# Genome assembly of wild tea tree DASZ reveals pedigree and selection history of tea varieties

**DOI:** 10.1038/s41467-020-17498-6

**Published:** 2020-07-24

**Authors:** Weiyi Zhang, Youjun Zhang, Haiji Qiu, Yafei Guo, Haoliang Wan, Xiaoliang Zhang, Federico Scossa, Saleh Alseekh, Qinghua Zhang, Pu Wang, Li Xu, Maximilian H-W Schmidt, Xinxin Jia, Daili Li, Anting Zhu, Fei Guo, Wei Chen, Dejiang Ni, Björn Usadel, Alisdair R. Fernie, Weiwei Wen

**Affiliations:** 1 Key Laboratory of Horticultural Plant Biology (MOE), College of Horticulture and Forestry Sciences, Huazhong Agricultural University, Wuhan, 430070 China; 2 Center of Plant Systems Biology and Biotechnology, 4000 Plovdiv, Bulgaria; 3 Max-Planck-Institute of Molecular Plant Physiology, Am Muehlenberg 1, 14476 Potsdam-Golm, Germany; 4 Council for Agricultural Research and Economics, Research Center for Genomics and Bioinformatics, Via Ardeatina 546, 00178 Rome, Italy; 5 National Key Laboratory of Crop Genetic Improvement, Huazhong Agricultural University, Wuhan, 430070 China; 6 Institute for Botany and Molecular Genetics, BioEconomy Science Center, RWTH Aachen University, 52074 Aachen, Germany; 7 Institute of Bio- and Geosciences, IBG-4: Bioinformatics, CEPLAS, Forschungszentrum Jülich, Leo- Brandt-Straße, 52425 Jülich, Germany; 8 Institute for Biological Data Science, Heinrich Heine University, 40225 Düsseldorf, Germany

**Keywords:** Genome evolution, Agricultural genetics, Genetic variation, Natural variation in plants

## Abstract

Wild teas are valuable genetic resources for studying domestication and breeding. Here we report the assembly of a high-quality chromosome-scale reference genome for an ancient tea tree. The further RNA sequencing of 217 diverse tea accessions clarifies the pedigree of tea cultivars and reveals key contributors in the breeding of Chinese tea. Candidate genes associated with flavonoid biosynthesis are identified by genome-wide association study. Specifically, diverse allelic function of *CsANR*, *CsF3’5’H* and *CsMYB5* is verified by transient overexpression and enzymatic assays, providing comprehensive insights into the biosynthesis of catechins, the most important bioactive compounds in tea plants. The inconspicuous differentiation between ancient trees and cultivars at both genetic and metabolic levels implies that tea may not have undergone long-term artificial directional selection in terms of flavor-related metabolites. These genomic resources provide evolutionary insight into tea plants and lay the foundation for better understanding the biosynthesis of beneficial natural compounds.

## Abstract (web summary)

Wild teas are considered as valuable resource for studying domestication and breeding. Here, Zhang et al. report genome of wild tea DASZ and transcriptome of 217 accessions, which clarify pedigree of Chinese tea cultivars and show tea may not have undergone long-term artificial directional selection on flavor-related metabolites.

## Introduction

Tea (*Camellia sinensis* (L.) O. Kuntze), a member of the genus Camellia (Theaceae), is one of the most popular beverages worldwide with rich flavors and health benefits^1,2^. It is known that tea drinking in China can be dated back to the 5th century AD, and by the end of the 6th century, the Chinese people began to have different cognitions of tea drinking: tea was no longer regarded as a mere medicinal drink, but also as a refreshing beverage^3^. The planting area of tea was more than 3.8 million hectares, resulting in the production of 6.1 million metric tons of tea worldwide in 2017 (http://www.fao.org). Research in tea science is very diverse, and is gradually verifying the folk wisdom concerning the health benefits of tea. Tea contains abundant medically important bioactive chemical compounds such as phenolics, amino acids, caffeine, and terpenes, which contribute to the pleasant flavors as well as high industrial and medical value of tea. For instance, Nakayama et al. showed that tea polyphenols such as Epigallocatechin gallate (EGCG) and theaflavin digallate (TF3) could inhibit the infection of influenza virus by binding to haemagglutinin^4,5^. EGCG can not only prevent the infection of influenza virus, but can also block the infectivity of other representative viruses such as HCV, HIV-1, and HBV^6^.

Tea plants are thought to be originated from the southwestern China including Yunnan and the adjacent provinces^3,7,8^. However, the wild progenitor of tea has not been found yet^7^. Analysis of the naturally occurring genetic variations can not only improve the understanding of the origin and domestication of certain species, but may also provide an effective way to decipher and determine the function of genes which can be utilized in the following up breeding practices. Since 2017, biological studies in tea plants have been greatly facilitated by the draft sequence assemblies of both a C. *sinensis* and a C. *assamica* variety^9–11^. However, a refined chromosome-scale reference genome is required for further facilitating both genetic research and trait improvement of tea. Wild teas grown in the aforementioned areas in China for over a 100 years are considered as valuable resources for basic research, since they have mainly undergone natural selection or have been only slightly affected by artificial selection. In addition, wild relatives or landraces, being often adapted to marginal environments, may possess desirable traits, such as the upright leaf angle in teosinte^12^ or superior stress tolerance and fitness in wild tomato^13^. Therefore, the genome sequencing of a wild relative or ancient tree would pave the way to unravel the evolutionary processes affecting the genome of tea plants as well as facilitate the mining of elite alleles.

Here, we present the genome of an ancient tea tree. Moreover, RNA sequencing of an additional 217 tea accessions is performed to characterize the genetic diversity, population structure, and pedigree relationship among a representative set of Chinese tea germplasms. We also demonstrate the utilization of the genome sequence and diverse natural populations of tea in the identification of genes and functional variations that regulate the content of catechins and gallic acid (GA) in tea leaves. These resources would facilitate the genetic improvement of tea plants as well as advance our understanding of the biosynthesis of health-beneficial natural products in tea.

## Results

### Genome sequencing and assembly and annotation

**Fig. 1:**
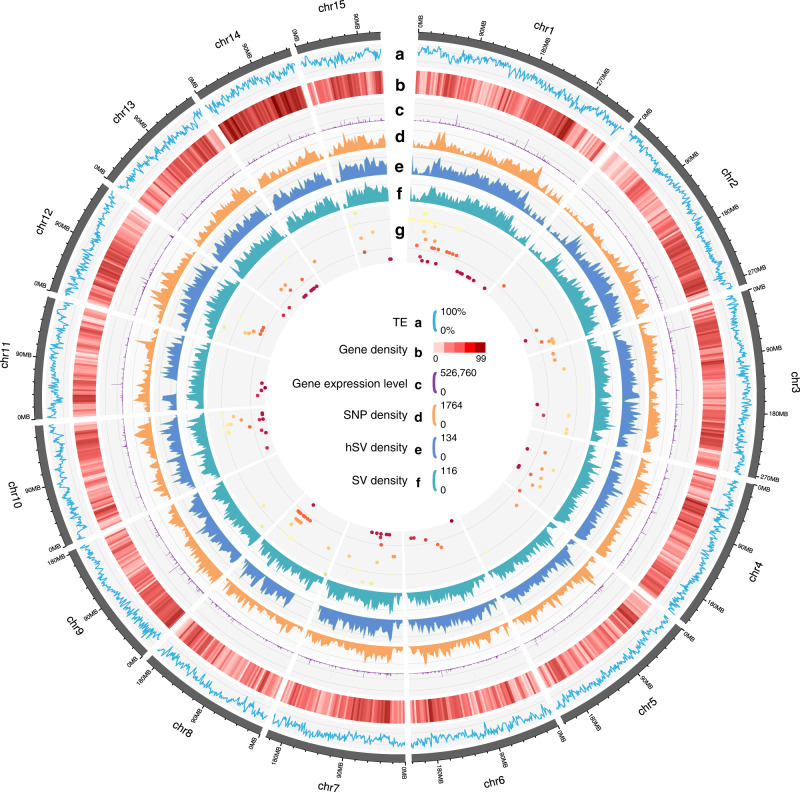
Features of DASZ genome and mQTL distributions. **a** Transposable-element content in each chromosome calculated using nonoverlapping window size of 1 Mb. **b** Gene density (numbers of genes in 1 Mb nonoverlapping windows). **c** Gene expression levels (sum of gene expression levels of each gene in 217 tea accessions). **d** SNP density (numbers of SNPs in 1 Mb nonoverlapping windows). **e** Density of heterozygous SVs (>50 bp) of DASZ (numbers of hSVs in 1 Mb nonoverlapping windows). **f** Density of SVs (>50 bp) identified between DASZ and Shuchazao genome (numbers of SVs in 1 Mb nonoverlapping windows). **g** mQTL distribution of catechins. Lines from outside to inner layers represent different catechins and each point indicates the mQTL identified by GWAS. Source data are provided as a Source Data file.

The genome of an ancient tea tree (named as DASZ; Supplementary Fig. 1) was sequenced and assembled using a combination of the single-molecule real-time (SMRT) sequencing technology from Pacific Biosciences (PacBio), next-generation sequencing technology, and high-resolution chromosome conformation capture (Hi-C). There were 5453 PacBio contigs linked by Hi-C, yielding a genome size of 3.11 Gbp with 1237 scaffolds and an N50 of 204.21 Mb, and 99.55% of the entire genome sequences were anchored on 15 chromosomes (Table 1; Supplementary Method 1; Supplementary Tables 1–6; Supplementary Figs. 2–6). 87.41% of the genome was found to be repetitive, including a large proportion (76.59%) of long terminal repeat (LTR) transposable elements (Fig. 1; Supplementary Figs. 7, 8; Supplementary Tables 7–9). *Gypsy* and *Copia* played dominant roles in LTRs, accounting for 49.36% and 8.50% of the DASZ genome, respectively (Supplementary Tables 7–9). The proportions of *Gypsy* and *Copia* were similar but slightly higher than the other two genomes of tea^9–11^. Considerable higher contents of other LTR sequences were detected in DASZ than Shuchazao (505,322,326 bp vs. 162,745,061 bp; Supplementary Tables 7–9)^10^, and this difference may be due to the higher Pacbio sequencing depth of DASZ, which could facilitate the prediction of long repetitive sequence.

**Table 1:** Summary statistics of DASZ assembly genome compared with that of Shuchazao and Yunkang 10.

Genomic feature	DASZ	Shuchazao	Yunkang 10
Length of largest scaffolds (bp)	336,926,639	7,310,000	3,505,831
Number of anchored scaffolds	15	/	/
N50 of scaffolds (bp)	204,210,641	1,390,000	449,457
Number of contigs	5453	/	258,790
Length of largest contig (bp)	16,831,527	/	257,648
Total length of contig (bp)	3,111,432,165	2,890,000,000	2,575,242,646
N50 of contigs (bp)	2,589,771	67,070	19,960
GC content (%)	38.98	/	42.31
Total length of pseudomolecules (Gb)	3.099	/	/
Sequences anchored to chromosomes (%)	99.55%	/	/
Number of unanchored scaffolds (bp)	1222	/	/
Total length of transposable elements (bp)	2,674,861,882	1,860,000,000	1,750,000,000
Percentage of transposable elements in genome size (%)	87.41	64.42	80.89
Numbers of gene models	33021	33,932	36,951
Average gene length (bp)	8050.5	7385	3549
Average exon number	5.38	5.7	4.8
Average exon length (bp)	211.7	259	237
Average CDS length (bp)	1139.09	1344.0	990
Number of annotated genes	30511 (92.4%)	31392 (92.51%)	33415 (90.4%)
Complete gene prediction BUSCO	93.2%	91.40% (86.2% in Scientific Data)	85.2%

There were a total of 33,021 high-confidence gene models in the tea plant genome (*C. sinensis*) as predicted by integrative gene-finding algorithms. Of these, 92.4% were functionally annotated in public databases (Supplementary Method 1; Supplementary Tables 10–12; Supplementary Figs. 9, 10). The completeness of the gene repertoire was evaluated using BUSCO v3.0. As a result, 93.2% of a core set of 1375 single-copy ortholog genes from plant lineage were complete in the assembly (87.9% as single-copy, and 5.3% as duplicates), reflecting the high quality of the assembly and annotations (Table 1; Supplementary Table 13).

### Structural variations between DASZ and Shuchazao

We remapped 100× coverage DASZ Pacbio sequences to the DASZ reference genome and 52,501 heterozygous structural variations (hSVs) longer than 50 base pairs (bp) were identified (ranging from 51 bp to 794,507 bp; Supplementary Data 1; Supplementary Method 2). Among the 52,501 heterozygous SVs, 2237 overlapped with gene coding regions (high impact SVs); whilst 1218 overlapped with the 5′ UTR and 795 overlapped with the 3′ UTR; and 6902 were in the intronic region (Supplementary Data 2). The high impact SVs affected a total of 2157 genes, which represented 6.5% of protein coding genes (Supplementary Data 2). GO annotation showed that about half of these high impact genes were related to the GO term “cellular process” and “metabolic process“ (Supplementary Fig. 11; Supplementary Method 2) and GO enrichment analysis showed that these genes were significantly enriched in the GO term of “regulation of cell death” (Supplementary Data 3; adjusted *P* = 4.44 × 10^−3^; Supplementary Method 2). A total of 27 genes were in the GO term “regulation of cell death” and detailed GO analysis showed that about half of them were related to membrane and had catalytic activity. Approximately 80% of them were related to the term “response to stimuli”. *W05g013121* and *W09g020593* (annotated as heme-binding protein and aspartyl protease, respectively) exhibited high expression levels in multiple tissues of DASZ, while most of 27 genes kept low expression levels in all the tissues (Supplementary Figs. 12, 13). We further evaluated hSVs in the Shuchazao genome by repeating the above analysis using Shuchazao Pacbio reads. In this genome, we identified a total of 1769 heterozygous and 21,003 homozygous SVs (92.2%), respectively (Supplementary Data 4). These results indicated greatly improved assembly quality of the DASZ genome.

SVs between DASZ and Shuchazao were further identified. After removal of the SVs overlapping with the homozygous ones identified in the DASZ reads, a total of 25,708 homozygous and 25,612 heterozygous SVs were identified (Supplementary Data 5). DASZ and Shuchazao shared 16,674 SVs. Among the 25,708 homozygous SVs between DASZ and Shuchazao, 1135 were high impact SVs that affected 1121 genes (Supplementary Data 6). We further performed GO annotation to these genes and the results were similar to the genes affected by hSVs in DASZ. Approximately 50% of 1121 genes were relating to the GO term “cellular process” and “metabolic process” (Supplementary Fig. 11; Supplementary Method 2). Detailed functional annotation revealed that 10 R genes were lost in Shuchazao, which may be involved in the response to biotic stress (Supplementary Data 6).

### RNA sequencing and variant identification of tea accessions

**Fig. 2:**
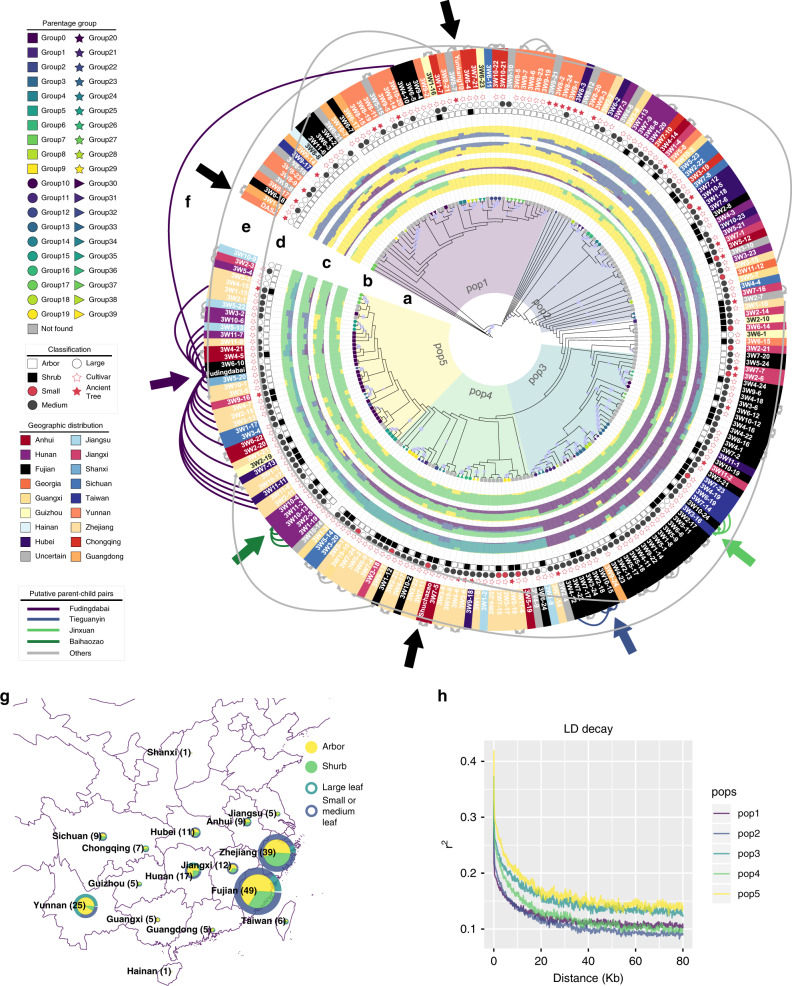
Population genetic analysis of 221 tea accessions. **a** Phylogenetic tree of 221 tea accessions. Circles in the middle of branches indicate the bootstrap values > 50 and the size of circles represent the values ranging from 50 to 100. The five colors in the background are the five subpopulations defined based on the phylogenetic tree and population structures. **b** Parentage analysis of 221 tea accessions. Different shapes and colors of nodes represent different parentage groups and the legend is shown in the left panel. Detailed information of these groups is presented in Supplementary Data 7. **c** Barplot of population structures. The inner layer to outside layer indicates the *K* from 2 to 5, respectively. Different colors represent different populations. **d** Classes of 221 tea accessions. Hollow and solid squares show the arbor or shrub of accessions, respectively. Hollow and black and red circles exhibit different leaf size and five-pointed stars represent the ancient trees or cultivars. Blankness indicates that the information is missing. Detailed legend is shown in left. **e** Geographic distribution of tea accessions. Colors represent the origins of 221 accessions. **f** Potential pedigrees of tea accessions. Black arrows indicate the three sequenced accessions. Colorful arrows represent the potential key contributors identified in this study. Links show the putative parent-children relationships. **g** Map of the geographic distribution of tea accessions. Radiuses of pie plots represent the numbers of accessions in one province of china. Inner parts of pie plots indicate the ratio of arbor and shrub accessions. Outside layers of pie plots show the ratio of different leaf size. **h** LD decay of five subpopulations. Different colored lines show different subpopulations. Source data underlying **a**, **c**, **f**, **h** are provided as a Source Data file.

RNA sequencing was conducted on 217 tea accessions, 216 of which were collected from 16 provinces of China and one was collected from Georgia (Supplementary Data 7, 8). Among these accessions, 31 are ancient trees, 162 are registered cultivars, and 24 are of uncertain identity. Based on field investigation, the 217 accessions were classified into four distinct groups (i.e., small- or medium-leaf group, large-leaf group, shrub group, and arbor group). Arbor and small- or medium-leaf types were the dominant types in most of the 16 provinces (Fig. 2g; Supplementary Data 7), while most of the accessions from Yunnan province belonged to large leaf types.

In order to exam the accuracy of SNP calling and filtration, we randomly picked 500 SNPs and two accessions for Sanger sequencing. The results demonstrated that the accuracy of SNP calling was 98.5%. These single nucleotide polymorphisms (SNPs) covered 81.1% of the annotated genes of DASZ, and 1491 SNPs were predicted as high impact SNPs (Supplementary Table 14). The heterozygous rates for each SNP among these 217 accessions ranged from 0 to 100% with an average of 26.0%, indicating a high heterozygosity within the tea population.

For a more comprehensive analysis of natural tea population collected here, we re-sequenced Fudingdabai, an elite tea variety in China (arbor with medium leaf size).

### Parentage analysis

We first performed parentage analysis of 221 tea accessions using ten sets of 500 randomly selected high polymorphic information content (PIC > 0.35) biallelic variants. A total of 101 accessions were found to have potential parents, among which 27 were found in pedigree records (Supplementary Data 9). We further merged these putative parent–offspring pairs into 40 groups. Fudingdabai was predicted as the parent of 19 offsprings, among which 13 were consistent with the pedigree records (Fig. 2b, f ; Supplementary Data 9). It is worth noting that accessions related with Fudingdabai were collected from many different provinces in China, indicating that Fudingdabai was one of the most widely used parents for tea breeding (Fig. 2e; Supplementary Data 7, 9). Another three accessions were predicted as the parents of at least three accessions and all of them were the contributors in some local breeding programs (Fig. 2f; Supplementary Data 7, 9). For example, an elite variety Tieguanyin (3W2-18) from Fujian province had three putative offspring with two being found in the breeding records, and all of the predicted offspring were from Fujian province (Fig. 2e; Supplementary Data 7, 9). DASZ was the putative offspring or parent of Fenghuangshuixian, which is a 300–400-year-old ancient tree according to the breeding records (Fig. 2f; Supplementary Data 7, 9). In order to ensure the randomization of sampling, we compared our result with previous parentage analysis which selected 128 elite tea accessions and discovered that Fudingdabai played important role in Chinese tea breeding (the potential parent of 29 tea accessions)^14^. And 15 of these 29 accessions were not included in our study. The importance of Fudingdabai in the Chinese tea breeding is therefore strongly indicated by these two independent studies.

### Population structure and diversification of tea accessions

Most accessions in the same parentage group were clustered together in the phylogenetic tree, while a few accessions from different parentage groups clustered together (Fig. 2a). On the one hand, accessions collected here were not completely grouped by leaf size or tree shape, especially for the Fudingdabai family, which included all the four kinds of phenotypes with uniform distribution (Fig. 2d). On the other hand, the accessions with both arbor and large-leaf phenotypes apparently clustered together in the Yunnan clade. Surprisingly, we did not observe distinct separation between ancient trees and cultivars (Fig. 2d). Only a subset of the accessions collected from the same provinces were grouped together and most of accessions from different provinces were mixed together in the phylogenetic tree. Similar results were also obtained in principal component analysis (Supplementary Fig. 14). Most accessions from the same parentage group were clustered together and accessions harboring the same phenotype or collected from the same place were scattered across the PCA plot (Supplementary Fig. 14). The phylogenetic analysis and PCA results indicated that the tea accessions collected here were mainly distinguished by their kinship rather than their geographical origin or morphological characteristics such as tree shape or leaf size. Nevertheless, it was still observed that most of the accessions from Yunnan province were further apart from other accessions in the PCA plot (Supplementary Fig. 14).

We further estimated the ancestry of these tea accessions in a model-based manner using a different cluster number (K) within ADMIXTURE^15^. Partially consistent with the parentage analysis, most accessions showed an admixture ancestry model. The accessions which were predicted as potential key contributors displayed much lower proportions of admixture than other accessions (Fig. 2c). Based on the phylogenetic and population structure analysis, nine accessions showing an extreme admixture background were removed from this analysis (Fig. 2c), and the remaining 212 accessions were divided into five subpopulations (Fig. 2a). Interestingly, all five subpopulations showed similar and rapid LD decay ranging from 5 to 9 kb (Fig. 2h).

### Variation of catechins and gallic acid in tea accessions

**Fig. 3:**
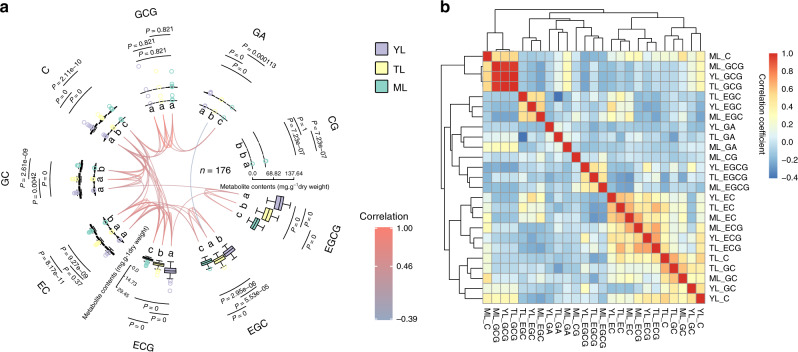
Catechin and gallic acid profiles of tea population. **a** Catechin and GA contents of tea accessions in different leaf tissues. The outside boxplots indicate the contents of catechins in three different leaf samples (YL: young leaf; TL: third leaf; ML: mature leaf; mg g^−1^ dry weight). The median is indicated by the middle line of the box, the lower and upper boundaries show the first (25%) and third (75%) quartiles and the whiskers represent the minimum and maximum values, excluding outliers. The points outside the whiskers represent the outliers. Letters represent the significant levels of posthoc (adjusted *P* < 0.01; Tukey’s test; two sided). Links indicate the significant correlations among catechins and colors show the correlation coefficients. **b** Heatmap of correlation of catechins and gallic acid in different leaf samples.

We measured eight catechin compounds and GA (CG: catechin gallate; GA: gallic acid; GCG: gallocatechin gallate; C: catechin; GC: gallocatechin; EC: epicatechin; ECG: epicatechin gallate; EGC: epigallocatechin; EGCG: epigallocatechin gallate) in three different leaf samples (YL: young leaf; TL: third leaf; ML: mature leaf) of 176 tea accessions (Supplementary Data 10). Among the nine compounds, EGCG, EGC, and ECG were at much higher levels than other compounds in all three samples (Fig. 3a; Supplementary Data 10). GCG, GA, and CG were extremely low in all the three samples of 176 tea accessions (Fig. 3a; Supplementary Data 10). Out of the nine compounds, five showed significantly higher levels in YL compared with in TL and ML, and seven exhibited significantly higher levels in TL than in YL (*P* < 0.01; Tukey’s test; Fig. 3a; Supplementary Method 3). In general, the catechin content decreased with increasing leaf age. High positive correlations amongst these nine compounds were detected in the three samples, particularly among EC, ECG, and GC, suggesting that these three catechins may share similar regulatory networks (Fig. 3a, b).

Tukey’s test showed that several compounds were affected by leaf size or subpopulation (Supplementary Table 15; Supplementary Method 3). However, no significant difference was detected between arbor and shrub types. EGCG was significantly higher in ancient trees (Supplementary Table 15) in Tukey’s test but this significance was not detected in mixed linear regression. Contents of Catechin in YL and TL showed differences among different subpopulations, as identified by both Tukey’s test and mixed linear model (Supplementary Tables 15, 16; Supplementary Method 3).

### Evolutionary analysis of gene families

**Fig. 4:**
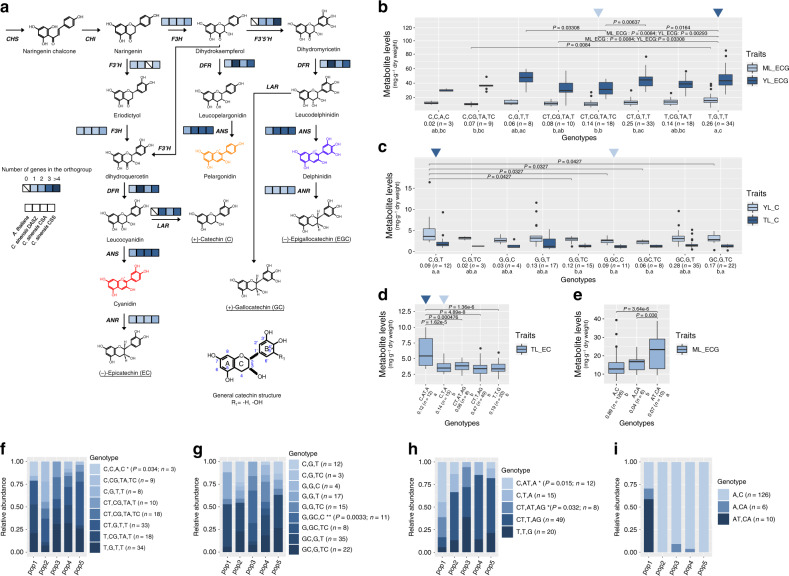
Catechin biosynthesis pathway and identification of favorable genetic variants of candidate genes. **a** General scheme for the biosynthesis of non-galloylated catechins in *Camellia* species. The main catechins accumulating in *Camellia* species are underlined. Catechins are essentially the products of reduction, catalysed by ANR or LAR, of either leuco- or anthocyanidins. For the main metabolic steps, the number of genes contained in the respective orthogroup from *A. thaliana*, *C. sinensis* (DASZ), *C. sinensis* (CSA), and *C. sinensis* (CSS) is indicated by the colored boxes. Gene names are abbreviated in black. *CHS* chalcone synthase, *CHI* chalcone isomerase, *F3H* flavanone 3-hydroxylase, *F3*′*5*′*H* flavonoid 3′5′-hydroxylase, *F3*′*H* flavonoid 3′-hydroxylase, *DFR* dihydroflavonol 4-reductase, *ANS* anthocyanidin synthase, *LAR* leucoanthocyanidin reductase, *ANR* anthocyanidin reductase. **b**–**e** Boxplots of the genotype (each SNP site is separated by comma, and N/N indicates heterozygous SNPs) analysis using non-synonymous SNPs in the candidate genes for *W12g026224* (*CsF3*′*5*′*H*), *W08g018649* (*CsANR*), *W12g025045* (*CsMYB5*), and *W13g026373* (*CsTT2*), respectively. The middle line of the box represents the median, the first (25%) and third (75%) quartiles are indicated by the lower and upper boundaries and the whiskers show the minimum and maximum values, excluding outliers. The black points outside the whiskers indicate the outliers. The first line of xlab shows the genotypes and the second line indicates the frequency of the corresponding genotypes in tea population. The last line shows the significant levels obtained by Tukey’s test (adjusted *P* < 0.05; two sided). Dark blue and light blue triangles in (**b**–**d**) indicate the selected genotypes for further functional validation. **f**–**i** Stack barplots of genotypes of *CsF3*′*5*′*H*, *CsANR, CsMYB5*, and *CsTT2* distribution among tea subpopulations, respectively. Each color in barplot indicates one genotype. The asterisks after the genotypes represent the significant levels of fisher exact test (*P < 0.05; **P < 0.01; Fisher exact test; two sided). Source data underlying (**b**–**i**) are provided as a Source Data file.

Using large-scale comparative analysis between the full set of proteins encoded in the genomes of 15 plant species, from both monocots and eudicots, we next investigated the dynamics of gene family expansion and contraction, focusing on the differences emerging in the size of the orthogroups of catechin biosynthesis (Supplementary Method 4). Catechins derive from naringenin through various steps of the phenylpropanoid pathway, and are eventually formed from the reduction of either a leuco- or anthocyanidin substrate. They represent up to 80% of the total polyphenol content in tea leaves; of these, around 70% are esterified with a unit of GA in position 3 of the aglycone backbone^10^. The whole pathway of catechin biosynthesis involves 11 gene families (*CHS*, *CHI*, *F3H*, *F3*′*5*′*H*, *F3*′*H*, *DFR*, *LAR*, *ANS*, *ANR*, *UGGT*, and *SCPL*-acyltransferases), whose members cluster into 23 different orthogroups (Supplementary Data 11; Fig. 4a). The genes catalyzing the early steps of the pathway (e.g., *CHS*) are generally dispersed across more than one orthogroup, likely as a result of their functional specialization toward a wider range of substrates, reflected in a greater amino acid divergence^16^. In the orthogroups of the genes active in the late steps of the pathway, tea genomes generally show a higher gene copy number with respect to Arabidopsis (*ANS, DFR, LAR,* and *F3*′*5*′*H*).

When comparing the copy number of catechin biosynthesis genes against the predicted size of the gene families in the ancestral nodes, we were able to identify specific cases of gene family contraction and expansion (Supplementary Data 11; Supplementary Method 4). Polyphenol oxidases (PPOs, belonging to OG0000385, and representing the orthologs of *tt10* in *Arabidopsis*^17^, for example, underwent contraction in the terminal branch leading to DASZ loss of 4 genes (adjusted *P* = 0.00075138); the number of PPO genes remained instead relatively constant in other tea lineages (Supplementary Fig. 15). Similarly, also the orthogroup containing SCPL acyltransferases (OG0000034, which includes, among others, the genes involved in the synthesis of galloylated catechins), was subjected to a contraction in DASZ, with loss of five genes (adjusted *P* = 0.000194233). The gene family of SCPL acyltransferases has instead expanded in *C. sinensis* var. *sinensis* (CSS), with the net gain of eight additional orthologs (adjusted *P* = 4.30226 × 10^−7^; Supplementary Figs. 16, 17; Supplementary Data 11–13). These contrasting evolutionary dynamics of gene gain and loss in the tea lineages are also exemplified by the fate of the genes encoding UDP-glycoside transferases of the UGT73 subfamily (OG0000017, encoding flavonol-7-O-glycoside transferases): this orthogroup accumulated significant expansion along the branch predating the divergence of the three tea genomes (+13 genes, adjusted *P* = 0.00261188), and additionally underwent contrasting gain and losses along the species-specific branches (*C. sinensis* var. *sinensis* (CSS) and DASZ acquired nine and eight UGT73 genes, respectively (adjusted *P* = 9.18156 × 10^−5^ for CSS and 0.00111415 for DASZ), while Yunkang 10 had a loss of 17 UGT73 orthologs, adjusted *P* = 3.1353 × 10^−13^; Supplementary Data 11, 13).

In addition to the analysis of the orthogroups undergoing significant changes in the catechin biosynthesis, we also had a wider look at the specific gene families undergoing contractions or expansions exclusively in the DASZ lineage (Supplementary Fig. 18; Supplementary Data 14; Supplementary Method 4). When looking at the intersections of all rapidly evolving orthogroups in the subclade comprising *A. chinensis* and the three tea genomes, we could identify the gene families specifically evolving in DASZ only, and at the same time not rapidly evolving in the other internal or terminal branches (Supplementary Data 14). In general, the DASZ-specific orthogroups undergoing expansions contained members encoding components of cell signaling and division, and cell wall metabolism, while orthologs encoding cytoskeleton and primary metabolism (amino acid and polyol metabolism) were instead part of gene families undergoing significant contractions (Supplementary Data 14). Taken together, these results suggest that, despite the short evolutionary distance separating DASZ from the other teas, distinctive dynamics of gene gain and loss affected the genome of DASZ; these events may have certainly contributed to lineage sorting marking the diversification of the *assamica*, *sinensis*, and DASZ teas from their last common ancestor^18^.

### Identification of genes in catechin biosynthesis

A total of 176 loci associated with the content of seven catechins and GA in three different leaf samples were identified using genome-wide association mapping (*P* < 1.05 × 10^−5^; Fig. 1g; Supplementary Data 15). Loci that encode key enzymes in catechins biosynthesis were found, including *ans*, *anr*, and *f3’5’h*. A strong association signal of ECG content in both ML and YL was mapped to locus *f3*′*5*′*h* at the end of chromosome 12 (*P* = 8.18 × 10^−6^; Supplementary Data 16). One possible candidate gene at this locus was *W12g026224* (flavonoid 3′, 5′-hydroxylase), which belongs to the CYP75A superfamily and could catalyze the hydroxylation of naringenin, eriodictyol, dihydrokaempferol, and dihydroquercetin to form 3′4′- and/or 3′4′5′-hydroxylated products (Fig. 4a)^19^. The association of this gene with the content of ECG is consistent with the enzymatic role of its encoded protein: F3′5′H uses in fact dihydrokaempferol (one of the precursors of ECG; Fig. 4a) as a preferred substrate to convert it to dihydromyricetin, from which different catechin monomers are formed (i.e., EGC and GC).

The *anr* locus on chromosome 8 (*W08g018649*) was associated with the catechin content in young leaves. This gene encodes an anthocyanidin reductase and could function in the production of terminal flavan-3-ol monomers required for the formation of proanthocyanidins or condensed tannins (*P* = 7.5 × 10^−7^; Supplementary Data 16; Fig. 4a)^20^. Moreover, the *ans* locus on chromosome 13 (*W13g027058*) was associated with the epicatechin content in mature leaves, and *ans* encodes an anthocyanidin synthase with leucoanthocyanidin dioxygenase activity (*P* = 3.48 × 10^−6^; Supplementary Data 16; Fig. 4a)^21^. The gene *W10g022427* located in an mQTL for GA contents in TL on chromosome 10 was annotated as a HXXXD-type acyl-transferase-like protein, which was predicted to catalyze the modification of *p*-coumaroyl CoA and shikimate to chlorogenic acid (*P* = 3.88 × 10^−6^; Supplementary Data 16). *P*-coumaroyl CoA is a pivotal metabolite located at the junction of flavonoid and phenylpropanoid pathways^22^.

The loci encoding transcription factors which may regulate catechin biosynthesis were also discovered. For instance, strong association signals were mapped to the loci containing *myb* genes, which have been demonstrated to have regulatory effects on flavonoid and anthocyanin levels in a wide range of species^23–26^. Two candidate genes *W12g025045* and *W13g026373*, which were both annotated as *myb* family members, showed significant association with EC in TL and ECG in ML, respectively (*P* = 3.54 × 10^−7^; *P* = 8.97 × 10^−6^; Supplementary Data 16). *W12g025045* is the ortholog of *AtMYB5* whilst *W13g026373* is the ortholog of *AtTT2*, both of which have been demonstrated to be involved in the formation of proanthocyanidins in Arabidopsis^27^.

### Favorable genetic variants of candidate genes

A total of ten non-synonymous mutations of *W12g026224* (*F3*′*5*′*H*) were identified based on RNA-seq data. Four SNPs (MAF > 0.05; *P* < 0.001; ANOVA) were significantly associated with ECG in both YL and ML, and formed eight different genotypes with the sample size ≥ 3 in tea population. Further analysis revealed that T/CGTT (N/N indicates heterozygous SNPs) conferred high level of ECG content (Fig. 4b). We found that CCAC, which conferred slightly lower ECG content than T/CGTT, was significantly enriched in pop1, indicating that CCAC may be related to the differentiation between pop1 and other subpopulations (*P* < 0.05; fisher’s exact test; Fig. 4f). Eight non-synonymous SNPs were identified in ANR, three of which showed significant association with catechins in TL and YL (*P* < 0.05; ANOVA). Further genotype analysis revealed that the level of catechins was significantly higher in CGT than in GG/CC and GG/CT/C genotypes in YL (*P* < 0.05; Tukey’s test; Fig. 4c) and GG/CC was significantly enriched in pop3 (*P* < 0.05; fisher’s exact test; Fig. 4g). *CsMYB5* (*W12g025045*) harbored three non-synonymous SNPs, all of which were significantly associated with EC in TL (*P* < 0.001; ANOVA). EC contents were significantly higher in CA/TA genotypes than in other genotypes (*P* < 0.05; Tukey’s test; Fig. 4d). It was further found that CA/TA and C/TA/TA/G genotypes were significantly enriched in pop1 and pop3, respectively (*P* < 0.05; fisher’s exact test; Fig. 4h). Seven non-synonymous SNPs were identified in the coding region of *W13g026373*, two of which showed significant association with ECG contents in ML. A/TC/A genotypes had significantly higher ECG levels than AC or AC/A genotypes (*P* < 0.05; Tukey’s test; Fig. 4e), and were only present in pop1 (Fig. 4i).

**Fig. 5:**
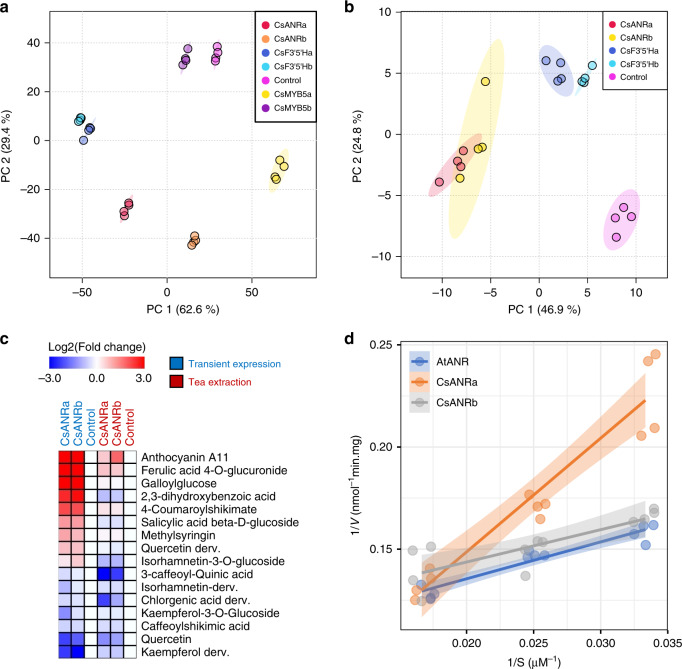
Functional analysis of three candidate genes. Allele of *CsANRa*: CGT; Allele of *CsANRb*: GCC; Allele of *CsF3*′*5*′*Ha*: TGTT; Allele of *CsF3*′*5*′*Hb*: TCAC; Allele of *CsMYB5a*: CAA; Allele of *CsMYB5b*: CTA. **a** Transient overexpression of these three genes was conducted in tobacco leaves in which secondary metabolites were subsequently detected by LC–MS. The metabolite profiling of all the overexpression tobacco lines is different from that of the GFP control. The metabolite profiling of the two alleles of each gene is distinct from each other. **b** The purified enzymes were incubated with tea extracts revealing their catalytic function in tea secondary metabolism. The dots with the same color in (**a**) and (**b**) indicate the four biological replicates for metabolite profiling. **c** Heatmap of significantly changed metabolites in both tobacco leaves (left) and tea extract (right) with presence of *CsANR* (*P* < 0.05; *t* test; two sided). The free GFP was used as negative control. **d** Enzyme activity of the anthocyanidin reductase (ANR). The Arabidopsis ANR (*AtANR*) was used as assay control. Four measurements were conducted for each enzyme under each concentration of cyanidin chloride. Data points are jittered to improve legibility. Shadows indicate the 95% confidence interval of linear regression. Two independent experiments were performed and generated similar results. Source data underlying **a**, **b**, **d** are provided as a Source Data file.

Functional validation of *CsANR*, *CsF3*′*5*′*H*, and *CsMYB5* were furthermore conducted (Supplementary Method 5). Because each gene harbored multiple alleles, for simplicity, based on their contribution to the metabolite contents we selected two alleles with high (allele a) and low (allele b) level of contribution for further functional validation, respectively (Fig. 4b–d; *CsANRa*: CGT; *CsANRb*: GCC; *CsF3*′*5*′*Ha*: TGTT; *CsF3*′*5*′*Hb*: TCAC; *CsMYB5a*: CAA; *CsMYB5b*: CTA; sequences of these alleles are in Supplementary Data 17; Supplementary Table 17). Both alleles of each gene were transient expressed in tobacco leaves with the free GFP as negative control. Both alleles of *CsANR*, *CsF3*′*5*′*H*, and *CsMYB5* affected metabolite profiles of the over-expressed tobacco lines when compared with GFP control in vivo (Fig. 5a). In addition, the CsANR and CsF3′5′H proteins were purified by affinity purification and incubated with tea extract for 30 min. As a result, metabolites including flavonoids such as anthocyanin A11, epicatechin and procyanidin were significantly changed by the CsANR and CsF3′5′H complexes (Fig. 5b, Supplementary Data 18). Moreover, the CsANRa and CsANRb converted several flavonoid related compounds in both in vivo and in vitro experiments (Fig. 5c, Supplementary Data 17). The enzyme catalytic parameters were characterized in purified CsANRa, CsANRb using the Arabidopsis AtANR enzyme as a control. The CsANRa has at least ten times higher Km and three times lower enzyme efficiency compared with both CsANRb and AtANR (Fig. 5d; Supplementary Table 18). The lower enzyme efficiency of CsANRa compared with CsANRb may partially block the pathway of cyanidin biosynthesis, which in turn would be anticipated to lead to the observed accumulation of catechin in the lines expressing this allele (Fig. 4a).

## Discussion

Tea has been grown in southwest China for ~5000 years and is largely cultivated worldwide^28^. Wild teas have mainly undergone natural selection or are only slightly affected by artificial selection, and thus are considered as a valuable resource for studying the genomic and phenotypic changes accompanying tea domestication and as a source of alleles for current tea breeding programs. The genome reported here will facilitate a better understanding of the high genetic diversity and kinship (breeding history) within the extant tea germplasms. It also lays the foundation for elaborating the intricate structural and regulatory aspects in the biosynthesis of the most bioactive compounds in tea.

Our results demonstrate that several elite Chinese tea varieties such as Fudingdabai have played crucial historical roles in Chinese tea breeding programs. However, diverse natural genetic variations remain untapped with regard to the genetic improvement of tea plants. In addition, third-generation sequencing identified large-scale structural variations between our assembled, chromosome-anchored genome (DASZ) and the previously published genome of the elite CSS variety Shuchazao. Structural variations have been demonstrated to have the potential to manipulate gene expression by affecting regulatory elements and chromatin loops in other species^29^. Recent studies have shown a low linkage disequilibrium between SVs and SNPs, indicating that SVs play independent roles from SNPs^30,31^. A total of 52,501 heterozygous SVs were identified in DASZ. However, only 25,612 hSVs were detected in Shuchazao. The huge difference of hSV number between DASZ and Shuchazao may result from several factors. On one hand, different number of hSVs may be partially caused by different Pacbio sequencing depth, which was less in Shuchazao than DASZ. On the other hand, a previous study showed that population history and clonal propagation tended to accumulate recessive deleterious variants in a heterozygous state^32^. Shuchazao was selected from local varieties of Shucheng County in 1990s^33^, while as an ancient tree that has lived several hundred years DASZ was more inclined to hide the SVs in a heterozygous state compared with Shuchazao. Further GO analysis suggested that genes affected by hSVs in DASZ were enriched in the term of “regulation of cell death”. These results are similar to the results obtained in grape^30^. It will be interesting to further investigate the functional effects and mechanism underlying the emergence of hemizygous genes in the future.

There seem to be frequent genetic exchanges between different groups (e.g., ancient trees and cultivars; accessions with *sinensis* and *assamica* characteristics) of tea plants since no clear separation was observed between them. Compared with the wild species, cultivated germplasms usually show lower levels of genetic diversity because breeding practices tend to reduce genetic diversity to a greater extent than domestication^34,35^. However, a recent study showed a considerably higher level of genetic diversity in cultivated tea than in wild-type tea, which could probably be attributed to factors including incomplete sampling of wild teas and the existence of wild teas in the cultivation^36^. Based on the above facts, it can be speculated that unlike some other species such as tomato^37^, tea has not undergone long-term artificial directional selection, at least not in terms of metabolites that confer the flavor. First, tea leaves are usually consumed after processing with the aim to produce the target flavor based on the intrinsic components in the leaves of different accessions. The quality or flavor of tea can thus be influenced and improved by the processing techniques. Secondly, there are various tea products to cater the diverse tastes of different consumers, such as green tea, black tea, white tea, yellow tea, dark tea, oolong tea, and pu’er tea, which are all processed by different protocols. Diverse flavors demanded by different consumers may lead to diversified breeding goals. Thirdly, the self-in-compatible characteristic of most tea varieties may not offer opportunities for long-term artificial selection, and conventional breeding strategies such as backcrossing and selfing are not feasible for most accessions in *C. sinensis*^38^. Finally, given that leaves from ancient tea trees that are several 100 years old are still used for processing tea product today^33^, it is possible that unlike for many other domesticated crops strong selection for certain types of taste and/or flavor may not have occurred in tea. That said wild teas are rare, not well identified and perhaps many of those accessions that we call wild teas are actually from feral populations. So this higher level of genetic diversity in cultivated tea might be due to incomplete sampling of real wild teas, or to a cultivated germplasm which is mostly deriving from a few controlled crosses between accessions of different geographic origin.

As the primary flavonoids in tea plants, catechins largely determine the astringency of tea flavor, and the protective effects of tea catechins on human health have been documented; thus, continuous efforts have been made to study the biosynthesis of this kind of beneficial compounds^39,40^. Transient overexpression of genes identified by GWAS in this study (*CsANR*, *CsF3*′*5*′*H*, and *CsMYB5*) in tobacco together with in vivo and in vitro enzymatic assay verified their diverse allelic function in flavonoid and anthocyanin biosynthesis. Understanding of the natural allelic variants could also provide implications for the utilization of functional genes in subsequent breeding practices or metabolic engineering. Here, the high-quality reference genome, coupled with the natural population, could greatly facilitate the study of valuable and specialized metabolites in tea plants. Besides, the favorable alleles obtained from our data can be used to enhance the production of catechins through bioengineering.

In conclusion, we have generated a high-quality chromosome-scale reference genome of an ancient tea tree, which may promote the advance of tea plant genomics and genetic improvement. The collected population and metabolite profiling in tea plants provide an example for the utilization of this genome to dissect complex metabolic traits. Favorable alleles or haplotypes for the content of catechins as well as the active components determining the quality and health attributes of tea were discovered in this study, which may be used to facilitate future breeding programs and improvement of the biosynthesis of beneficial natural products.

## Methods

### Plant materials and sequencing assembly

An ancient tea tree (named as DASZ) found in wild condition (Mangyan Village, Yongjiang Township, Longyang District, Baoshan City, Yunnan Province; N 24°54′55.59″ E 98°48′30.87″; the altitude is between 1000 and 2000 m; Supplementary Fig. 1) was sampled for whole genome sequencing and de novo assembly. Five 20K de novo libraries were constructed according to standard manufacturers’ protocol (SMRT PacBio) to be used for SMRT PacBio genome sequencing. The final libraries were sequenced on the PacBio Sequel platform (Pacific Biosciences). A total of 39,763,171 reads were generated from 54 cells of PacBio data with a total length of 361 Gbp. Falcon (v0.3.0)^41^ was performed to assemble the PacBio data. The HaploMerger2 (v20180603)^42^ program (default parameters) was used to reduce the redundancy, and Arrow program (https://github.com/PacificBiosciences/SMRT-Link) with default parameters was used for correcting the sequencing errors according to the alignments. DNA was also sequenced using the Illumina HiSeq platform and a total of 200 Gbp clean data were generated. Clean reads were mapped to the corrected above-mentioned contigs with BWA MEM (v0.7.16a)^43^ with default parameters, and high-quality mapped reads (MAQ >20) were further used to polish the assembly using Pilon (v1.22)^44^ with default parameters. This procedure resulted in a total assembly length of 3.11 Gbp with an N50 length of 2.59 Mbp.

### Hybrid assembly of PacBio contigs

Details of the Hi-C library construction procedure are described in the Supplementary Method 1. Two libraries with an insertion size of 300 bp were sequenced on the Hiseq Xten instrument, resulting in the generation of 466.2 Gbp raw data. After filtering of the data, 390.2 Gbp clean data were retained. The clean reads from sequenced Hi-C libraries were mapped to draft contigs genome for obtaining global mapped reads using bowtie2 (v2.2.5)^45^. Unique mapped read pairs were selected for obtaining valid interaction pairs under HiC-Pro pipeline (v2.5.0)^46^. After removal of the duplicated pairs, 36.1 Gbp effective bases were obtained for further analysis. Unique valid interaction pairs from each library were merged for interaction-matrix construction. Juicer (v1.0)^47^ and 3d-dna (v2.0)^48^ were applied to group, sort and target the contigs of draft genome, and finally evaluate the assembly results.

### Genome annotation

Repetitive families in the genome were identified using an integration of independent homology searching and de novo predictions. We used RepeatMasker^49^ and ProteinMask^49^ for homology searching and de novo predictions were conducted by RepeatModeler^50^ and LTR-FINDER^51^. Tandem repeats were discovered by Tandem Repeats Finder v4.09^52^.

To aid the gene annotation, total RNA of ten different tissues from “DASZ” was sequenced using Illumina HiSeq platform. RNA was extracted using TRIZOL protocol and the libraries were constructed using the Illumina standard mRNA-seq library preparation kit. In addition, two PacBio ISO-Seq libraries were constructed for a sample pooled from the above-mentioned ten tissues and subsequently sequenced on the PacBio Sequel platform (Pacific Biosciences). Then, Augustus (v3.3.1)^53^ and SNAP (v2006-07-28)^54^ were used for de novo prediction with the self-trained parameters. Finally, evidence of de novo prediction, homolog based prediction and EST/transcripts prediction was submitted to MAKER (v2.31.10)^55^, resulting in the final gene models.

### RNA sequencing and variant calling of tea accessions

Accessions for RNA sequencing and metabolic profiling were grown in a single field at Huazhong agricultural university, Wuhan, China. We harvested the young leaf samples for each accession on the same day. Total RNA was extracted using extraction kit (Huayueyang, Beijing, China) according to the manufacturer’s instructions. The stranded RNA libraries were constructed using the KAPA Stranded mRNA-Seq Kit (Roche, Pleasanton, CA) following the manufacturer’s recommendations. After library preparation and pooling, we sequenced the libraries using pair-end sequencing with 150 bp reads on an Illumina HiSeq X instrument (Illumina, San Diego, CA).

After trimming the low quality reads using Trimmomatic-0.36^56^ with default parameters, the clean RNA-seq reads were mapped to reference genome using STAR^57^ with 2-pass-mode. PCR duplicates were removed using picard (http://broadinstitute.github.io/picard/). GATK^58^ SplitNCigarReads tool was used to update cigar string in vcf file. SNP calling was carried out by GATK^58^ HaplotypeCaller with—dont-use-soft-clipped-bases and—standard-min-confidence-threshold-for-calling 20 options. High-quality variants were obtained by the following steps: (1) raw SNPs were filtered by GATK^58^ VariantFiltration (QD < 2.0 ||FS > 60.0|| MQ < 40.0 ||MQRankSum < −12.5|| ReadPosRankSum < −8.0). (2) SNPs with more than two alleles were filtered. (3) For homozygous SNPs, the supporting reads should be more than 10. (4) For heterozygous SNPs, the sequencing depth for each allele should be greater than 4. (5) SNPs within 10 bp of InDels were removed. (6) SNPs with missing rate > 0.8 and MAF < 0.05 were removed. Finally, the sites that failed to pass these criteria were assigned as missing data.

### Parentage analysis

SNPs with missing rate ≤ 10% and MAF > 0.05 were selected to calculate PIC (polymorphic information content) by Cervus 3.0^59^. To improve the computational efficiency, we randomly chose ten sets of 500 SNPs with PIC greater than 0.35 for further analysis. Parentage analysis was conducted by Cervus 3.0^59^. All the tested accessions were considered as candidate mothers. The level of confidence in the parentage analysis was estimated via simulation with the following parameters: number of offspring = 10,000, number of candidate mothers = 221, proportion of sample = 0.5, proportion of mistyped loci = 0.01, and proportion of loci typed = 0.8. The most-likely parent–offspring pairs were assigned by Cervus 3.0^59^ with confidence of 95% and parent–offspring pairs identified at least in nine sets of markers were considered as the reliable pairs.

### Population genetic analysis

SNPs with missing rate ≤ 10% and MAF > 0.05 were selected to perform PCA, population structure analysis and phylogenetic analysis. SNPs were further filtered by PLINK^60^ software to mitigate the effect of LD with the parameter:—indep-pairwise 50 10 0.1. PCA was conducted by smartPCA in EIGENSOFT packages^61^ and the first two components were selected for plotting with R. Population structure was analyzed by ADMIXTURE software^15^. We ran ADMIXTURE with cross-validation for *K* values from 2 to 10. For each *K*, 20 independent runs of ADMIXTURE were performed. Values of membership coefficient (*Q* matrix) were obtained using CLUMPP v1.1.2^62^. Based on the cross-validation error, we choose five as the best *K*. Stacked barplot of *Q* matrix was generated in R^63^. Phylogenetic tree was constructed by RAxML software^64^ with the parameter: x (rapid bootstrap random number seed) = 12345, p (parsimony random seed) = 12345, # (number of runs) = 100, m (Model of Binary) = GTRGAMMA, and f (rapid Bootstrap analysis and search for best-scoring ML tree in one program run) = a. Phylogenetic tree was displayed by iTOL^65^. LD decay was analyzed by PopLDdecay software^66^ using the following parameters: Miss (Max ratio of miss allele filter) = 0.5, MAF (Min minor allele frequency filter) = 0.05, MaxDist (Max Distance (kb) between two SNP) = 100. LD decay was defined as the physical distance of the dropping of LD to half of the maximum.

### Genome-wide association analysis

Fresh tea leaves of all the 176 samples were harvested on the same day and extracted using 70% methanol with three biological replicates for each accession. All samples were analyzed by HPLC (Agilent, 1260 infinity) equipped with an Agilent TC-C18 reverse-phase column (5 μm, 250 mm × 4.6 mm) at 35 °C. A binary gradient elution system was adopted, and the mobile phase consisted of 0.1% formic acid-water (A) and 0.1% formic acid-methanol (B). The flow rate was maintained at 0.8 mL/min, and the detection wavelength was set to 278 nm. Each target component was identified by comparing the retention time to authentic standards. The standard curves used for quantification was calculated by using the linear regression equation of each component. Eight catechins (C, EC, CG, ECG, GC, EGC, GCG, and EGCG) and GA in each sample were quantified.

Phenotypic data were firstly checked by PCA plot in R^63^. The obvious outlier samples were manually removed before GWAS analysis. Missing genotypes were imputed by beagle 5.0^67^ with default parameters. GWAS was performed by EMMAX software^68^ and using BN matrix to control the population structure. The significant threshold of lead SNPs was determined by GEC software^69^ (1/independent markers). Variants with *P* value < 0.001 and within 500 kb to the lead SNPs and pairwise LD (*r*^2^) > 0.1 were merged into initial QTL region and each merged QTL region should at least harbor two or more SNPs. The final QTL region was extended by 100 kb based on the initial region. Candidate genes were selected in a candidate QTL region based on gene functional annotation and the correlation between phenotypic data and gene expression levels.

### Reporting summary

Further information on research design is available in the Nature Research Reporting Summary linked to this article.

## Supplementary information

Supplementary Information

Peer Review

Reporting Summary

Description of Additional Supplementary Files

Supplementary Data 1–18

## Data Availability

The data supporting the findings of this work are available within the paper and its Supplementary Information files. A reporting summary for this Article is available as a Supplementary Information file. The datasets generated and analyzed during the current study are available from the corresponding author upon request. All genome and transcriptome sequencing raw data described in this article are publicly available in the NCBI database under project PRJNA595851 for DASZ PacBio and Illumina reads, PRJNA595795 for RNA-Seq of 217 tea plant accessions, and PRJNA595898 for Illumina reads of Fudingdabai. DASZ genome assembly and annotation files are deposited to figshare database. Metabolic data are deposited to figshare database. Source data are provided with this paper.

## Source data

Source Data
