# Supplementary material for: Genome assembly of wild tea tree DASZ reveals pedigree and selection history of tea varieties
Source: Nat Commun. 2020 Jul 24;11:3719. doi: 10.1038/s41467-020-17498-6 (PMC7381669; doi:10.1038/s41467-020-17498-6)
Supplement: Supplementary file 1 — Supplementary Information [file 41467_2020_17498_MOESM1_ESM.docx]

**Genome assembly of wild tea tree DASZ reveals pedigree and selection history of tea varieties**

Zhang *et al.*

**Supplementary Method 1. Genome sequencing and assembly**

Plant material ­

The tea plant (*Camellia sinensis*) is one member of the section *Thea* of the genus *Camellia* in the family Theaceae. A wild tea landrace (named as DASZ; Supplementary Figure 1) found in Yunnan province, China, was sampled for whole genome sequencing and *de novo* assembly. Branches were harvested and transplanted in a greenhouse in Huazhong Agricultural University, Wuhan, China. Ten types of tissues (i.e., bud, first leaf, second leaf, mature leaf, stem, vein of young leaves, vein of mature leaves, stamens, roots, and petals) were harvested for transcriptome sequencing.

**Construction and sequencing of Illumina libraries**

High quality genomic DNA extracted from the young leaves was used for Illumina HiSeq libraries construction using the TruSeq Stranded DNA-seq kit (Illumina Biosystems, California, USA). The libraries were sequenced by the Illumina HiSeq system (paired-end 150-bp sequencing strategy). 244 Gbp raw data were generated from sequencing. After trimming the adapters and low-quality bases (minimum quality of 20) and the polluted reads by Trimmomatic^[1](#_ENREF_1)^ (v0.32) with default parameters, about 200 Gbp clean data were obtained and used for genome survey and correction of genome assembly.

Estimation of the genome size of DASZ

5 mm^2^ young leaves were shredded, incubated for 30 seconds and filtered, which were subsequently analyzed using a flow cytometer (Sysmex Partec GmbH, Germany) to estimate genome size and ploidy^[2](#_ENREF_2)^. *Maize* (*Zea mays L.* B73) was used as the genome size standard. The result of flow cytometer analysis shows that the estimated average genome size is 2.92 Gbp and DASZ is a diploid. The genome size was also estimated by Jellyfish[^3^](#_ENREF_3) (v1.1.12) program with the parameters (k=19, 21, 23) using 200 Gbp clean data. The estimated genome size is 3.8 Gbp with heterozygosity of 0.4% to 0.8% based on k-mer distribution of heterozygous sequences (Supplementary Figure 2).

**Construction and sequencing of SMRT PacBio libraries**

Five 20K *de novo* libraries, used for SMRT PacBio genome sequencing, were constructed according to standard manufacturers’ protocol (SMRT PacBio). The final libraries were sequenced on the PacBio Sequel platform (Pacific Biosciences). Totally, 39,763,171 reads were generated from 54 cells of PacBio data with a total length of 361 Gbp and N50 length of 14,084 bp (Supplementary Figure 3). All of these subreads were used for genome assembly.

*De novo* assembly of PacBio SMRT reads

A total of 39,763,171 PacBio SMRT subreads were used for contig assembly with Falcon[^4^](#_ENREF_4) (v0.3.0). The parameters of Falcon program were listed as follows (i.e., pa_DBsplit_option:-x2000 -s500; pa_HPCdaligner_option: -v -D24 -M30 -h300 -e.75 -w8 -l2000 -s1000 -k18; ovlp_HPCdaligner_option: -v -D24 -M30 -h1200 -e.96 -l1500 -s1000 -k25). HaploMerger2[^5^](#_ENREF_5) (v20180603) program (default parameters) was used for reducing redundancy and Arrow program (https://github.com/PacificBiosciences/SMRT-Link) with default parameters used for correcting the sequencing errors according to the alignments.

Illumina paired-end clean reads were mapped to the corrected above-mentioned contigs with BWA mem^[6](#_ENREF_6)^ (v0.7.16a) with default parameters, and high-quality mapped reads (MAQ >20) were further used to polish the assembly with Pilon^[7](#_ENREF_7)^ (v1.22) with default parameters. This procedure resulted in a total assembly length of 3.11 Gbp with an N50 length of 2.59 Mbp.

The evaluation of genome GC content

Illumina paired-end clean reads, mapped to assembly genome by SOAP aligner[^8^](#_ENREF_8) (v2.21) program, were used for evaluation of GC content and sequencing depth of genome. The results showed that the GC distribution of the genome of this sample concentrated at about 40%, and the depth concentrated at about 40X. The tendency of concentration in other GC regions at discrete points indicated that there is no contamination in the material (Supplementary Figure 4).

Analysis of sequencing depth

Base depth analysis was conducted to evaluate the coverage depth of single base and reflect the accuracy of base. Illumina paired-end clean reads were mapped to the assembled genome using BWA[^6^](#_ENREF_6) (v0.7.16a) program. The distribution of sequencing depth could be constructed by counting the coverage of each base of genome. The results showed that the majority of genome was 40X covered in average. And only 0.45% of genome was covered less than 10X (Supplementary Figure. 5).

**Chromosome conformation capture sequencing**

# About 3g young leaves were harvested after two days shading treatment. After cutting the plant leaves into fragments of about 4 mm^2^, we added 50 mL 2% formaldehyde into a container with sample fragments. The mixture was placed at room temperature for 15 min with intermittent agitation. Following procedures are: add 2.5 ml 2M glycine, and place it at room temperature for 5 min, and place it on ice for at least 15min to terminate the crosslinking; filter the cross-linked plant tissue solution with two layers of gauze, discard the solution and leave solid plant tissue; freeze the tissue sample after cross-linking by liquid nitrogen and stored in -80℃ for libraries construction. Nuclei extraction, nuclei permeabilization, chromatin digestion and proximity-ligation treatments were performed as previously described[^9^](#_ENREF_9). The recovery of Hi-C DNA and subsequent DNA manipulations were performed as previously described[^10^](#_ENREF_10). Two libraries with insertion sizes of 300 bp were sequenced on the Hiseq Xten instrument and generated 561.9 Gbp raw data. After data filtering, 390.2 Gbp high quality clean data were retained.

Hybrid assembly of PacBio contigs and Hi-C

The clean reads obtained from sequenced Hi-C libraries were mapped to draft contigs genome for obtaining global mapped reads using bowtie2[^11^](#_ENREF_11) (v2.2.5) and the parameters are as follows (BOWTIE2_GLOBAL_OPTIONS = --very-sensitive -L 30 --score-min L, -0.6, -0.2 --end-to-end –reorder, BOWTIE2_LOCAL_OPTIONS = --very-sensitive -L 20 --score-min L,-0.6,-0.2 --end-to-end --reorder). More than 49% of each single end reads are global mapped reads, and 39.76% raw reads were unique mapped read pairs (Supplementary Table 1, 2). And then unique mapped read pairs were mapped to the assembled genome for obtaining valid interaction pairs under HiC-Pro[^12^](#_ENREF_12) pipeline (v2.5.0) and 51.62% of them were obtained as valid interaction pairs (Supplementary Table 3). 45.12% unique valid interaction pairs were obtained after removing the duplicated pairs (Supplementary Table 4). Finally, we obtained (number of valid read pairs 1,300,782,477 * 39.76% * 51.62% * 45.12% = 120,447,024 read pairs) 36.1 Gpb effective bases for further analysis. Unique valid interaction pairs from each library were merged for interaction-matrix construction. Juicer[^13^](#_ENREF_13) (v1.0) and 3d-dna[^14^](#_ENREF_14) (v2.0) were applied to group, sort and target the contigs of draft genome, and finally evaluate the assembly results. The genome sequence was divided into bins of equal length of 100 Kbp fragment, and the number of unique valid interaction was determined between each pair of reported bins. A heatmap of Hi-C chromosomal interaction was created using HiC-pro[^12^](#_ENREF_12) software (v2.5.0) (Supplementary Figure 6). There were 5,453 PacBio contigs linked by Hi-C yielded a genome length of 3.11 Gbp in 1,237 scaffolds with an N50 of 204.21 Mb. 99.55% of whole genome sequence were anchored on 15 chromosomes.

Evaluation of genome assembly

To investigate the quality, accuracy and completeness of the genome assembly, three methods were used, including EST/Unigenes evaluation, BUSCOs data sets from the plant lineage and two genetic maps of tea plant[^15^](#_ENREF_15)^,^[^16^](#_ENREF_16). Benchmarking Universal Single-Copy Orthologs (BUSCO v3.0) was used for evaluation of the contigs genome assembly by against contigs to embryophyta_odb10 database[^17^](#_ENREF_17), the complete BUSCOs is 95.0% (Supplementary Table 5). Assessment of gene region coverage was conducted by querying public EST (Expressed Sequence Tag) data and the Unigenes in genome assembly using BLAT[^18^](#_ENREF_18) (v0.36). The result showed that EST and Unigenes assessment cover 90% and 97% of gene region, respectively. Genome assembly assessment using two genetic maps of tea plant was conducted by performing BLAT[^18^](#_ENREF_18) (v0.36) and ALLMAPS[^19^](#_ENREF_19) (v180626). Both of the coefficients of consistency between the current assembly and two published genetic maps of tea plant were more than 0.95 in average (Supplementary Table 6).

Genome annotation

The genome annotation mainly included repeat annotation, gene annotation and non-coding RNA annotation. The detailed pipeline is shown as the Supplementary Figure 7.

Repetitive sequences annotation

Repetitive families in the genome were identified using an integration of independent homology searching and *de novo* predictions. Of the genome, 87.41% was found to be repetitive, including a large proportion of long terminal repeat (LTR) transposable elements (Supplementary Table 7). These transposable elements include DNA, LINE, SINE and LTR (Supplementary Table 8, 9). The distribution of sequence divergence among four types of TEs is shown in Supplementary Figure 8 (Repbase homology shown in Supplementary Figure 8 left, *de novo* prediction shown in Supplementary Figure 8 right).

**Gene annotation**

To aid in gene annotation, RNA-seq was conducted on 10 different tissues from ‘DASZ’ (i.e., bud, first leaf, second leaf, mature leaf, stem, vein of young leaves, vein of mature leaves, stamens, roots, and petals). Total RNA of each sample was extracted with TRIzol according to the manufacturer’s instructions. RNA-seq libraries were prepared using the Illumina standard mRNA-seq library preparation kit and sequenced on the illumina HiSeq platform with 150 bp paired-end sequencing strategy and obtained 149.13 Gbp clean data. Two PacBio ISO-Seq libraries were constructed for a sample pooled from the above-mentioned 10 tissues and subsequently sequenced on the PacBio Sequel platform (Pacific Biosciences). 22.95 Gbp PacBio sequencing data was generated.

Gene model prediction

Strategy to predict non-redundant protein-encoding gene models was a combination of *de novo* prediction, homolog based prediction and EST/transcripts based prediction. For homolog prediction, Protein sequences from *Arabidopsis thaliana*[^20^](#_ENREF_20)*, Actinidia chinensis*^[21](#_ENREF_21)^*, Coffea canephora*^[22](#_ENREF_22)^*, Populus trichocarpa*^[23](#_ENREF_23)^*, Solanum lycopersicum*^[24](#_ENREF_24)^ and *Vitis vinifera*^[25](#_ENREF_25)^ were used to predict gene models using Exonerate[^26^](#_ENREF_26) (v2.2.0) and Genewise^[27](#_ENREF_27)^ (v2.4.1). For EST/transcripts-based prediction, RNA-Seq reads from different tissues were mapped to reference genome using hisat^[28](#_ENREF_28)^ (v2-2.1.0) and mapping results were assembled using StringTie^[29](#_ENREF_29)^ (v1.3.4d). Full-length transcripts generated from Iso-Seq were mapped onto the reference genome using Gmap^[30](#_ENREF_30)^ (v2014-08-04) and assembled using Pasa^[31](#_ENREF_31)^ (v2.3.3). EST evidence was also used in the prediction process. For de novo prediction, we first trained parameters based on 200 best predicted gene models in transcripts-based prediction. Then, Augustus[^32^](#_ENREF_32) (v3.3.1) and SNAP[^33^](#_ENREF_33) (v2006-07-28) were used for de novo prediction with the self-trained parameters. Finally, evidence of *de novo* prediction, homolog based prediction and EST/transcripts prediction was submitted to MAKER[^34^](#_ENREF_34) (v2.31.10) and 33,021 non-redundant genes were identified. Detailed information is shown in Supplementary Table 10 and Supplementary Figure 9.

Functional annotation

To assign gene functions to the protein-encoding gene models, we compared them to the databases SwissProt^[35](#_ENREF_35)^ (201709), TrEMBL^[35](#_ENREF_35)^, NR (20170924), KOG[^36^](#_ENREF_36) (20090331), KEGG[^37^](#_ENREF_37) (v84), Interpro^[38](#_ENREF_38)^ (v5.16-55.0), GO[^39^](#_ENREF_39) using Blast[^40^](#_ENREF_40) (v2.2.3). 30,511 of the total gene models had significant similarities in functional protein databases (Supplementary Table 11), and 20,337 were annotated in all the five databases (NR, InterPro, KEGG, SwissProt and KOG) (Supplementary Figure 10).

**Annotation of Non-coding RNA genes**

Non-coding RNA includes rRNA, tRNA, snRNA, miRNA, etc. We used tRNAscan-SE[^41^](#_ENREF_41) (v1.3.1) to identify tRNA genes. The rRNA genes were identified by searching the genome assembly against the rRNA sequences of closely related species using BLASTN[^40^](#_ENREF_40) (v2.2.3). miRNAs and snRNAs were predicted using INFERNAL of Rfam^[42](#_ENREF_42)^ (v12.0). 660 tRNA, 1870 rRNA, 151 miRNA and 568 snRNA were annotated in the whole genome (Supplementary Table 12).

Evaluation of gene set

Benchmarking Universal Single-Copy Orthologs^[17](#_ENREF_17)^ (BUSCOs v3.0) was used for evaluation of the completeness of gene set. 1375 conserved plant genes (embryophyta_odb10 database) were used and the complete BUSCO is 93.2% (Supplementary Table 13).

**Supplementary Method 2. Genomic variants and bioinformatics analysis**

**SV calling**

We remapped Pacbio reads of DASZ and Shuchazao to DASZ reference genome by ngmlr^[43](#_ENREF_43)^ (v0.2.7) with default parameters. Shuchazao Pacbio reads were downloaded from NCBI SRA database (SRR8334869). Bam files were sorted by samtools and the raw SV calling was done by sniffles 1.0.10 (Parameters for DASZ: -s 10 -t 30 --cluster --cluster_support 5 -q 30 -l 50 --genotype --report_seq; parameters for Shuchazao: -s 5 -t 30 --cluster --cluster_support 5 -q 30 -l 50 --genotype --report_seq). We filtered the raw SVs by the following steps: (1) SVs with flag ‘UNDETERMINED’ and ‘IMPRECISE’ were removed. (2) The break points of SVs were 200 bp near base ‘N’ were filtered. Such SVs may span two or more contigs or scaffolds. (3) Multiple SVs within overlapped regions were merged and SVs with more supported reads than others were kept. (4) Deletions and Insertions with supporting read exceeding the threshold (DASZ: 100; Shuchazao: 30) were removed. Sequencing depth was calculated using samtools^[44](#_ENREF_44)^ depth command. These SVs might be resulted from duplications or translocation events. (5) Finally, we removed deletions which spanned heterozygous SVs and these SVs were not belonging to the same clusters identified by sniffles. For evaluating the heterozygous SVs and assembly quality of Shuchazao genome, we re-mapped Shuchazao Pacbio reads to Shuchazao genome and called the SVs using the same procedures described above.

**Gene annotation of genes affected by SVs**

We classified heterozygous SVs identified in DASZ into six classes using an in-house perl (https://www.perl.org/) script (SVs overlapped with 5’UTR, 3’UTR, intron, CDS, presence absence (PAV) and intergenic regions). PAVs and SVs overlapped with CDS region were considered as high impact SVs. Genes affected by those high impact heterozygous SVs only processing one allele instead of the usual two alleles. These genes were thus considered as hemizygous genes. We performed GO annotation using WeGO^[45](#_ENREF_45)^ website, and the results showed that the most hemizygous genes were involved in cellular and metabolic process. GO enrichment analysis was done by binGO package in Cytoscape[^46^](#_ENREF_46)^,^[^47^](#_ENREF_47) software, and the results revealed that hemizygous genes was enriched in the GO term of ‘regulation of cell death’ (Supplementary Figure 12). Classification of SVs identified between DASZ and Shuchazao and subsequent GO annotation were used the same procedure described above.

**Variants calling of genome re-sequencing data and SNP effect annotation**

Shuchazao and Yunkang 10 re-sequencing reads were downloaded from NCBI (SRR5252474 and SRR5416919, respectively). Fudingdabai and DASZ were sequenced in this study. Low quality reads and adaptors of these four data were removed by Trimmomatic-0.36[^1^](#_ENREF_1) with default parameters. Clean reads were mapped to DASZ reference genome using bwa mem^[6](#_ENREF_6)^. PCR duplicates were removed by picard (http://broadinstitute.github.io/picard) and raw SNPs calling and filtering were performed by GATK[^48^](#_ENREF_48). Raw SNPs were filtered by GATK VariationFiltration (QD < 2.0 || FS > 60.0 || MQ <40.0 || MQRankSum < -12.5 || ReadPosRankSum < -8.0). SNP effects of DASZ were predicted by snpeff^[49](#_ENREF_49)^.

**Imputation for missing genotypes**

We conducted beagle[^50^](#_ENREF_50) (v5.0) to impute missing data. For evaluating the imputation accuracy, we first randomly masked 25% SNPs for each site and performed beagle with default parameters. The imputation accuracy was estimated using the proportion of true sites after imputation. In order to improve imputation accuracy, only SNPs with missing rate <= 80% and imputation accuracy >= 0.9 were kept for further analysis.

**Supplementary Method 3. Catechin variation in tea population**

The pairwise Pearson correlation between different catechins was calculated by Hmisc package and *P*-values were further adjusted by Bonferroni correction. Heatmap of catechin correlation was plotted by pheatmap package[^51^](#_ENREF_51) in R. We used lmer function in lme4 package[^52^](#_ENREF_52) in R to conduct linear mixed effects analysis to investigate the relationships between catechin contents and four factors including leaf size, tree shape, accession groups and sub-populations. We fitted the mixed linear model four times to test these four factors, respectively. For each time, we entered one factor as fixed effect and others as random effects into the model. *P*-values were obtained by likelihood ratio tests of the full model with the effect in question against the reduced model without the effect in question using anova function in R (https://cloud.r-project.org).

**Supplementary Method 4. Evolutionary analysis of gene families**

We first identified orthogroups using Orthofinder^[53-55](#_ENREF_53)^, using MUSCLE for sequence alignment[^56^](#_ENREF_56) and FastTree for the inference of phylogenetic trees[^57^](#_ENREF_57). We included in the analysis the full proteome files (derived from primary transcripts only) derived from the three tea genomes sequenced so far (including the DASZ genome reported here) and from 12 additional species from both Eudicots and Monocots (A. chinensis, A. thaliana, C sinensis, C. arabica, C. canephora, M. truncatula, O. sativa, P. trichocarpa, S. lycopersicum, T. cacao, V. vinifera and Z. mays). The number of the genes in each orthogroup and the ultrametric species tree were then used as the input files for CAFE[^58^](#_ENREF_58)^,^[^59^](#_ENREF_59), where the rate of gene birth and loss (λ) was estimated iteratively to find the best value maximising the log likelihood of the data from all orthogroups. CAFE compares the copy number, for each gene family, against the predicted size of the orthogroups in the ancestral nodes of the phylogeny. We then calculated the intersections of the rapidly evolving gene families across the different branches of the phylogeny using UpsetR^[60](#_ENREF_60)^. The orthogroups rapidly evolving in DASZ only (and thus, not evolving in A. chinensis and in the other tea lineages) were then annotated using Interpro^[38](#_ENREF_38)^.

**Supplementary Method 5. Functional verification of candidate genes**

**cDNA cloning and vector construction**

Non-synonymous SNPs in each gene were predicted by ANNOVAR[^61^](#_ENREF_61) and the contribution of each SNP to the metabolic trait was tested by ANOVA. Only significant SNPs were kept for further analysis (*P* < 0.05). Accessions with genotypes of high and low level phenotypic contribution were selected to amplify the cDNA for each gene, respectively. The PCR products were subsequently introduced into T-vectors and sequenced to select the allele for each gene. Primers using for vector construction were listed in Supplementary Table 18.

The selected alleles of *CsANR*, *CsF3’5’H* and *CsMYB5* were synthesized from Twist Bioscience. These fragments were then subcloned into pDONR207 Donor vector (Thermo Fisher Scientific, Waltham, MA) by PCR-based Gateway BP cloning according the protocol[^62^](#_ENREF_62). The stop codon was removed to fuse a nano-GFP tag with the C-terminal of each gene. Expression vectors for transient expression and enzyme assay were constructed using the Gateway LR reaction with pK7FWG2[^63^](#_ENREF_63).

**Transient expression analysis in the tobacco leaves and LC-MS**

The above constructs were transformed into *Agrobacterium* *tumefaciens* strain AGL1 and transiently expressed in tobacco leaves according to the previous protocol [^64^](#_ENREF_64). Two days after inoculation, tobacco leaves were checked by confocal microscopy to confirm the expression level of each gene. The tobacco leaves were harvested and crushed into powders in liquid nitrogen and then extracted in 80% methanol for the LC-MS measurement.

**Protein expression and enzyme activity assay**

All the enzymes were purified from tobacco leaves by the affinity purification with nano-GFP tag[^63^](#_ENREF_63). The tea leaves were extracted using 80% Methanol and dried for the in vitro enzyme assay. Both CsANRa and CsANRb were incubated with tea extract and 0.1 mM of the co-factor NADPH for 30 min in an end stopped metabolite assay. Similarly, the CsF3’5’Ha and CsF3’5’Hb were incubated with tea extract and co-factor of 0.2mM NADPH/NADH for 30 min. The empty GFP protein was processed the same treatments of ANR or F3’5’H as a negative control. Kinetic constants for ANR were determined using a concentration gradient of cyanidin chloride (30, 40 and 60 μM; PHL80022 Supelco) with a total volume of 200 μl containing 100 mM Tris-HCl (pH 8.0), 12 mM NADPH and 30 μg purified cleaved ANR according the method by Xie et al[^65^](#_ENREF_65).


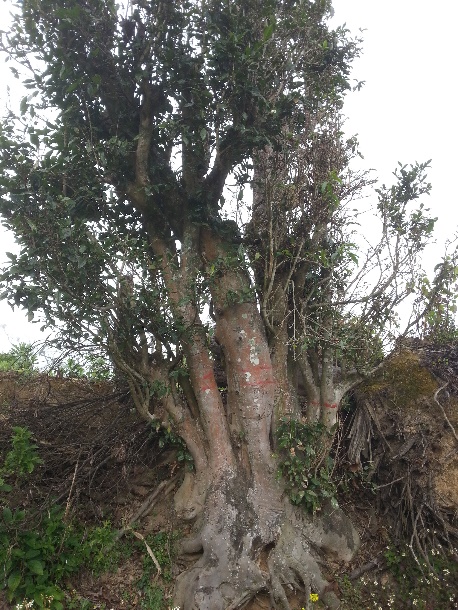


**Supplementary Figure 1. Tea plant DASZ**. The photo was taken by one of our co-authors Dr. Dejiang Ni.


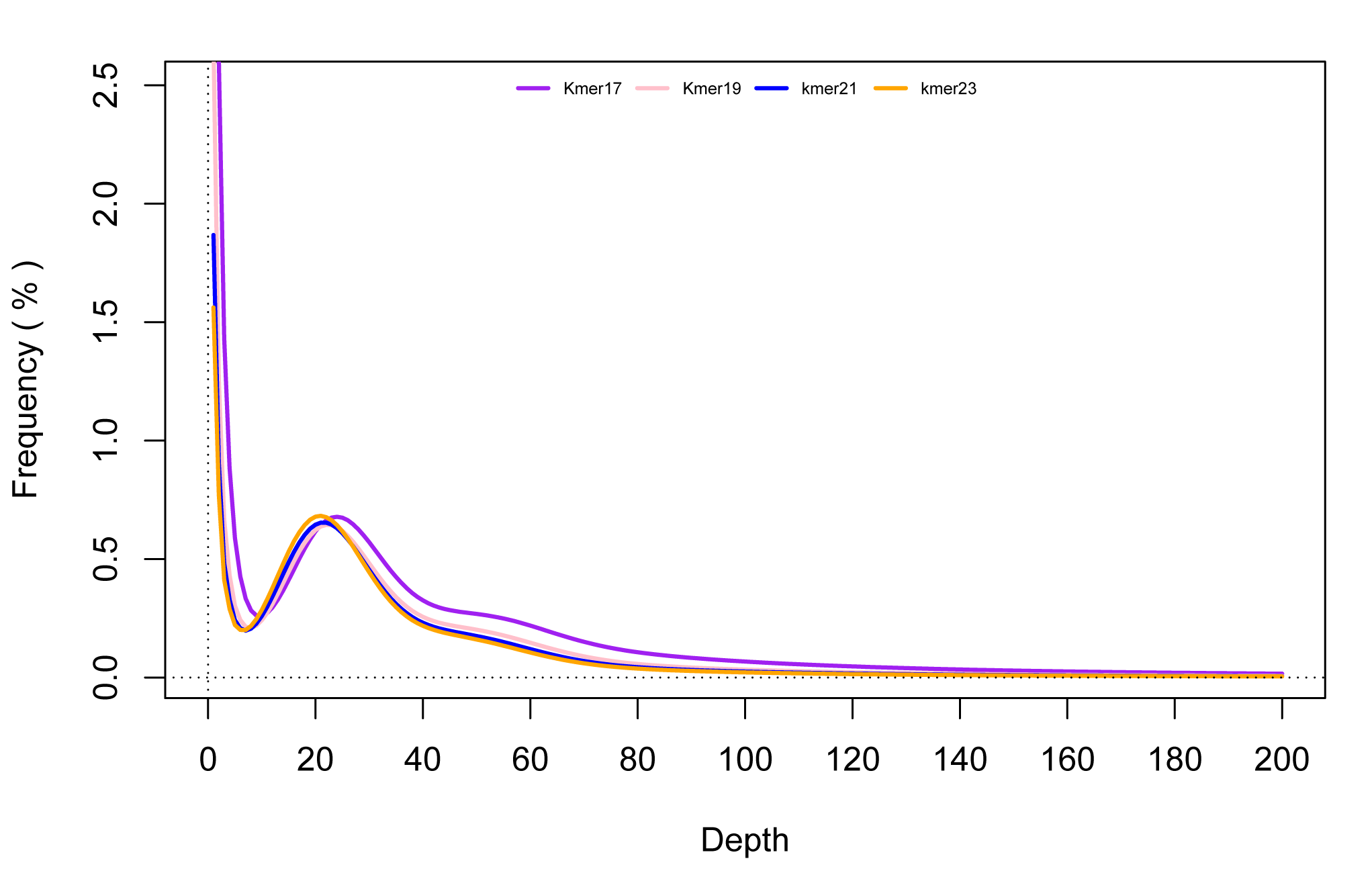


**Supplementary Figure 2. The distribution of K-mer. Source data are provided as a Source Data file.**

**Supplementary Figure 3. Distribution of length of PacBio sequencing subreads. Source data are provided as a Source Data file.**


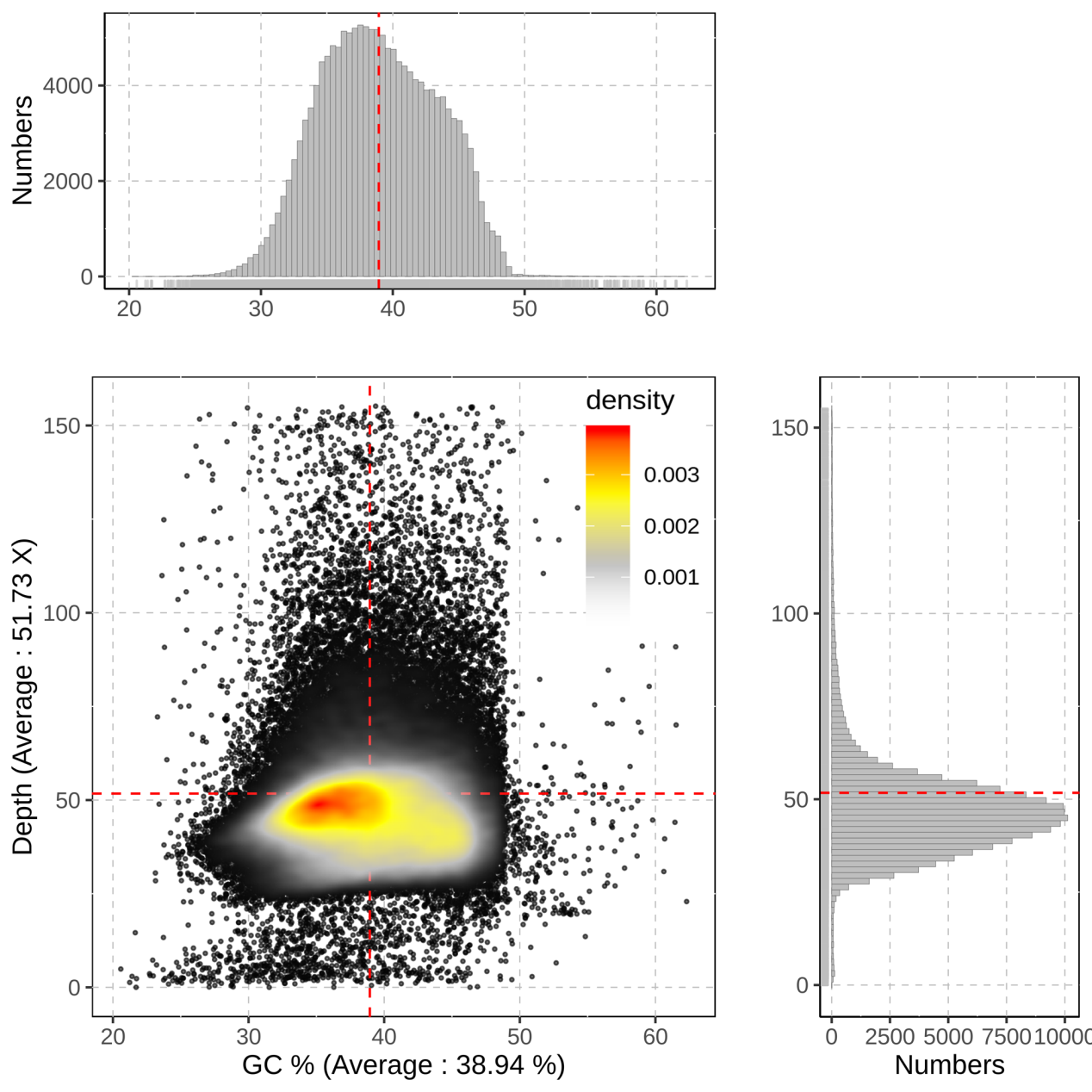


**Supplementary Figure 4. GC content and the distribution of average depth under a 20 Kbp non overlapped slide window of genome. Source data are provided as a Source Data file.**


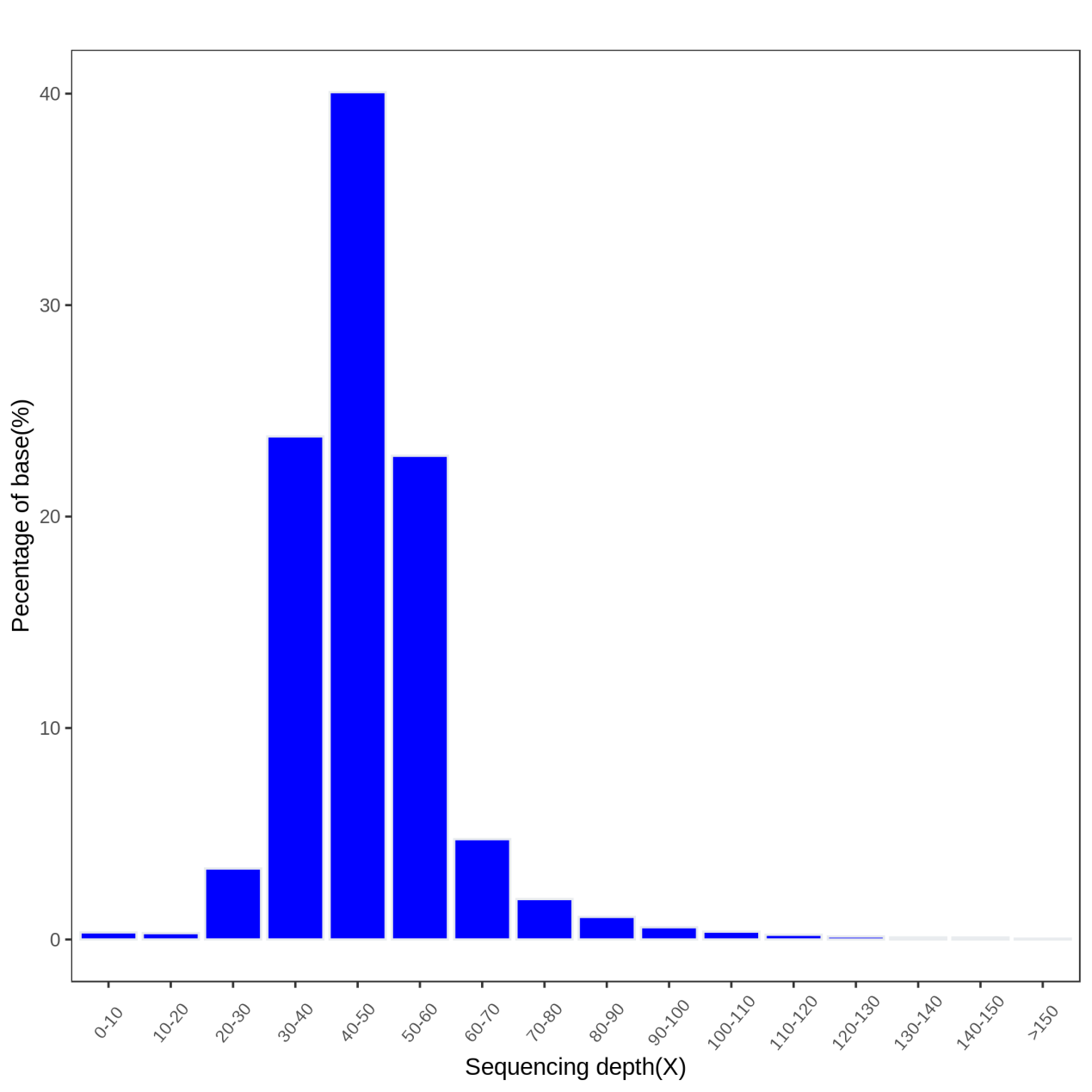


**Supplementary Figure 5. The distribution of sequencing depth. Source data are provided as a Source Data file.**

**
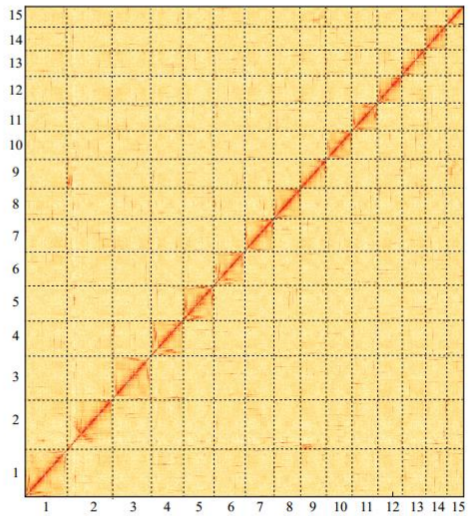
**

**Supplementary Figure 6. Heatmap of Hi-C chromosomal interaction.**

Hi-C interactions among 15 chromosomes with a 100-kb resolution. Dark red indicates strong interactions and yellow indicates weak interactions. **Source data are provided as a Source Data file.**


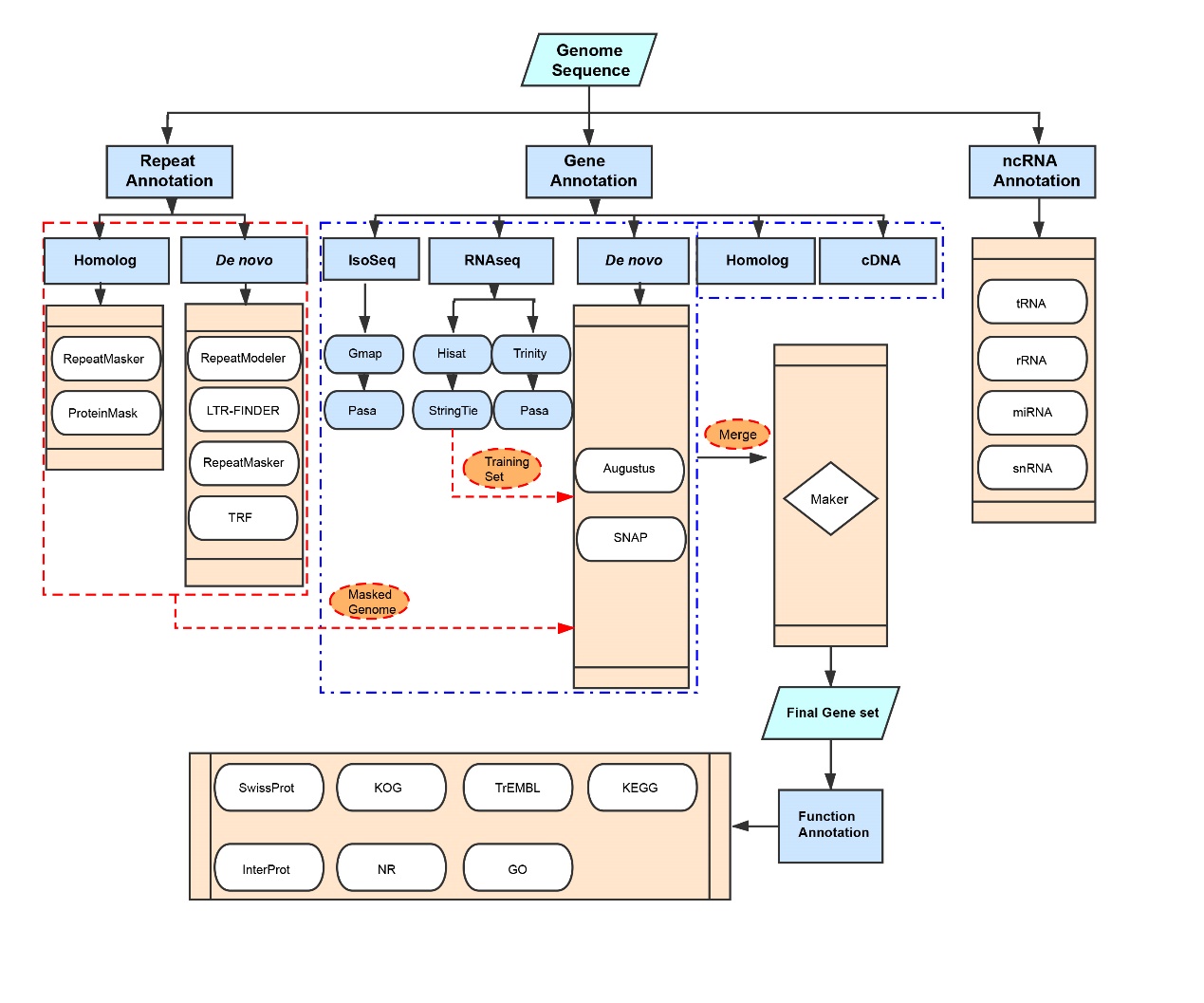


**Supplementary Figure 7. The pipeline of genome annotation in tea plant.**


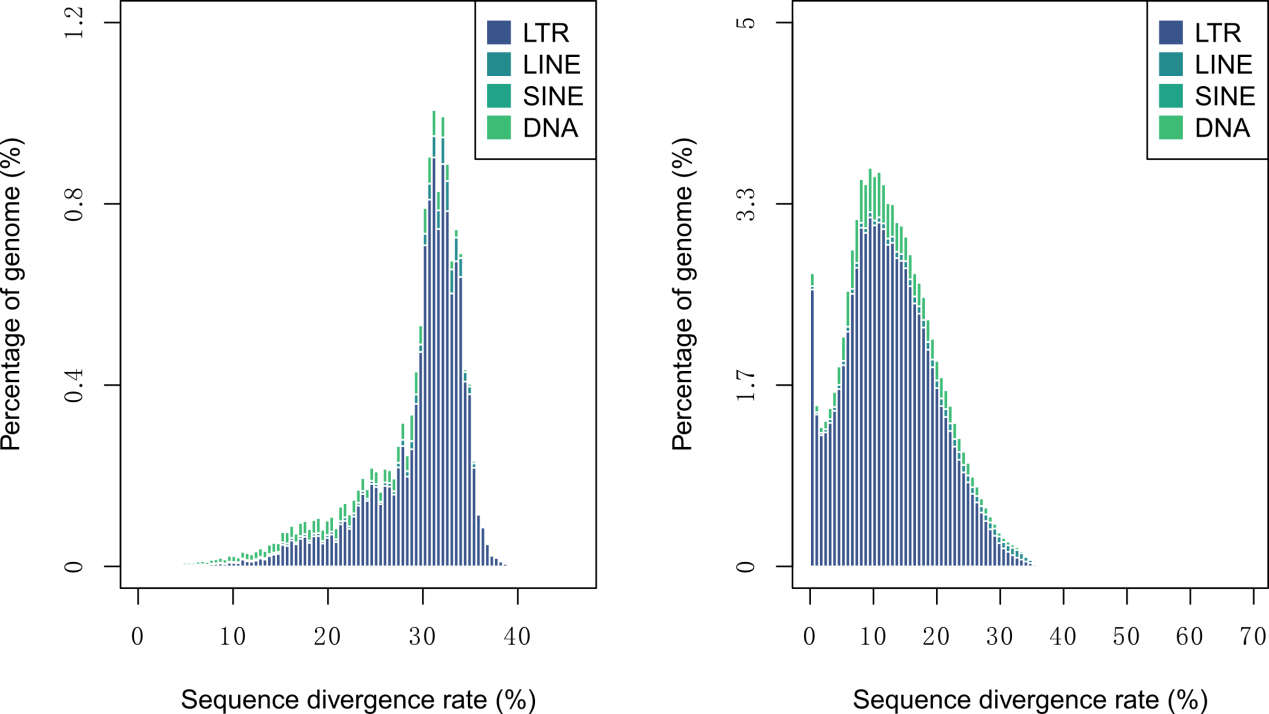


**Supplementary Figure 8. The distribution of sequence divergence among four types of TE.** The left panel shows the distribution of sequence divergence among four types of TE from Repbase homology. The right panel shows the distribution of sequence divergence among four types of TE from *de novo* prediction. **Source data are provided as a Source Data file.**


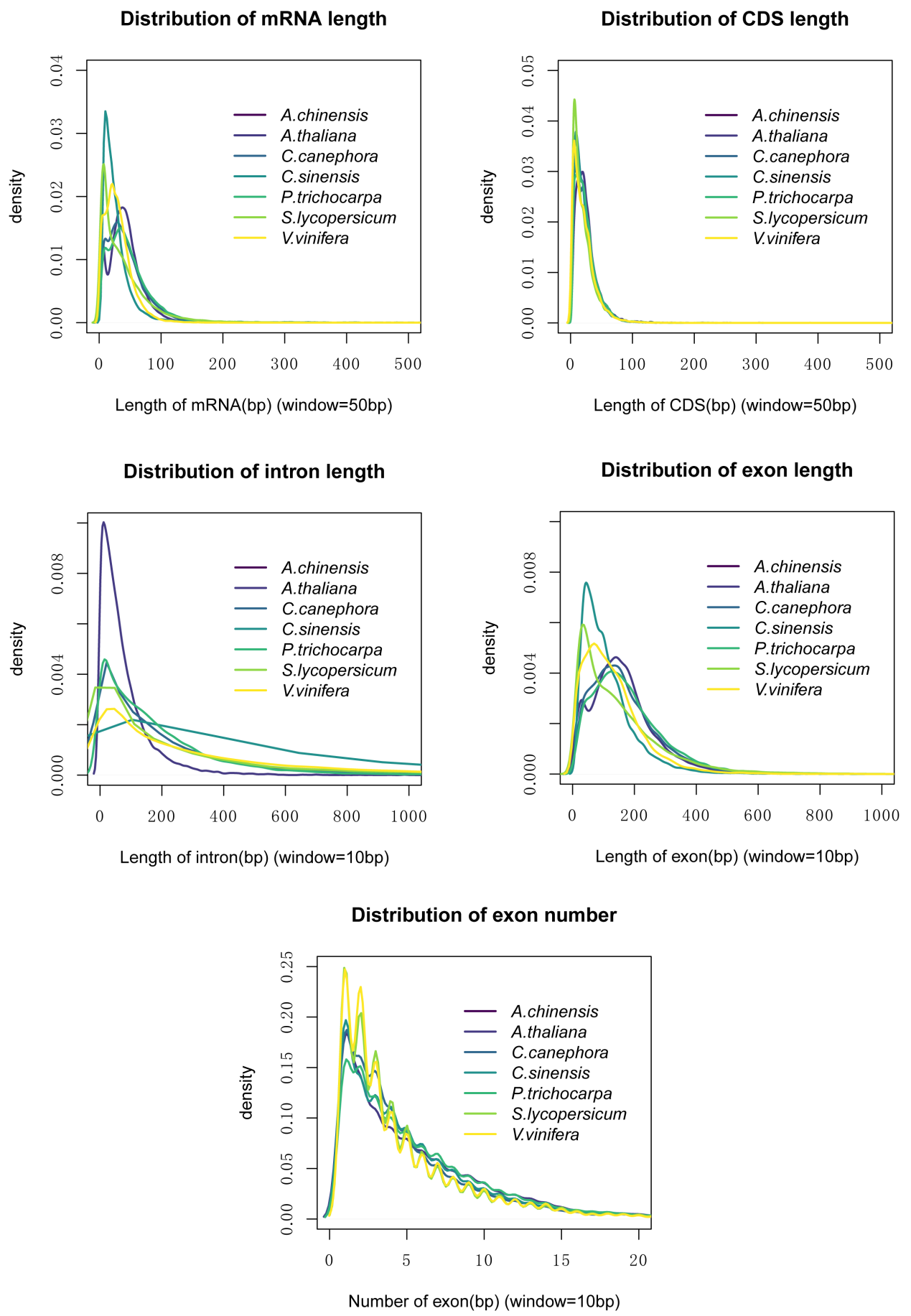


**Supplementary Figure 9. Statistics of gene model prediction**. Different colors represent different species. **Source data are provided as a Source Data file.**


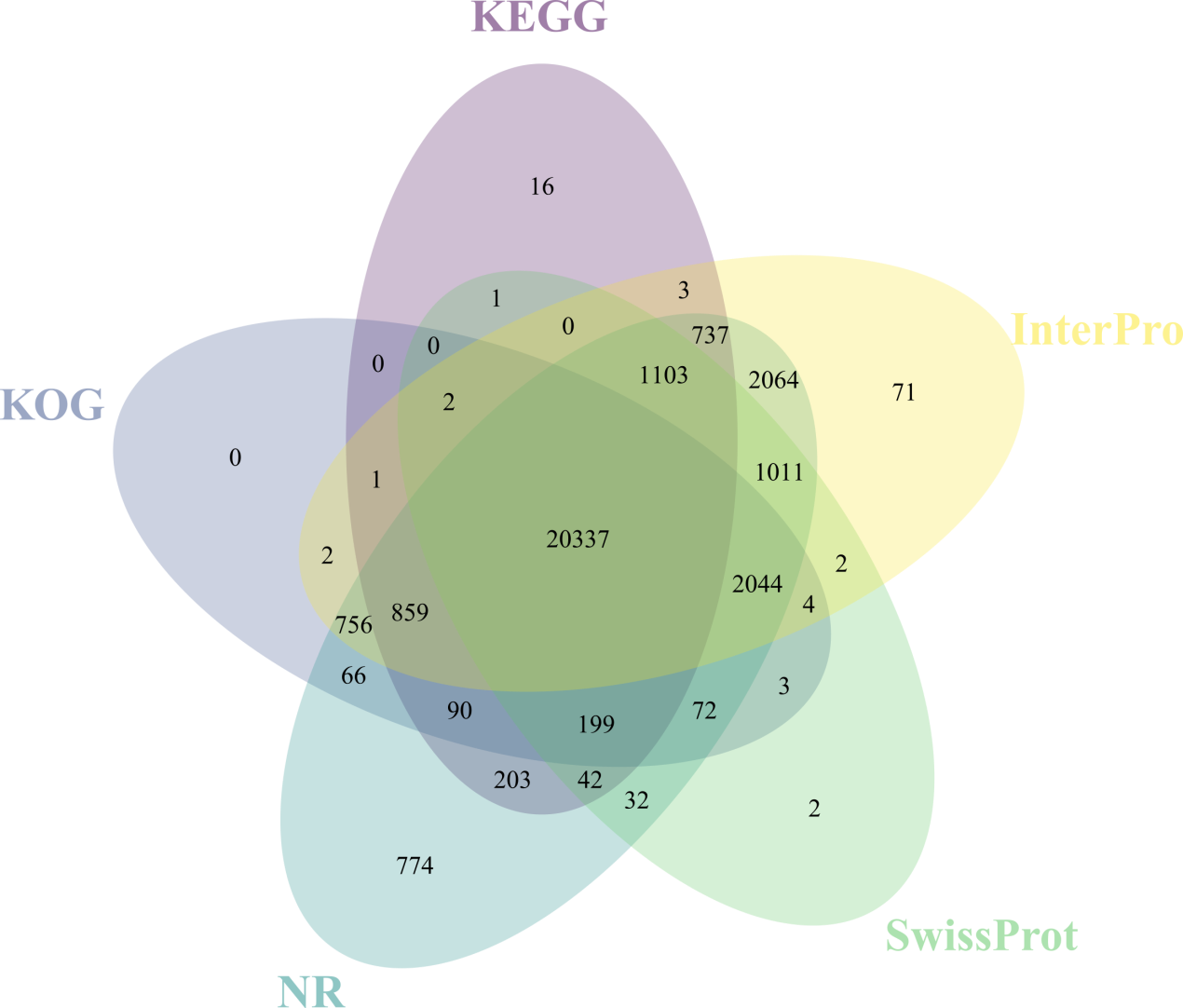


**Supplementary Figure 10. Venn plot of gene annotation**. Results are based on NR, InterPro, KEGG, SwissProt and KOG databases. **Source data are provided as a Source Data file.**


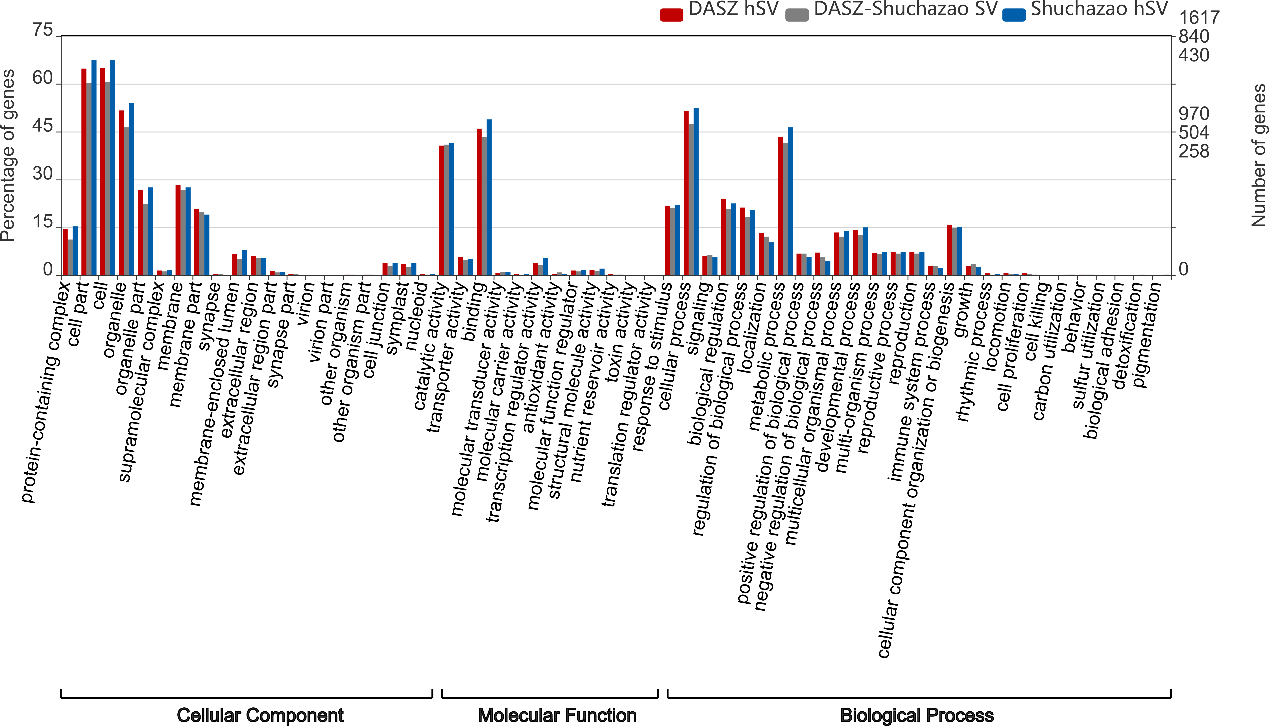


**Supplementary Figure 11. GO annotation of genes affected by SVs**. Different colors show the genes affected by DASZ hSVs, DASZ-Shuchazao SVs and Shuchazao hSVs, respectively. **Source data are provided as a Source Data file.**


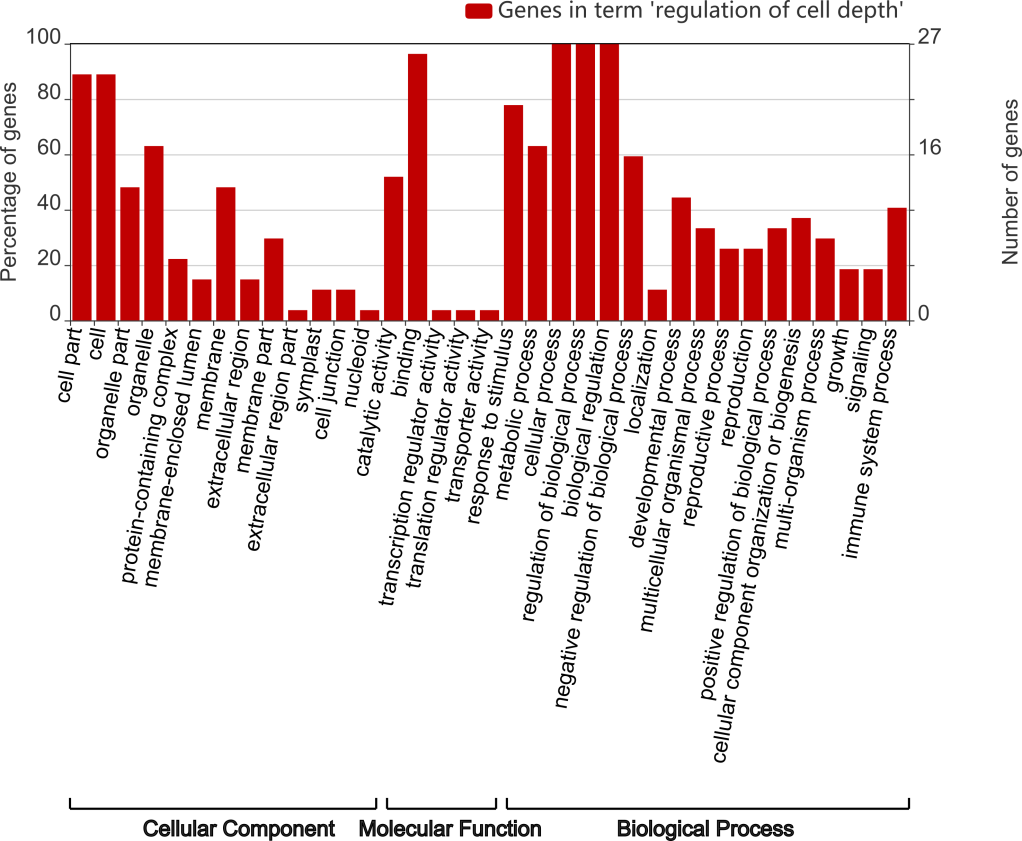


**Supplementary Figure 12. GO annotation of 27 genes affected by hSVs and related with GO term ‘regulation of cell depth’ in DASZ. Source data are provided as a Source Data file.**


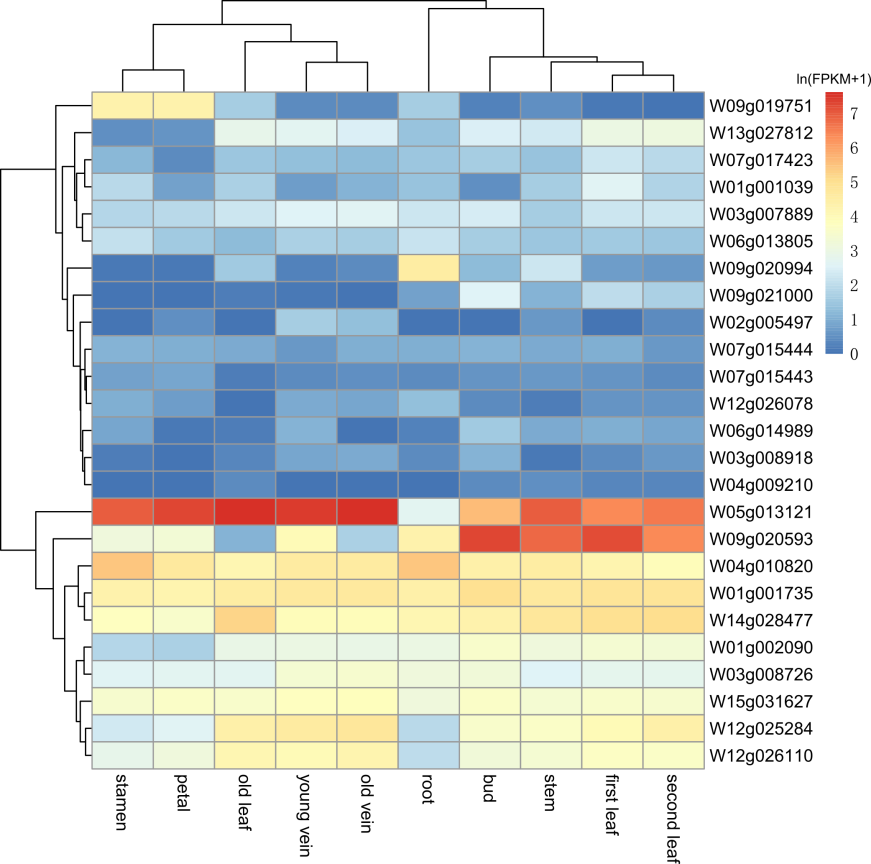


**Supplementary Figure 13. Heatmap of expression of genes affected by hSVs and related with GO term ‘regulation of cell depth’ across ten tissues of DASZ.** X-axis indicates 10 tissues of DASZ and Y-axis shows the gene IDs. Gene expression levels have been ln transformed and indicated by different colors shown in the right of the plot. **Source data are provided as a Source Data file.**


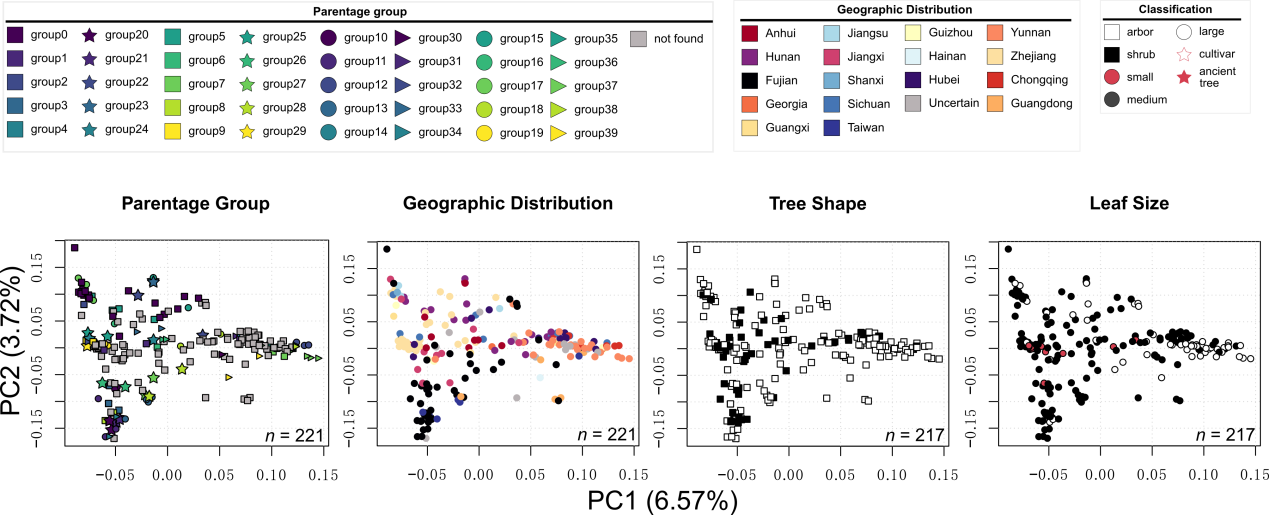


**Supplementary Figure 14. PCA plots of tea accessions.** The meanings of shapes and colors of each point are indicated by legend in the top. **Source data are provided as a Source Data file.**


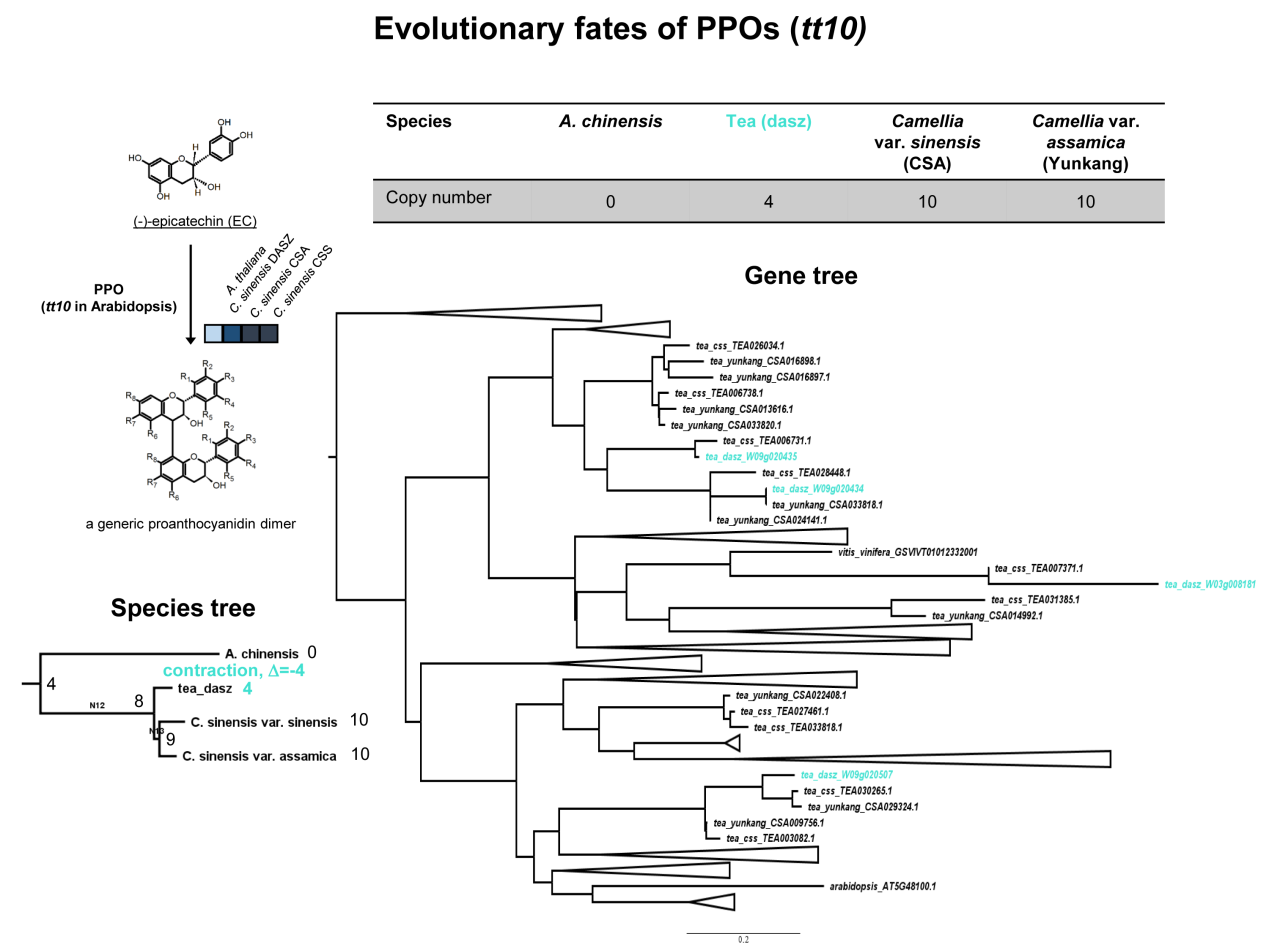


**Supplementary Figure 15. Evolutionary fates of PPOs.** Copy numbers of PPOs in each species are shown in the top table. Left part of the figure shows general scheme for the reaction of PPOs in *Camellia* spp. PPO could catalyze epicatechin (EC) into proanthocyanidin dimer. For the main metabolic steps, the number of genes contained in the respective orthogroup from *A. thaliana*, *C. sinensis* (DASZ), *C. sinensis* var. *assamica* (CSA) and *C. sinensis* var. *sinensis* (CSS) is indicated by the colored boxes. Right part of the figure indicates the phylogenetic tree of PPO genes. Turquoise node names indicate PPOs of DASZ. Lower left of the figure shows the species tree of A. chinensis, DASZ, Yunkang10 and Shuchazao which indicates the contraction of PPO gene family in DASZ.


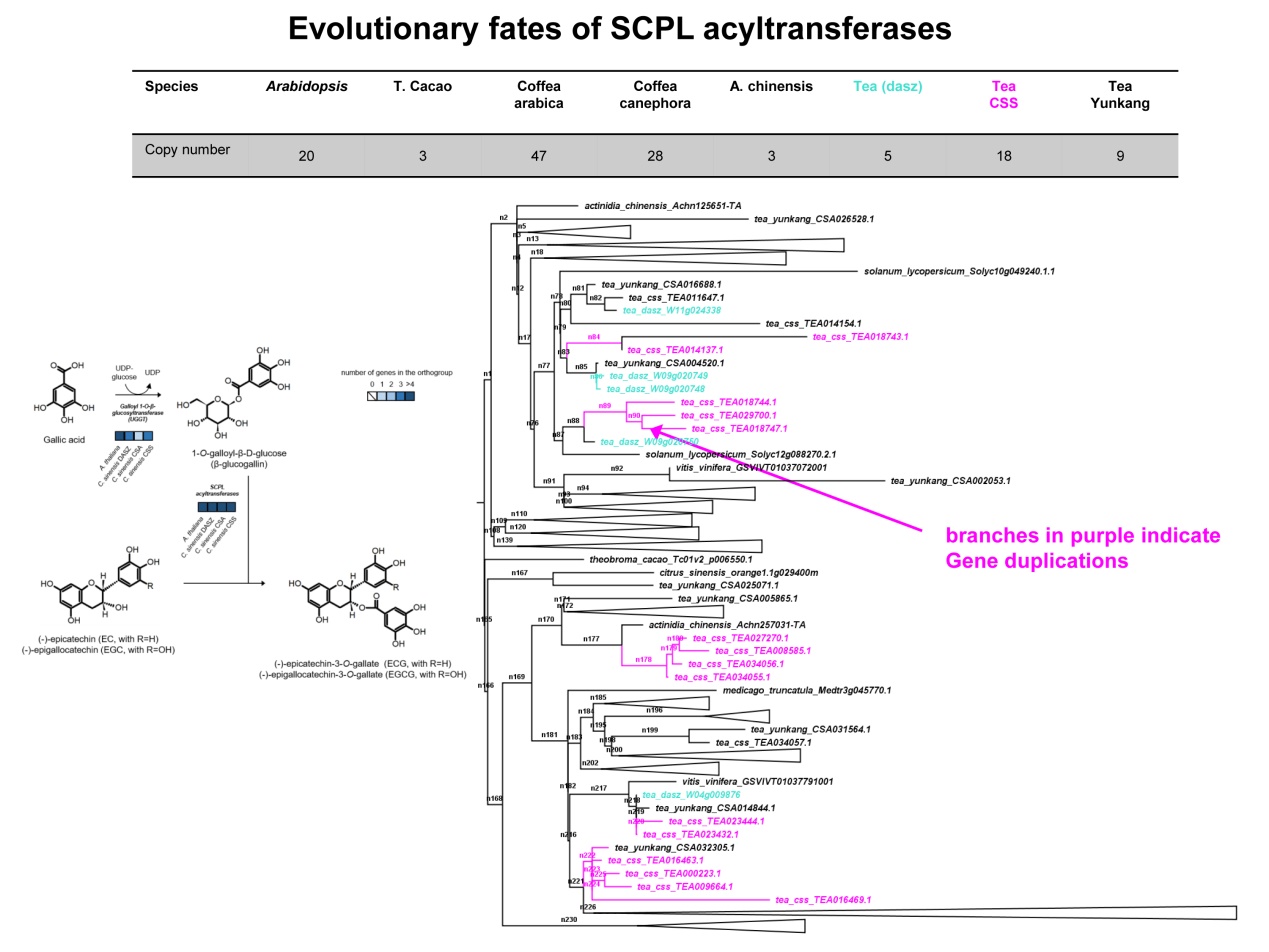


**Supplementary Figure 16. Evolutionary fates of SCPL acyltransferases.** Copy numbers of SCPL acyltransferases in each species are shown in the top table. Left part of the figure shows general scheme for the biosynthesis of galloylated catechins in *Camellia* spp. Synthesis of galloylated catechins starts with the glycosylation of gallic acid by the action of galloyl-1-O-β-D-glucosyltransferase. Galloylglucose is then esterified to a catechin molecule by the action of a Serine CarboxyPeptidase-Like (SCPL) acyltransferase. (-)-epigallocatechin-3-O-gallate usually represents the most abundant catechin in tea plant, reaching up to 75% of all catechins. For the main metabolic steps, the number of turquoise contained in the respective orthogroup from *A. thaliana*, *C. sinensis* (DASZ), *C. sinensis* var. *assamica* (CSA) and *C. sinensis* var. *sinensis* (CSS) is indicated by the colored boxes. Right part of the figure indicates the phylogenetic tree of SCPL acyltransferases genes. Purple node names represent SCPL acyltransferases of Shuchazao and turquoise node names indicate SCPL acyltransferases of DASZ. Branches in purple indicate gene duplication events.


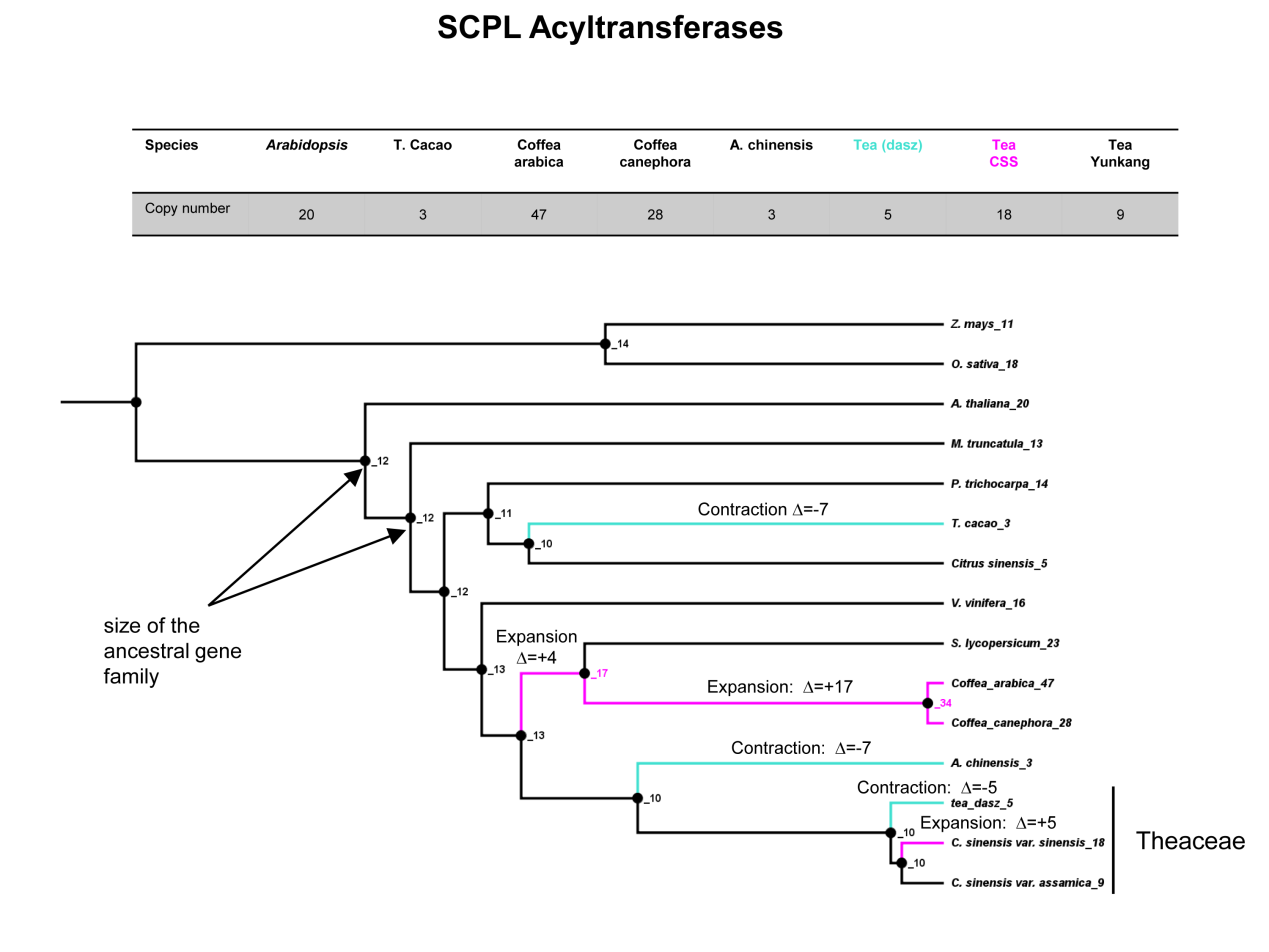


**Supplementary Figure 17. Expansion and contraction of SCPL acyltransferases genes.** Table in the top of the figure shows the copy numbers of SCPL acyltransferases in each species. Purple and turquoise branches indicate the expansion events and contraction events, respectively.


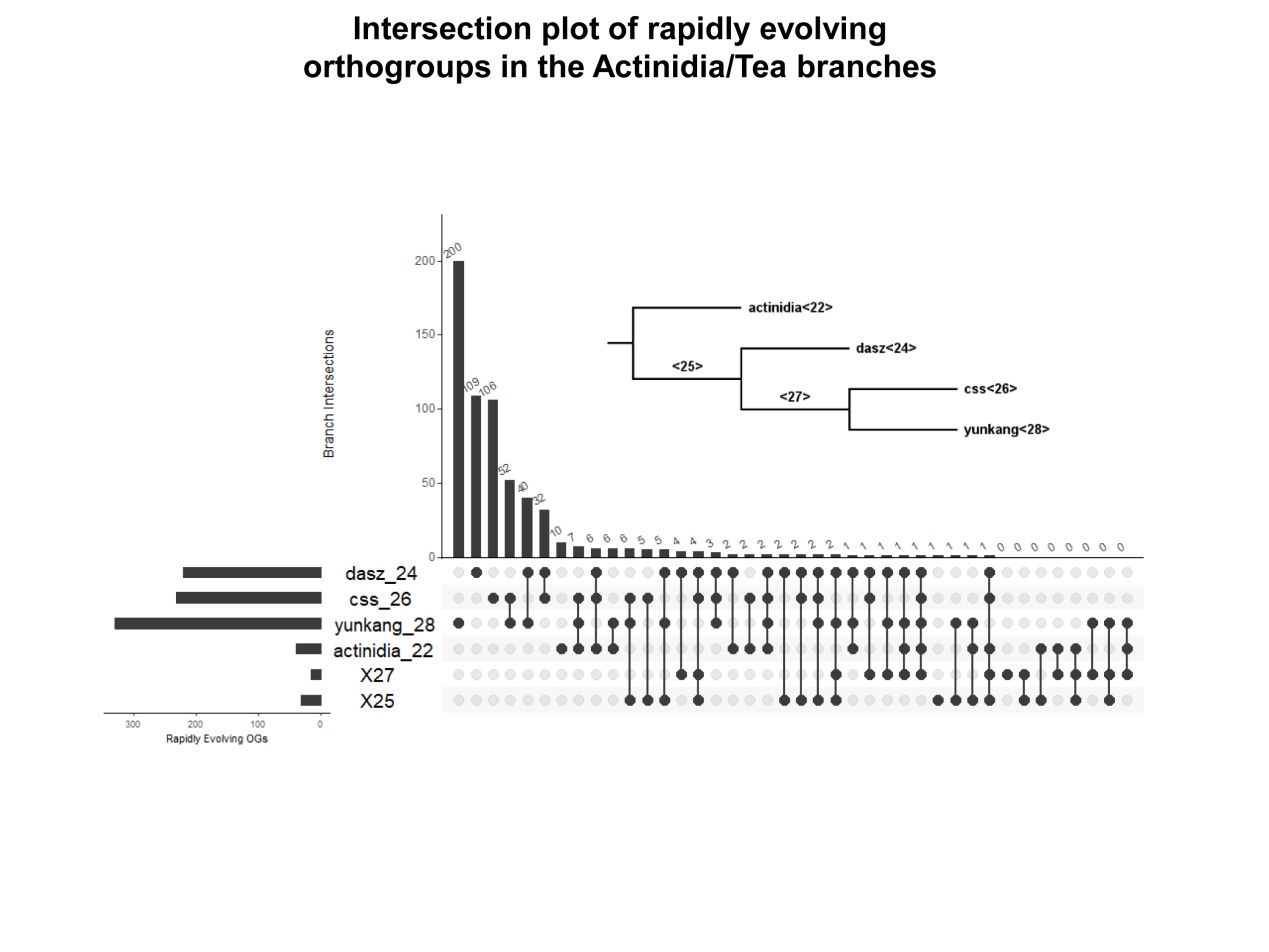


**Supplementary Figure 18. Intersection plot of the rapidly evolving orthogroups across the branches of the tea phylogeny.** The main histogram bars refer to the number of shared orthogroups from the branches/species indicated below in black circles and connected by lines. The first three columns in the histogram represent the number of unique orthogroups (OGs) (outside of the intersections of the six species/branches) which are rapidly evolving only in one of the three tea species. The histogram in the lower left of the figure shows the total number of rapidly evolving orthogroups in each species/branch.

| **Supplementary Table 1. Statistics of clean data from Hi-C mapped to genome.**   \| **Library** \| **Reads** \| \| **Mapping type** \| \| **Number** \| \| **Ratio（%）** \| \| \| --- \| --- \| --- \| --- \| --- \| --- \| --- \| --- \| --- \| \| CS_total \| R1 \| Total clean read pairs \| \| 1,300,782,477 \| \| 100 \| \| \| Mapped \| \| 1,087,235,182 \| \| 83.58 \| \| \| Global mapped to genome \| \| 686,205,087 \| \| 52.75 \| \| \| Local mapped to genome \| \| 401,030,095 \| \| 30.82 \| \| \| R2 \| Total clean read pairs \| \| 1,300,782,477 \| \| 100 \| \| \| Mapped \| \| 1,030,899,909 \| \| 79.25 \| \| \| Global mapped to genome \| \| 637,697,586 \| \| 49.02 \| \| \| Local mapped to genome \| \| 393,202,323 \| \| 30.22 \| \|     **Supplementary Table 2. Merged mapped read pairs.**   \| **Library** \| **Mapping type** \| **Ratio（%）** \| \| --- \| --- \| --- \| \| CS_total \| unique pairs \| 39.76 \| \| mutiple and singleton pairs \| 53.4 \| \| unmapping pairs \| 6.84 \|   **Supplementary Table 3. Valid and invalid read pairs.**   \| **Library** \| **Type** \| **Ratio (%)** \| \| --- \| --- \| --- \| \| CS_total \| Valid Interaction Pairs \| 51.62 \| \| Invalid Pairs \| 48.38 \|   **Supplementary Table 4. Statistic of valid interaction read pairs.**   \| **Library** \| **Type** \| **Ratio (%)** \| \| --- \| --- \| --- \| \| CS_total \| Valid Interaction Pairs \| 45.12% \| \| Duplicates \| 54.88% \|   **Supplementary Table 5. Summary of BUSCOs genome evaluation.**   \|  \|  \| \| \| --- \| --- \| --- \| \|  \| Number \| Percent (%) \| \| Complete and single-copy BUSCOs* (S) \| 1,209 \| 87.9 \| \| Complete and duplicated BUSCOs (D) \| 97 \| 7.1 \| \| Fragmented BUSCOs (F) \| 21 \| 1.5 \| \| Missing BUSCOs (M) \| 48 \| 3.5 \|   * The database was embryophyta_odb10 (Creation date:2017-12-01, number of species: 60, number of BUSCOs: 1375) |  |
| --- | --- | --- | --- | --- | --- | --- | --- | --- | --- | --- | --- | --- | --- | --- | --- | --- | --- | --- | --- | --- | --- | --- | --- | --- | --- | --- | --- | --- | --- | --- | --- | --- | --- | --- | --- | --- | --- | --- | --- | --- | --- | --- | --- | --- | --- | --- | --- | --- | --- | --- | --- | --- | --- | --- | --- | --- | --- | --- | --- | --- | --- | --- | --- | --- | --- | --- | --- | --- | --- | --- | --- | --- | --- | --- | --- | --- | --- | --- | --- | --- | --- | --- | --- | --- | --- | --- | --- | --- | --- | --- | --- | --- | --- | --- | --- | --- | --- | --- | --- | --- | --- | --- | --- | --- | --- |

| **Supplementary Table 6. The coefficient of consistency between the genome assembly and two published genetic maps of tea.**   \| **Chromosome** \| **Genetic map 1**[**^15^**](#_ENREF_15) \| **Genetic map 2**[**^16^**](#_ENREF_16) \| \| --- \| --- \| --- \| \| chr1 \| 0.824 \| 0.909 \| \| chr2 \| 0.988 \| 0.927 \| \| chr3 \| 0.96 \| 0.993 \| \| chr4 \| 0.958 \| 0.882 \| \| chr5 \| 0.895 \| 0.98 \| \| chr6 \| 0.883 \| 0.987 \| \| chr7 \| 0.974 \| 0.966 \| \| chr8 \| 0.988 \| 0.983 \| \| chr9 \| 0.971 \| 0.939 \| \| chr10 \| 0.977 \| 0.983 \| \| chr11 \| 0.971 \| 0.995 \| \| chr12 \| 0.975 \| 0.978 \| \| chr13 \| 0.974 \| 0.989 \| \| chr14 \| 0.987 \| 0.982 \| \| chr15 \| 0.947 \| 0.915 \|   **Supplementary Table 7. Summary of repetitive sequence identification.** | | | |
| --- | --- | --- | --- | --- | --- | --- | --- | --- | --- | --- | --- | --- | --- | --- | --- | --- | --- | --- | --- | --- | --- | --- | --- | --- | --- | --- | --- | --- | --- | --- | --- | --- | --- | --- | --- | --- | --- | --- | --- | --- | --- | --- | --- | --- | --- | --- | --- | --- | --- | --- | --- |
| **Type** | **Repeat Size(bp)** | **Percent of genome (%)** | |
| TRF | 242,936,417 | 7.8 | |
| RepeatMasker | 476,112,853 | 15.29 | |
| RepeatProteinMask | 555,348,235 | 17.84 | |
| *De novo* | 2,705,689,613 | 86.91 | |
| Total | 2,721,167,198 | 87.41 | |

**Supplementary Table 8. Summary of repeat classification.**

|  | **RepBase TEs** | | **TE Proteins** | | **De novo** | | **Combined TEs** | |
| --- | --- | --- | --- | --- | --- | --- | --- | --- |
|  | **Length (bp)** | **Percent of Genome (%)** | **Length (bp)** | **Percent of Genome (%)** | **Length(bp)** | **Percent of Genome (%)** | **Length (bp)** | **Percent of Genome (%)** |
| **DNA** | 51,689,457 | 1.660404 | 39,983,546 | 1.284378 | 294,994,556 | 9.476016 | 329,705,659 | 10.59103 |
| **LINE** | 24,610,566 | 0.790557 | 32,840,835 | 1.054935 | 91,608,406 | 2.942708 | 101,570,242 | 3.262709 |
| **SINE** | 395,750 | 0.012713 | 0 | 0 | 236,126 | 0.007585 | 397,798 | 0.012778 |
| **LTR** | 403,344,130 | 12.956495 | 485,040,715 | 15.580808 | 2,378,556,120 | 76.405603 | 2,384,429,337 | 76.594266 |
| **Other** | 267,05 | 0.000858 | 0 | 0 | 6,358 | 0.000204 | 27,266 | 0.000876 |
| **Unknown** | 0 | 0 | 0 | 0 | 95,116,538 | 3.055398 | 95,116,538 | 3.055398 |
| **Total** | 476,112,853 | 15.294022 | 555,348,235 | 17.839274 | 2,670,314,287 | 85.777657 | 2,674,861,882 | 85.923738 |

**Supplementary Table 9. Repetitive sequence categories and contents in DASZ.**

| **Type** | **Length (bp)** | **% in genome** |
| --- | --- | --- |
| DNA/CMC-EnSpm | 14,346,803 | 0.461 |
| DNA/MuLE-MuDR | 139,668,578 | 4.487 |
| DNA/PIF-Harbinger | 11,930,357 | 0.383 |
| DNA/hAT-Ac | 5,645,596 | 0.181 |
| DNA/hAT-Tag1 | 2,556,005 | 0.082 |
| DNA/hAT-Tip100 | 5,072,854 | 0.163 |
| other DNA | 7,231,169 | 0.232 |
| LINE | 72,459,424 | 2.328 |
| SINE | 233,642 | 0.008 |
| LTR/Caulimovirus | 21,364,138 | 0.686 |
| LTR/Copia | 264,411,050 | 8.494 |
| LTR/Gypsy | 1,536,720,337 | 49.364 |
| other LTR | 483,958,188 | 15.546 |
| RC/Helitron | 4,535,893 | 0.146 |
| Simple_repeat | 96,685,536 | 3.106 |
| Unknown | 54,340,748 | 1.746 |

**Supplementary Table 10. Gene model prediction.**

|  | **Gene set** | **Number** | **Average gene length (bp)** | **Average CDS length (bp)** | **Average exon per gene** | **Average exon length (bp)** | **Average intron length (bp)** |
| --- | --- | --- | --- | --- | --- | --- | --- |
| *De novo* | AUGSTUTUS | 44,491 | 5,962.03 | 1,204.41 | 4.44 | 271.26 | 1,382.98 |
|  | SNAP | 41,669 | 2,068.74 | 477.24 | 2.73 | 174.54 | 917.66 |
| Homolog | *A.thaliana* | 37,662 | 5,481.21 | 904.69 | 3.43 | 263.89 | 1,884.65 |
|  | *A.chinensis* | 91,902 | 4,029.72 | 738.3 | 2.57 | 287.84 | 2,103.14 |
|  | *C.canephora* | 73,662 | 5,052.80 | 750.23 | 2.7 | 277.67 | 2,528.13 |
|  | *P.trichocarpa* | 83,001 | 5,144.92 | 870.61 | 3 | 290.21 | 2,137.28 |
|  | *S.lycopersicum* | 62,088 | 5,154.61 | 854.8 | 3.05 | 280.55 | 2,100.66 |
|  | *V.vinifera* | 74,936 | 5,187.40 | 833.23 | 2.79 | 298.29 | 2,427.98 |
|  | Pacbio transcripts | 49,904 | 13,850.60 | 2,750.15 | 7.84 | 350.82 | 1,623.05 |
| Integration | Maker | 34,459 | 8,332.28 | 1,106.72 | 5.47 | 202.4 | 1,353.03 |
| Final gene set |  | 33,021 | 8,050.50 | 1139.09 | 5.38 | 211.68 | 1,330.34 |

**Supplementary Table 11. Summary of gene function annotation.**

|  | **Total** | **NR** | **Swissprot** | **KEGG** | **KOG** | **TrEMBL** | **Interpro** | **GO** | **Overall** |
| --- | --- | --- | --- | --- | --- | --- | --- | --- | --- |
| Number | 33,021 | 30,389 | 24,854 | 23,593 | 24,435 | 30,241 | 28,996 | 17,370 | 30,511 |
| Percentage | 100% | 92.03% | 75.27% | 71.45% | 74.00% | 91.58% | 87.81% | 52.60% | 92.40% |

Supplementary Table 12. Summary of Non-coding RNA gene annotation.

| **Type** | | **Copy (w)** | **Average length (bp)** | | **Total length (bp)** | **percent of genome** |
| --- | --- | --- | --- | --- | --- | --- |
| miRNA |  | 151 | 123.8477 | 18,701 | | 0.000601 |
| tRNA |  | 660 | 75.45909 | 49,803 | | 0.0016 |
| rRNA | rRNA | 1,870 | 184.7551 | 345,492 | | 0.011098 |
|  | 18S | 169 | 1,057.462 | 178,711 | | 0.005741 |
|  | 28S | 389 | 128.5578 | 50,009 | | 0.001606 |
|  | 5.8S | 92 | 137.6522 | 12,664 | | 0.000407 |
|  | 5S | 1,220 | 85.33443 | 104,108 | | 0.003344 |
| snRNA | snRNA | 568 | 120.5141 | 68,452 | | 0.002199 |
|  | CD-box | 326 | 109.9141 | 35,832 | | 0.001151 |
|  | HACA-box | 89 | 130.4494 | 11,610 | | 0.000373 |
|  | splicing | 153 | 137.3203 | 21010 | | 0.000675 |

**Supplementary Table 13. Summary of BUSCOs gene evaluation.**

|  |  | |
| --- | --- | --- |
|  | Number | Percent (%) |
| Complete and single-copy BUSCOs* (S) | 1,209 | 87.9 |
| Complete and duplicated BUSCOs (D) | 73 | 5.3 |
| Fragmented BUSCOs (F) | 43 | 3.1 |
| Missing BUSCOs (M) | 50 | 3.7 |

* The database was embryophyta_odb10 (Creation date: 2017-12-01, number of species: 60, number of BUSCOs: 1375)

**Supplementary Table 14. Number of effects by type and region.**

| **Type (alphabetical order)** | **Count** | **Percent** |
| --- | --- | --- |
| downstream_gene_variant | 144,416 | 18.42% |
| initiator_codon_variant | 12 | 0.00% |
| intergenic_region | 262,127 | 33.44% |
| intron_variant | 80,157 | 10.23% |
| missense_variant | 105,976 | 13.52% |
| splice_acceptor_variant | 149 | 0.02% |
| splice_donor_variant | 141 | 0.02% |
| splice_region_variant | 3,144 | 0.40% |
| start_lost | 106 | 0.01% |
| stop_gained | 941 | 0.12% |
| stop_lost | 154 | 0.02% |
| stop_retained_variant | 131 | 0.02% |
| synonymous_variant | 112,662 | 14.37% |
| upstream_gene_variant | 73,738 | 9.41% |

**Supplementary Table 15. Summary of mean values and Tukey's test of catechins against leaf size, leaf shape, populations and groups.**

| **Tissue^a^** | **Catechin^b^** | **Leaf size^c^** | | **Tree shape^c^** | | **Populations^c^** | | | | | **Groups^c^** | |
| --- | --- | --- | --- | --- | --- | --- | --- | --- | --- | --- | --- | --- |
|  |  | **Large leaf** | **Median leaf** | **Arbor** | **Shrub** | **Pop1** | **Pop2** | **Pop3** | **Pop4** | **Pop5** | **Cultivar** | **Landrace** |
| YL | GA | 0.39036,a | 0.43251,a | 0.43697,a | 0.39794,a | 0.45406,a | 0.40102,a | 0.44457,a | 0.3507,a | 0.46773,a | 0.42297,a | 0.46322,a |
| YL | GC | 3.37115,a | 3.0239,a | 3.1836,a | 2.86553,a | 3.20227,ab | 3.61701,a | 2.44114,b | 3.38301,ab | 2.98899,ab | 3.11035,a | 2.62149,a |
| YL | EGC | 33.68988,b | 38.41774,a | 37.28981,a | 38.21338,a | 34.00767,a | 39.9002,a | 43.6205,a | 35.12659,a | 33.79239,a | 37.6177,a | 36.73325,a |
| YL | C | 3.95441,a | 3.15295,a | 3.37023,a | 3.12878,a | 4.47843,a | 4.6118,a | 2.29897,b | 2.9854,b | 2.80497,b | 3.31301,a | 2.97418,a |
| YL | EGCG | 93.9208,a | 96.90768,a | 95.80493,a | 97.6587,a | 87.84292,b | 102.77159,a | 90.31893,b | 99.30392,ab | 99.61246,ab | 96.29377,a | 97.90449,a |
| YL | EC | 3.76028,a | 3.68947,a | 3.75796,a | 3.57612,a | 4.25749,a | 4.06239,a | 3.53487,a | 3.40843,a | 3.50118,a | 3.73413,a | 3.09371,a |
| YL | GCG | 1.17806,a | 0.02185,b | 0.32953,a | 0,a | 1.55008,a | 0.09306,a | 0,a | 0,a | 0,a | 0.24033,a | 0,a |
| YL | ECG | 41.28404,a | 40.71668,a | 41.26399,a | 39.81193,a | 44.51454,a | 46.46649,a | 31.94892,a | 42.39377,a | 41.62359,a | 41.03214,a | 36.74929,a |
| YL | CG | NA | NA | NA | NA | NA | NA | NA | NA | NA | NA | NA |
| TL | GA | 0.08155,a | 0.088,a | 0.09144,a | 0.0765,a | 0.09134,a | 0.09013,a | 0.07132,a | 0.07451,a | 0.10661,a | 0.08746,a | 0.07529,a |
| TL | GC | 3.15369,a | 2.61181,a | 2.84661,a | 2.39719,a | 3.27196,a | 3.23617,a | 2.38251,a | 2.1284,a | 2.75197,a | 2.72397,a | 2.41613,a |
| TL | EGC | 41.69936,a | 42.40607,a | 42.09686,a | 42.69272,a | 41.46241,a | 42.96204,a | 45.66447,a | 38.76413,a | 41.70184,a | 42.03365,a | 46.95822,a |
| TL | C | 1.78287,a | 1.66351,a | 1.81264,a | 1.39648,a | 2.45622,a | 2.32186,a | 1.36402,ab | 1.18802,b | 1.45375,ab | 1.67803,a | 1.81381,a |
| TL | EGCG | 65.27709,a | 71.76185,a | 70.06818,a | 71.81224,a | 55.87992,b | 73.54078,a | 70.29756,a | 72.25151,a | 75.48854,a | 70.01461,b | 81.79961,a |
| TL | EC | 3.9527,a | 3.79554,a | 3.87445,a | 3.7089,a | 4.76516,a | 4.4309,ab | 3.50395,bc | 3.24026,c | 3.58865,ac | 3.83743,a | 3.56076,a |
| TL | GCG | 0.78731,a | 0.01265,b | 0.21791,a | 0,a | 1.03593,a | 0.05388,a | 0,a | 0,a | 0,a | 0.15893,a | 0,a |
| TL | ECG | 22.8766,a | 23.5726,a | 23.86338,a | 22.512,a | 26.66024,a | 27.57372,a | 19.25796,a | 22.16236,a | 23.46566,a | 23.55478,a | 21.42536,a |
| TL | CG | NA | NA | NA | NA | NA | NA | NA | NA | NA | NA | NA |
| ML | GA | 0.00475,a | 0.00063,b | 0.0011,a | 0.00196,a | 0.00181,a | 0,a | 0.00356,a | 0,a | 0.00116,a | 0.00119,a | 0.00472,a |
| ML | GC | 2.42234,a | 1.66893,b | 1.89556,a | 1.59574,a | 2.41814,a | 1.98633,a | 1.86452,a | 1.63509,a | 1.38922,a | 1.81775,a | 1.53215,a |
| ML | EGC | 33.15281,a | 32.34603,a | 31.95214,a | 33.70363,a | 29.79517,a | 30.3241,a | 36.50024,a | 31.40713,a | 32.68395,a | 32.53606,a | 31.6169,a |
| ML | C | 1.36602,a | 0.63748,b | 0.85132,a | 0.57864,a | 1.64041,a | 0.56644,b | 0.72262,b | 0.65362,b | 0.57365,b | 0.78453,a | 0.44528,a |
| ML | EGCG | 43.65342,a | 46.05284,a | 45.19499,a | 46.59298,a | 38.41862,b | 47.41021,a | 44.63985,ab | 43.96631,ab | 50.50521,a | 45.32653,b | 51.2834,a |
| ML | EC | 3.06906,a | 2.82007,a | 2.86328,a | 2.86738,a | 3.86774,a | 2.54235,a | 2.77695,a | 2.8533,a | 2.65371,a | 2.88644,a | 2.44835,a |
| ML | GCG | 0.81,a | 0.12712,b | 0.33234,a | 0.06122,a | 1.00949,a | 0.13832,a | 0.12066,a | 0.08656,a | 0.16575,a | 0.25842,a | 0.07141,a |
| ML | ECG | 15.08349,a | 14.33881,a | 14.80158,a | 13.72784,a | 18.66406,a | 14.53341,ab | 12.3522,b | 14.24462,ab | 14.31776,ab | 14.54255,a | 13.12734,a |
| ML | CG | 0.05389,a | 0.05146,a | 0.04,a | 0.07874,a | 0.03097,a | 0.0832,a | 0.01135,a | 0.07291,a | 0.06139,a | 0.05207,a | 0.04853,a |

a. YL: young leaf; TL: third leaf; ML: mature leaf

b. CG: Catechin gallate; GA: gallic acid; GCG: Gallocatechin gallate; C: catechin; GC: gallocatechin; EC: epicatechin; ECG: Epicatechin gallate; EGC: Epigallocatechin; EGCG: Epigallocatechin gallate

c. Numbers indicate the mean levels of corresponding metabolites. The letters indicate the statistical significance of Tukey’s test (*P* < 0.05)

**Supplementary Table 16. Adjusted *P*-value (Bonferroni) of likelihood radial test for catechins.**

| **Tissue** | **Catechin and GA** | **Tree shape** | **Leaf size** | **Sub-populations** | **Group** |
| --- | --- | --- | --- | --- | --- |
| YL | GA | 1.000 | 1.000 | 1.000 | 1.000 |
| YL | GC | 1.000 | 1.000 | 1.000 | 1.000 |
| YL | EGC | 1.000 | 1.000 | 1.000 | 1.000 |
| YL | C | 1.000 | 1.000 | 0.002 | 0.261 |
| YL | EGCG | 1.000 | 1.000 | 1.000 | 1.000 |
| YL | EC | 1.000 | 1.000 | 1.000 | 1.000 |
| YL | GCG | 1.000 | 0.826 | 1.000 | 1.000 |
| YL | ECG | 1.000 | 1.000 | 1.000 | 1.000 |
| YL | CG | NA | NA | NA | NA |
| TL | GA | 1.000 | 1.000 | 1.000 | 1.000 |
| TL | GC | 1.000 | 1.000 | 1.000 | 1.000 |
| TL | EGC | 1.000 | 1.000 | 1.000 | 1.000 |
| TL | C | 1.000 | 1.000 | 0.037 | 0.893 |
| TL | EGCG | 1.000 | 1.000 | 0.094 | 0.635 |
| TL | EC | 1.000 | 1.000 | 0.199 | 1.000 |
| TL | GCG | 1.000 | 0.815 | 1.000 | 1.000 |
| TL | ECG | 1.000 | 1.000 | 1.000 | 1.000 |
| TL | CG | NA | NA | NA | NA |
| ML | GA | 1.000 | 1.000 | 1.000 | 1.000 |
| ML | GC | 1.000 | 0.315 | 1.000 | 1.000 |
| ML | EGC | 1.000 | 1.000 | 1.000 | 1.000 |
| ML | C | 1.000 | 0.028 | 0.073 | 1.000 |
| ML | EGCG | 1.000 | 1.000 | 0.351 | 1.000 |
| ML | EC | 1.000 | 1.000 | 1.000 | 1.000 |
| ML | GCG | 1.000 | 1.000 | 1.000 | 1.000 |
| ML | ECG | 1.000 | 1.000 | 1.000 | 1.000 |
| ML | CG | 1.000 | 1.000 | 1.000 | 1.000 |

**Supplementary Table 17. Primers for amplifying cDNA of three candidate genes.**

| **Gene** | **Left primer (5’-3’)** | **Right primer (5’-3’)** |
| --- | --- | --- |
| CsF3'5'H | ATGGCCCTAGACACAGTCTTCCT | TTAAGCAGCATAAGCATTTGGAG |
| CsANR | ATGGCAATGGCAATGGCAACA | TCAGTTCTGCAAAAGCCCCTTA |
| CsMYB5 | ATGAGGCAGCCATCATCATCAT | TCAGATCTGCTTTTCCATGTGAT |

**Supplementary Table 18. Enzyme efficiency of CsANRa and CsANRb.**

| Gene | Km (μM) | Vmax (nM^-1^min·mg) | Kcat (min^-1^) | Enzyme efficiency (S^-1^ × μM^-1^) |
| --- | --- | --- | --- | --- |
| AtANR | 18.15 | 10.06 | 6.03 | 0.33 |
| CsANRa | 149.24 | 26.81 | 16.06 | 0.11 |
| CsANRb | 14.15 | 8.93 | 5.35 | 0.38 |

**Supplementary References**

1 Bolger, A. M., Lohse, M. & Usadel, B. Trimmomatic: a flexible trimmer for Illumina sequence data. *Bioinformatics* **30**, 2114-2120 (2014).

2 Dolezel, J., Greilhuber, J. & Suda, J. Estimation of nuclear DNA content in plants using flow cytometry. *Nat. Protoc.* **2**, 2233-2244, doi:10.1038/nprot.2007.310 (2007).

3 Liu, B. *et al.* Estimation of genomic characteristics by analyzing k-mer frequency in de novo genome projects. *arXiv preprint arXiv:1308.2012* (2013).

4 Chin, C.-S. *et al.* Phased diploid genome assembly with single-molecule real-time sequencing. *Nat. Methods* **13**, 1050 (2016).

5 Huang, S., Kang, M. & Xu, A. HaploMerger2: rebuilding both haploid sub-assemblies from high-heterozygosity diploid genome assembly. *Bioinformatics* **33**, 2577-2579 (2017).

6 Li, H. & Durbin, R. Fast and accurate short read alignment with Burrows–Wheeler transform. *Bioinformatics* **25**, 1754-1760 (2009).

7 Walker, B. J. *et al.* Pilon: an integrated tool for comprehensive microbial variant detection and genome assembly improvement. *PloS one* **9**, e112963 (2014).

8 Li, R., Li, Y., Kristiansen, K. & Wang, J. SOAP: short oligonucleotide alignment program. *Bioinformatics* **24**, 713-714 (2008).

9 Zhu, W. *et al.* Altered chromatin compaction and histone methylation drive non-additive gene expression in an interspecific Arabidopsis hybrid. *Genome Biol.* **18**, 157 (2017).

10 Wang, C. *et al.* Genome-wide analysis of local chromatin packing in Arabidopsis thaliana. *Genome Res.* **25**, 246-256 (2015).

11 Langmead, B. & Salzberg, S. L. Fast gapped-read alignment with Bowtie 2. *Nat. Methods* **9**, 357 (2012).

12 Servant, N. *et al.* HiC-Pro: an optimized and flexible pipeline for Hi-C data processing. *Genome Biol.* **16**, 259 (2015).

13 Durand, N. C. *et al.* Juicer provides a one-click system for analyzing loop-resolution Hi-C experiments. *Cell Syst.* **3**, 95-98 (2016).

14 Dudchenko, O. *et al.* De novo assembly of the Aedes aegypti genome using Hi-C yields chromosome-length scaffolds. *Science* **356**, 92-95 (2017).

15 Ma, J.-Q. *et al.* Large-scale SNP discovery and genotyping for constructing a high-density genetic map of tea plant using specific-locus amplified fragment sequencing (SLAF-seq). *PLoS One* **10** (2015).

16 Xu, L.-Y. *et al.* High-density SNP linkage map construction and QTL mapping for flavonoid-related traits in a tea plant (Camellia sinensis) using 2b-RAD sequencing. *BMC genomics* **19**, 955 (2018).

17 Simão, F. A., Waterhouse, R. M., Ioannidis, P., Kriventseva, E. V. & Zdobnov, E. M. BUSCO: assessing genome assembly and annotation completeness with single-copy orthologs. *Bioinformatics* **31**, 3210-3212 (2015).

18 Kent, W. J. BLAT--the BLAST-like alignment tool. *Genome research* **12**, 656-664, doi:10.1101/gr.229202 (2002).

19 Tang, H. *et al.* ALLMAPS: robust scaffold ordering based on multiple maps. *Genome Biol.* **16**, 3 (2015).

20 Initiative, A. G. Analysis of the genome sequence of the flowering plant Arabidopsis thaliana. *Nature* **408**, 796 (2000).

21 Huang, S. *et al.* Draft genome of the kiwifruit Actinidia chinensis. *Nat. Commun.* **4**, 1-9 (2013).

22 Denoeud, F. *et al.* The coffee genome provides insight into the convergent evolution of caffeine biosynthesis. *Science* **345**, 1181-1184 (2014).

23 Tuskan, G. A. *et al.* The genome of black cottonwood, Populus trichocarpa (Torr. & Gray). *Science* **313**, 1596-1604 (2006).

24 Consortium, T. G. The tomato genome sequence provides insights into fleshy fruit evolution. *Nature* **485**, 635 (2012).

25 Jaillon, O. *et al.* The grapevine genome sequence suggests ancestral hexaploidization in major angiosperm phyla. *Nature* **449**, 463 (2007).

26 Slater, G. S. C. & Birney, E. Automated generation of heuristics for biological sequence comparison. *BMC bioinformatics* **6**, 31 (2005).

27 Birney, E., Clamp, M. & Durbin, R. GeneWise and genomewise. *Genome Res.* **14**, 988-995 (2004).

28 Kim, D., Langmead, B. & Salzberg, S. L. HISAT: a fast spliced aligner with low memory requirements. *Nature methods* **12**, 357-360, doi:10.1038/nmeth.3317 (2015).

29 Pertea, M. *et al.* StringTie enables improved reconstruction of a transcriptome from RNA-seq reads. *Nat. Biotechnol.* **33**, 290 (2015).

30 Wu, T. D. & Watanabe, C. K. GMAP: a genomic mapping and alignment program for mRNA and EST sequences. *Bioinformatics* **21**, 1859-1875 (2005).

31 Keller, O., Kollmar, M., Stanke, M. & Waack, S. A novel hybrid gene prediction method employing protein multiple sequence alignments. *Bioinformatics* **27**, 757-763 (2011).

32 Stanke, M. *et al.* AUGUSTUS: ab initio prediction of alternative transcripts. *Nucleic Acids Res.* **34**, W435-W439 (2006).

33 Johnson, A. D. *et al.* SNAP: a web-based tool for identification and annotation of proxy SNPs using HapMap. *Bioinformatics* **24**, 2938-2939 (2008).

34 Holt, C. & Yandell, M. MAKER2: an annotation pipeline and genome-database management tool for second-generation genome projects. *BMC bioinformatics* **12**, 491 (2011).

35 Boeckmann, B. *et al.* The SWISS-PROT protein knowledgebase and its supplement TrEMBL in 2003. *Nucleic Acids Res.* **31**, 365-370 (2003).

36 Koonin, E. V. *et al.* A comprehensive evolutionary classification of proteins encoded in complete eukaryotic genomes. *Genome Biol.* **5**, R7 (2004).

37 Kanehisa, M. & Goto, S. KEGG: kyoto encyclopedia of genes and genomes. *Nucleic Acids Res.* **28**, 27-30 (2000).

38 Mitchell, A. L. *et al.* InterPro in 2019: improving coverage, classification and access to protein sequence annotations. *Nucleic Acids Res.* **47**, D351-D360, doi:10.1093/nar/gky1100 (2019).

39 Ashburner, M. *et al.* Gene ontology: tool for the unification of biology. *Nat. Genet.* **25**, 25 (2000).

40 Altschul, S. F., Gish, W., Miller, W., Myers, E. W. & Lipman, D. J. Basic local alignment search tool. *J Mol Biol* **215**, 403-410, doi:10.1016/S0022-2836(05)80360-2 (1990).

41 Lowe, T. M. & Eddy, S. R. tRNAscan-SE: a program for improved detection of transfer RNA genes in genomic sequence. *Nucleic Acids Res.* **25**, 955-964 (1997).

42 Griffiths-Jones, S. *et al.* Rfam: annotating non-coding RNAs in complete genomes. *Nucleic Acids Res.* **33**, D121-D124 (2005).

43 Sedlazeck, F. J. *et al.* Accurate detection of complex structural variations using single-molecule sequencing. *Nat. Methods* **15**, 461-468 (2018).

44 Li, H. *et al.* The Sequence Alignment/Map format and SAMtools. *Bioinformatics* **25**, 2078-2079 (2009).

45 Ye, J. *et al.* WEGO 2.0: a web tool for analyzing and plotting GO annotations, 2018 update. *Nucleic Acids Res.* **46**, W71-W75 (2018).

46 Maere, S., Heymans, K. & Kuiper, M. BiNGO: a Cytoscape plugin to assess overrepresentation of gene ontology categories in biological networks. *Bioinformatics* **21**, 3448-3449, doi:10.1093/bioinformatics/bti551 (2005).

47 Shannon, P. *et al.* Cytoscape: a software environment for integrated models of biomolecular interaction networks. *Genome Res* **13**, 2498-2504, doi:10.1101/gr.1239303 (2003).

48 McKenna, A. *et al.* The Genome Analysis Toolkit: A MapReduce framework for analyzing next-generation DNA sequencing data. *Genome Res.* **20**, 1297-1303 (2010).

49 Cingolani, P. *et al.* A program for annotating and predicting the effects of single nucleotide polymorphisms, SnpEff: SNPs in the genome of Drosophila melanogaster strain w1118; iso-2; iso-3. *Fly* **6**, 80-92 (2012).

50 Browning, S. R. & Browning, B. L. Rapid and accurate haplotype phasing and missing-data inference for whole-genome association studies by use of localized haplotype clustering. *Am. J. Hum. Genet.* **81**, 1084-1097 (2007).

51 Kolde, R. pheatmap: Pretty Heatmaps. R package version 1.0. 8. (2015).

52 Bates, D., Machler, M., Bolker, B. M. & Walker, S. C. Fitting Linear Mixed-Effects Models Using lme4. *Journal of Statistical Software* **67**, 1-48 (2015).

53 Emms, D. M. & Kelly, S. OrthoFinder: phylogenetic orthology inference for comparative genomics. *Genome Biol.* **20**, 238, doi:10.1186/s13059-019-1832-y (2019).

54 Emms, D. M. & Kelly, S. OrthoFinder: solving fundamental biases in whole genome comparisons dramatically improves orthogroup inference accuracy. *Genome Biol.* **16**, 157, doi:10.1186/s13059-015-0721-2 (2015).

55 Emms, D. M. & Kelly, S. STRIDE: Species Tree Root Inference from Gene Duplication Events. *Mol. Biol. Evol.* **34**, 3267-3278, doi:10.1093/molbev/msx259 (2017).

56 Edgar, R. C. MUSCLE: a multiple sequence alignment method with reduced time and space complexity. *BMC bioinformatics* **5**, 113, doi:10.1186/1471-2105-5-113 (2004).

57 Price, M. N., Dehal, P. S. & Arkin, A. P. FastTree 2--approximately maximum-likelihood trees for large alignments. *PLoS One* **5**, e9490, doi:10.1371/journal.pone.0009490 (2010).

58 Hahn, M. W., De Bie, T., Stajich, J. E., Nguyen, C. & Cristianini, N. Estimating the tempo and mode of gene family evolution from comparative genomic data. *Genome Res.* **15**, 1153-1160, doi:10.1101/gr.3567505 (2005).

59 De Bie, T., Cristianini, N., Demuth, J. P. & Hahn, M. W. CAFE: a computational tool for the study of gene family evolution. *Bioinformatics* **22**, 1269-1271 (2006).

60 Conway, J. R., Lex, A. & Gehlenborg, N. UpSetR: an R package for the visualization of intersecting sets and their properties. *Bioinformatics* **33**, 2938-2940, doi:10.1093/bioinformatics/btx364 (2017).

61 Wang, K., Li, M. & Hakonarson, H. ANNOVAR: functional annotation of genetic variants from high-throughput sequencing data. *Nucleic Acids Res.* **38**, e164-e164 (2010).

62 Zhang, Y. *et al.* Rapid Identification of Protein-Protein Interactions in Plants. *Curr. Opin. Plant Biol.* **4**, e20099, doi:10.1002/cppb.20099 (2019).

63 Zhang, Y. *et al.* The Extra-Pathway Interactome of the TCA Cycle: Expected and Unexpected Metabolic Interactions. *Plant Physiol.* **177**, 966-979, doi:10.1104/pp.17.01687 (2018).

64 Youjun Zhang, M. C., Beata Siemiatkowska, M. R. T., Yue Jing, V. S., Jianghua Zhang, Y. S. & Fernie, A. R. A Highly Efficient Agrobacterium-Mediated Method for Transient Gene Expression and Functional Studies in Multipe Plant Species. *Plant Communications*, doi:https://doi.org/10.1016/j.xplc.2020.100028 (2020).

65 Xie, D. Y., Sharma, S. B. & Dixon, R. A. Anthocyanidin reductases from Medicago truncatula and Arabidopsis thaliana. *Archives of biochemistry and biophysics* **422**, 91-102, doi:10.1016/j.abb.2003.12.011 (2004).
