# Supplementary material for: Genome assembly of wild tea tree DASZ reveals pedigree and selection history of tea varieties
Source: Nat Commun. 2020 Jul 24;11:3719. doi: 10.1038/s41467-020-17498-6 (PMC7381669; doi:10.1038/s41467-020-17498-6)
Supplement: Supplementary file 3 — Reporting Summary [file 41467_2020_17498_MOESM3_ESM.pdf]

nature research

Corresponding author(s): Weiwei Wen

Last updated by author(s): Jun 17, 2020

Reporting Summary

Nature Research wishes to improve the reproducibility of the work that we publish. This form provides structure for consistency and transparency in reporting. For further information on Nature Research policies, see Authors & Referees and the Editorial Policy Checklist .

Statistics

For all statistical analyses, confirm that the following items are present in the figure legend, table legend, main text, or Methods section.

n/a	Confirmed	
☐	☒	The exact sample size (*n*) for each experimental group/condition, given as a discrete number and unit of measurement
☐	☒	A statement on whether measurements were taken from distinct samples or whether the same sample was measured repeatedly
☐	☒	The statistical test(s) used AND whether they are one- or two-sided<br>*Only common tests should be described solely by name; describe more complex techniques in the Methods section.*
☐	☒	A description of all covariates tested
☐	☒	A description of any assumptions or corrections, such as tests of normality and adjustment for multiple comparisons
☐	☒	A full description of the statistical parameters including central tendency (e.g. means) or other basic estimates (e.g. regression coefficient) AND variation (e.g. standard deviation) or associated estimates of uncertainty (e.g. confidence intervals)
☐	☒	For null hypothesis testing, the test statistic (e.g. *F*, *t*, *r*) with confidence intervals, effect sizes, degrees of freedom and *P* value noted<br>*Give P values as exact values whenever suitable.*
☒	☐	For Bayesian analysis, information on the choice of priors and Markov chain Monte Carlo settings
☒	☐	For hierarchical and complex designs, identification of the appropriate level for tests and full reporting of outcomes
☐	☒	Estimates of effect sizes (e.g. Cohen's *d*, Pearson's *r*), indicating how they were calculated

Our web collection on statistics for biologists contains articles on many of the points above.

Software and code

Policy information about availability of computer code

Data collection	No software was used for data collection
Data analysis	R (v3.6) is used in all statistical analysis;Trimmomatic (v0.36) is used for filtering adapters and low quality reads; Jellyfish (v1.1.12) was used to estimate genome size; Falcon (v0.3.0) is used for genome assembly; HaploMerger2 (v20180603) was used for reducing redundancy. Arrow (https://github.com/PacificBiosciences/SMRT-Link) and Pilon (v1.22) are used to correct genome assembly; SOAPaligner (v2.21) is used for evaluation of GC contents; BWA (v0.7.16a) is used for mapping illumina reads; bowtie2 (v2.2.5) is used for mapping Hi-C reads to draft contigs; HiC-Pro pipeline (v2.5.0), Juicer (v1.0) and 3d-dna (v2.0) were applied for assembly DASZ genome using Hi-C reads; BUSCO (v3.0), BLAT (v0.36) and ALLMAPS (v180626) are used for evaluation of genome assembly; RepeatMasker (v4.07), ProteinMask (v4.07), RepeatModeler (v2.0.1), LTR-FINDER (v1.06) and TRF (v4.09) are used for annotation the repeat sequences in DASZ. hisat (v2-2.1.0), STAR (2.7.1a), StringTie (v1.3.4d) and RSEM (v1.3.3) are used for analysis illumina RNA-seq data; Gmap (v2014-08-04), Pasa (v2.3.3), Exonerate (v2.2.0), Genewise (v2.4.1), Augustus (v3.3.1), SNAP (v2006-07-28) and MAKER (v2.31.10) are used for prediction of gene models of DASZ; SwissProt (201709), TrEMBL (201709), NR (20170924), KOG (20090331), KEGG (v84) Inerpro (v5.16-55.0), GO databases (20181101) and BLAST (v2.2.28) were used for gene annotation. tRNAscan-SE (v1.3.1) and Rfam (v12.0) were used for annotation of non-coding genes of DASZ; ngmlr (v0.2.7) and sniffles (v1.0.10) were used for SV calling; GATK (v4.1.2.0) is used for SNP calling; Cervus (v3.0) is used for parentage analysis; PLINK (v1.90b4) is used for filtering SNPs; EIGENSOFT (v7.21) is used for PCA analysis; ADMIXTURE (v1.3.0) and CLUMPP (v1.1.2) are used for analysis population structure; RAxML (v8.2.12) and iTOL (v5) are used for phylogenetic analysis; PopLDdecay (v3.40) is used for analysis of LD decay; beagle (v5.0) is used for imputation missing genotypes; EMMAX (2010) and GEC (v0.2) are used for GWAS; Orthofinder (v2.3.3) is used for analysis cross-species orthology relationships; MUSCLE (v3.8.31) was used for multiple alignment. FastTree (v2.0) was used for constructing phylogenetic tree. CAFE (v4.2) was used for studying gene evolution. UpSetR (v1.4.0) was used to generate intersection plot. GO annotation was conducted using WeGO website (v2.0). pheatmap (1.0.12) was used to plot heatmap. lme4 (1.1-23) was used for performing linear mixed effects analysis. ANNOVAR (2019Oct24) was used for annotation of SNP effects.

For manuscripts utilizing custom algorithms or software that are central to the research but not yet described in published literature, software must be made available to editors/reviewers. We strongly encourage code deposition in a community repository (e.g. GitHub). See the Nature Research guidelines for submitting code & software for further information.

Data

Policy information about availability of data

All manuscripts must include a data availability statement. This statement should provide the following information, where applicable:

- Accession codes, unique identifiers, or web links for publicly available datasets
- A list of figures that have associated raw data
- A description of any restrictions on data availability

All datasets in this study were deposited to National Genomics Data Center at the Beijing Institute of Genomics, Chinese Academy of Sciences under the following accession codes: DASZ PacBio long reads, CRA002210 ; DASZ Hi-C data, CRA002233 ; DASZ Illumina short reads, CRA002211 ; Illumina short reads of Fudingdabai, CRA002227 ; RNA-Seq of 217 tea plant accessions, CRA002228 ; Genome assembly and annotation files of DASZ, GWHABKB00000000. Metabolic data was provided in the Supplementary Data 10 and source file data was provided in Source Data. Source data for all the Figures in main text and Supplementary information were provided in either Source Data or Supplementary Data.

Field-specific reporting

Please select the one below that is the best fit for your research. If you are not sure, read the appropriate sections before making your selection.

[x] Life sciences   [ ] Behavioural & social sciences   [ ] Ecological, evolutionary & environmental sciences

For a reference copy of the document with all sections, see nature.com/documents/nr-reporting-summary-flat.pdf

Life sciences study design

All studies must disclose on these points even when the disclosure is negative.

Sample size	No sample size calculation was performed. The sample size choosing is comparable to the previous GWAS and population genetic research. For example, Zhang et al., 2017 used 240 lettuce accessions for GWAS study. Crowell et al., 2016 performed GWAS with 242 tropical rice accessions.
Data exclusions	During population genetic analysis, variants with minor allele frequency (MAF) less than 5% and missing rate greater than 10% were excluded. This filtration will improve the reliability of the analysis. The exclusion criteria were widely used in population genetic study and were pre-established. During association mapping, those variants with minor allele frequency (MAF) less than 10% were excluded and those samples were obviously outlier in PCA plot were removed. Because current statistic model is not suitable for these minor alleles and outliers. The exclusion criteria were widely used and were pre-established.
Replication	Large scale metabolic profiling data were obtained from replicated experimental studies. Subsequent enzyme activity assay experiments have demonstrated reproducibility.
Randomization	10 sets of 500 SNPs with polymorphic information content (PIC) greater than 0.35 were randomly selected for parentage analysis. In order to exam the accuracy of SNP calling and filtration, we randomly picked 500 SNPs and two accessions for sanger sequencing.
Blinding	The names of tea accessions were blinded to the researchers who performed RNA-seq. Gene names and allelic information are blinded to the researcher who conducted transient overexpression and enzyme activity assay

Reporting for specific materials, systems and methods

We require information from authors about some types of materials, experimental systems and methods used in many studies. Here, indicate whether each material, system or method listed is relevant to your study. If you are not sure if a list item applies to your research, read the appropriate section before selecting a response.

Materials & experimental systems

n/a	Involved in the study
[x]	[ ] Antibodies
[x]	[ ] Eukaryotic cell lines
[x]	[ ] Palaeontology
[x]	[ ] Animals and other organisms
[x]	[ ] Human research participants
[x]	[ ] Clinical data

Methods

n/a	Involved in the study
[x]	[ ] ChIP-seq
[x]	[ ] Flow cytometry
[x]	[ ] MRI-based neuroimaging
