# Supplementary material for: Genome assembly of wild tea tree DASZ reveals pedigree and selection history of tea varieties
Source: Nat Commun. 2020 Jul 24;11:3719. doi: 10.1038/s41467-020-17498-6 (PMC7381669; doi:10.1038/s41467-020-17498-6)
Supplement: Supplementary file 4 — Description of Additional Supplementary Files [file 41467_2020_17498_MOESM4_ESM.docx]

**Description of Additional Supplementary Files**

File Name: **Supplementary Data 1**

Description: List of SVs identified by mapping the Pacbio sequences of DASZ to DASZ genome.

File Name: **Supplementary Data 2**

Description: List of genes affected by hSVs identified in DASZ. This table contains SVs overlapping with genes.

File Name: **Supplementary Data 3**

Description: GO enrichment analysis results of genes overlapped with hSVs identified by mapping DASZ Pacbio reads to DASZ genome.

File Name: **Supplementary Data 4**

Description: List of SVs identified by mapping the Pacbio sequences of Shuchazao to Shuchazao genome.

File Name: **Supplementary Data 5**

Description: List of SVs identified between DASZ and Shuchazao by mapping the Pacbio sequences of Shuchazao to DASZ genome.

File Name: **Supplementary Data 6**

Description: Annotation of genes overlapped with SVs identified between DASZ and Shuchazao.

File Name: **Supplementary Data 7**

Description: Information of accessions collected in this study including the origin, sample type, morphological information and pedigree records.

File Name: **Supplementary Data 8**

Description: Summary of RNA-seq results including number of raw reads, number of clean reads and overall alignment rate.

File Name: **Supplementary Data 9**

Description: Results of parentage analysis.

File Name: **Supplementary Data 10**

Description: Contents (mg·g^-1^ dry weight) of catechins and gallic acid in three leaf tissues of tea population.

File Name: **Supplementary Data 11**

Description: List of orthogroups for the biosynthetic pathway of non-galloylated and galloylated catechins. This table contains the list of the orthogroups related to the structural genes of catechin biosynthesis. Genes on the same row constitute an orthogroup. Orthogroups are defined as groups of genes descending from a single gene in the last common ancestor (LCA) of a group of species.

File Name: **Supplementary Data 12**

Description: Location of duplication events within the gene family of SCPL Acyltransferases (OG0000034).

File Name: **Supplementary Data 13**

Description: List of the gene families which are rapidly evolving across each branch of the phylogeny (see tree below the table for the branch numbers).

File Name: **Supplementary Data 14**

Description: List of the gene families which are rapidly evolving in DASZ only. The table contains the gene families (orthogroups) which are rapidly evolving (expansion >4 or contraction <4) only in DASZ with respect to the other genomes shown in the intersection plot below.

File Name: **Supplementary Data 15**

Description: List of mQTL identified by GWAS.

File Name: **Supplementary Data 16**

Description: List of genes located in the mQTL candidate region. The table contains gene annotations and correlation between gene expression level and metabolite content.

File Name: **Supplementary Data 17**

Description: Sequences of the two alleles of *CsANR*, *CsMYB5* and *CsF3’5’H* in this study.

File Name: **Supplementary Data 18**

Description: Metabolite changes (log_2_Foldchange) in transgenic tobacco leaves and *in vitro* enzyme assay using tea extraction. Value in red represents p < 0.05 in two-sided *t*-test.
