# Supplementary material for: Genome assembly of wild tea tree DASZ reveals pedigree and selection history of tea varieties
Source: Nat Commun. 2020 Jul 24;11:3719. doi: 10.1038/s41467-020-17498-6 (PMC7381669; doi:10.1038/s41467-020-17498-6)
Supplement: Supplementary file 2 — Peer Review [file 41467_2020_17498_MOESM2_ESM.pdf]

Editorial Note: Parts of this Peer Review File have been redacted as indicated to maintain the confidentiality of people who appear in the picture.

Reviewers' comments:

Reviewer #1 (Remarks to the Author):

The manuscript entitled “A chromosome-scale Camellia sinensis genome allows genetic, biochemical and evolutionary insights into the tea plant” characterized the assembly of a chromosome-scale reference genome for a wild tea landrace by using single-molecule real-time sequencing in combination with chromosome-contact and genetic maps. The results obtained from this study will contribute to further understanding of the biosynthesis of health-beneficial natural products in tea and future genetic improvement of tea. The following points are suggested to be improved.

(1) The possible existence of selective sweep among DASZ, Yunkang 10, and Shuchazao should be further studied to explore the role of artificial domestication.
(2) The authors needed to further explore 1121 genes affected by 1135 high impact SVs, to explore the metabolic pathways and metabolites that these SVs and genes might influence, and the significance of these SVs and genes possibly involved in biotic and abiotic stresses or artificial domestication.
(3) What does the higher gene copy number (ANS, DFR, LAR, F3'5'H) mean in the tea genome?
(4) The GWAS screened catechins and gallic acid synthesis related genes need be further tested for their function, such as in vitro enzymatic activity. The transcription factors regulating the formation of catechins also need be verified by corresponding transcriptional regulation experiments.
(5) Further experiments are needed to verify the significance of GWAS screening for non-synonymous SNPs on the function of the gene, such as affecting enzyme activity or other attributes?

Reviewer #2 (Remarks to the Author):

Manuscript 19-30844935 by Wen and colleagues presents a genome sequence for a Camellia sinensis accession performed at a very high quality. The manuscript also provides RNAseq data from numerous Chinese germplasm sources to identify SNPs that are used for diversity analyses. The data are useful, but the authors do not discover anything that was previously not known. Some results are not terribly believable, like the GWAS studies, and many of the conclusions are unjustified. I provide my specific comments below, in manuscript order.

Abstract:
(1) There is no such thing as a wild landrace. If it is a landrace, it is not wild. The idea, mentioned throughout, that landraces are somehow less subject to human selection is untrue and insulting to traditional farmers. Of course ancient farmers were selecting for improved traits. Most maize improvement, for instance, was performed by ancient farmers, and this is true for virtually all other crops. Hence, “the inconspicuous” sentence is nonsense. Moreover, if it so inconspicuous, then how did the authors perform GWAS studies?
(2) Their analysis is not “a step of the genetic improvement” at all, and it is certainly not “significant” in that regard.

Introduction:
(1) Why is the Scientific Data 2019 manuscript from Xia et al not mentioned? It has a more advanced Shuchazao assembly/annotation than does the 2018 manuscript that they reference.
(2) Test from “For example, nanomaterials….” is a non-sequitur. There are lots of proposed benefits from tea, but little to no proof, and listing a couple extreme and unproven examples is not a good use of the reader’s time.
(3) The idea that “the genetic diversity and population structure remain elusive due to limited number of molecular markers” is not true. You need fairly few markers for such an analysis, and certainly GBS

marker sets are more than sufficient. Actually, tea diversity has been fairly well studied already, but that does not mean that every variety has been investigated. These authors choose some additional varieties, but I don't think they find much that is new in a general sense, just new information about these varieties.

Results:
(1) As mentioned above, comparisons should be made to the assembly in the Scientific Data assembly/annotation, not the two old publications.
(2) Regarding 93.2% BUSCO scores, what were the BUSCO scores of earlier assemblies? I vaguely remember that the Scientific Data assembly gave something over 94%.
(3) The whole SV description is vague. (What is tau S?) I am not expert in this type of analysis, but the authors' statements of why homozygous SVs are possible artifacts or what hSVs mean (heterozygous I am guessing means hemizygous, at least for deletions). Are all SVs, as they define them, deletions, or do they include other types of rearrangements? Can they tell insertions from deletions, or are these all just considered indels?
(4) On lines 185-191, it seems odd that Fudingdabai is more closely related to Yunkang10 than to Shuchazao. Is Fudingdabai an assamica tea?
(5) It seems unlikely to me that the tea germplasm chosen was purely random. The authors see numerous relatives of Fudingdabai and a few others, but how do we know that this is an indication that many Chinese teas are related to these few lineages, or is it an outcome of what teas they chose to study? The results mentioned on lines 220-222 are strange, once again suggesting that the germplasm choice was not random.
(6) Regarding lines 236-237, how do the authors explain that other researchers have seen correlations both with geography (that is, Yunnan versus elsewhere) and with assamica (large leaf) versus sinensis (small leaf)? My guess is that their germplasm collection was so skewed (perhaps to small leaf varieties) that there really was little leaf size variation, and thus no real variance investigated.
(7) The metabolite analyses are only valid if performed on leaves harvested at the same leaf size, on the same day, and in a single field location. From the Methods, it seems unlikely that any of this was actually done.
(8) Gene family analyses have already been done in other publications.
(9) GWAS analysis is not valid unless the metabolite studies are done with samples collected in the same field, at the same leaf stage on the same day for all of the germplasm. I expect that this was not done. Many studies have shown major contributions of the environment to the metabolite profiles observed. Moreover, favorable genetic variants are only possible to detect if you have strong GWAS results. GWAS is prone to lots of false positives, as well, but it is hard to see how the authors took this into account.

Discussion
(1) The sentence "This huge difference…." Is not a very likely explanation. Population history (e.g., time spent homozygous versus heterozygous) is a more likely explanation.
(2) On lines 409-411, the conclusion made has already been shown to not be true in a general sense. The sinensis and assamica subspecies can be easily separated, and have shown little intercrossing, although that does not seem to be true for the apparently more skewed germplasm these authors chose to study.
(3) Lines 421-433 are interesting, but not really related to this study.
(4) Lines 434-437 are quite reasonable, but the authors should avoid the term "wild teas" because this is not a good name for landraces. There is nothing wild about them. Also, the authors should provide any proof for trees that have been harvested for hundreds of years. Is there any documentation of this, or is it just the opinion of local farmers.
(5) "and the protective effects of tea catechins on human health has been finely documented" greatly overstates the case. The possible value of catechins on human health if far from proven, much less "finely documented".

Reviewer #3 (Remarks to the Author):

In this report, Qiu et al. present a new reference genome sequence for tea, based on DNA from a landrace, contact and genetic maps, and long-read technologies. Additionally, RNA sequencing was performed on other accessions, and the authors used this information to investigate the history of cultivated varieties, as well as to find genes implicated in biosynthesis of important compounds. The authors concluded that flavor metabolites may not have been strongly targeted by selection.
The first interest of this manuscript is the quality of the genome sequence, particularly the continuity, which is a great improvement compared to the two draft versions already published. There are however no main findings derived from this high-quality assembly compared to the previous papers. The structural variations led to some conclusions that should be investigated more in-depth. The parentage study has to be improved, as well as the investigation of gene categories enrichment and percentage of variations between genomes. Taken together, the authors should investigate more their preliminary findings to show more convincingly the interest of this new reference.
Table 1 presents a comparison of annotation results, and it appears that there is no strong improvement compared to the sequence of Shuchazao besides the continuity of the contigs. The authors may make a better case why this sequence is really helping to address more questions than the previous one.
The analysis of SVs led to the finding of a large number of homozygous cases (line 144), which should be artefactual. It is important to sort out what is the problem. An assessment of error rates in long-read assemblies would be helpful. Many other conclusions, in particular about the heterozygous SVs differences with other varieties may be affected by this problem.
The parentage analysis is based on a selection of 500 random variants. Many conclusions are apparently based on this, although this number looks rather low for such a large genome. It is essential in this case to show that the same results can be obtained by other assortments of random markers.
Line 152, the category "regulation of cell death" is deemed enriched in heterozygous SVs. The authors should investigate this, and maybe other categories, in detail to derive functional insights.
In the discussion, the huge difference in SVs between Shuchazao and DASZ is explained by growth time. Other explanations have to be investigated, first the impact of the sequencing technologies, but also the sizes of the populations.

Reviewer #4 (Remarks to the Author):

In this manuscript, the authors report a new assembly of tea plant (Camellia sinensis), resequencing analysis based on RNA-seq data form 217 accessions. Compared with the original genome, the quality of this assembly is much higher. Chromosome-level genome assembly is important for understanding the origin of tea tree species, genome-assisted breeding, and genomic structure analysis. The authors used SNPs obtained from the sequences of leaf transcriptomes from 217 accessions to perform population genomics analysis, explore phylogenetic relationships of tea trees and perform genomic association analysis.
The manuscript is particularly useful for generating a genetic resource for the tea plant research community. I believe this genome assembled version will be the best genome assembled version of the species for quite some time. As a reference genome of Camellia sinensis, authors need to be more careful in confirming the quality of the assembly of the genome, especially in comparison with published assemblies, and in-depth analysis of the new assembly. Based on the reference genome, the author analyzes SV in the genome, but I think this analysis is open to question, both in terms of method and conclusion. Considering the large size of tea tree genome, in order to speed up and save costs, the author used RNA-seq data for SNP calling. These data also need to be verified to see if the results obtained by other methods are good consistency. The manuscript is in general clearly written.

Considering that tea tree has already released at least 2 different genome assemblies, the author needs to better show the uniqueness of this version and analyze more biological questions, so I suggest that this article needs a major revision.

There are other major issues that require attention:

1. Considering the complexity of the tea genome, especially the high content of repetitive sequences (over 80%), I strongly recommend that the author use different assembly strategies and software to list different assembly results and then determine a version, rather than using Falcon directly. HERA (Huilong Du,2019) is much better for high repetitive sequences genome. Cuna is other choice which is reported more accurate. Considering that this manuscript is based on genome assembly updates, the author is strongly recommended to use multiple software for assembly, and fully explain that this version is the best.
2. Hi-C data is useful when the chromosomes anchoring, but in the current high-quality plant reference genomes, Bionano data is also used to correct each other with Hi-C. Based on our past experience, simply using Hi-C in highly heterozygous genomes will lead many assembly errors, so it is recommended that the author use Bionano data for quality improvement, especially comparing the differences between the results of Bionano de novo assembly and the existing version.
3. High-quality reference genomes have large moderators for annotations of TEs sequences. In fact, the authors found that more than 87% of the tea tree genome is composed of TEs sequences. I hope that the author can analyze the TEs sequence, not just show the results of the annotation.
4. The authors used Pacbio reads mapping for SV calling and analysis, but these analyses were based on only one individual and the reference genome. Both the method and the biological interpretation were very inadequate. It is recommended to use more individuals, especially key accessions among 217 accessions.
5. In the analysis of the population genomics, due to the complex history of tea tree domestication, there are many asexual reproduction phenomena, and many varieties have the same name or are recorded incorrectly. How did the author ensure the accuracy of these samples?
6. The author uses RNA-seq sequences for analysis. According to the statistics of the data submitted by the author, the data volume of the sample ranges from 14M reads to 93M reads, and the mapping rate ranges from 80% to 98%. The author needs to explain the impact of these differences on their SNP calling. In addition, SNP filtering is very tricky. It is recommended that the author analyze each step of SNP calling and then perform SNP filtering based on the results.
7. As for this species, unlike Camellia sinensis (Linn.) Var. assamica, the leaf size changes little. The author divides the leaf size into three types. Is there any specific phenotypic measurement data support? Is this phenotype related to different growth environments? If the phenotype is not accurate, I recommend removing it in subsequent GWAS analysis.
8. Considering the high heterozygosity of the tea genome, part of the parentage analysis, it is recommended to use all biaslelic SNPs to ensure the credibility of the structure, in addition to the small number of samples, this part of the analysis results need more methods to support its feasibility. 38 samples have known breeding records. According to the results of population genomics analysis, can they be consistent with these breeding records?

I hope the author can explain and elaborate on the above issues, and more importantly, improve the quality of the manuscript and scientific issues through our discussion.

Reviewers' comments:

Reviewer #1 (Remarks to the Author):

The manuscript entitled “A chromosome-scale Camellia sinensis genome allows genetic, biochemical and evolutionary insights into the tea plant” characterized the assembly of a chromosome-scale reference genome for a wild tea landrace by using single-molecule real-time sequencing in combination with chromosome-contact and genetic maps. The results obtained from this study will contribute to further understanding of the biosynthesis of health-beneficial natural products in tea and future genetic improvement of tea. The following points are suggested to be improved.

We would firstly like to thank this reviewer for the positive assessment as well as the important critiques which we feel greatly aided us to considerably improve the manuscript.

(1) The possible existence of selective sweep among DASZ, Yunkang 10, and Shuchazao should be further studied to explore the role of artificial domestication.
Thanks for the nice comments. Firstly, a new favorable allele under strong directional selection would reduce the variation in adjacent chromosomal region, which was also called selective sweep and it is hard to find selective sweeps among three individuals. Secondly, long-term artificial selection has not occurred in tea population based on our population genetic analysis. Therefore, we turned into calculating the ratio of Ka/Ks among the ortholog genes of three genomes and try to discover some selection signatures at protein coding level. A total of 1600 and 135 genes with Ka/Ks > 1 were identified in DASZ-Shuchazao and DASZ-Yunkang 10, respectively (Supplementary Data 18-19; Supplementary Figure 21). The smaller number of DASZ-Yunkang 10 may be due to the low quality of gene annotation in Yunkang 10. Detailed gene annotation showed that some important genes may be under positive selection. For example, we identified a DFR-like (W06g014908) and an ANR-like (W13g026881) gene with Ka/Ks > 1 when comparing between DASZ and Shuchazao. Both DFR and ANR are involved in the biosynthesis of catechins. We added these results in Supplementary Note.

(2) The authors needed to further explore 1121 genes affected by 1135 high impact SVs, to explore the metabolic pathways and metabolites that these SVs and genes might influence, and the significance of these SVs and genes possibly involved in biotic and abiotic stresses or artificial domestication.
Thanks for the great comments. We further performed GO annotation on these genes and the results were similar to that of the genes affected by hSVs in DASZ. Approximately 50% of these 1,121 genes were related to the GO term ‘cellular process’ and ‘metabolic process’ (Supplementary Figure 13). Detailed functional annotation revealed that 10 R genes were lost in Shuchazao, which may be involved in the response to biotic stress (Supplementary Data 6). Furthermore, genes involved in important metabolic pathways which possibly relate to the tea quality traits were also identified. For example, two F3’5’H

genes, which encode isoforms of a key enzyme in the flavonoid pathway (W15g031524 and W15g031525) were partially lost in Shuchazao." We add the results in the revised manuscript (see line 188 to 197).

(3) What does the higher gene copy number (ANS, DFR, LAR, F3'5'H) mean in the tea genome?

Thanks for the question. The higher gene copy number of ANS, DFR, LAR, F3'5'H in the tea genome means that compared to Arabidopsis these gene families in tea plant have expanded during the process of evolution. As compounds of the flavonoid pathway, catechins are enriched in tea plants compared to other species. All four of these genes encode key enzymes in the flavonoid pathway (operating upstream of the reactions directly responsible for catechin synthesis) and the expansion of these genes may therefore underlie the enriched catechins in tea plants and the innovation and diversity of plant specialized metabolism.

To place the expansion and contraction of gene families in a wider genomic context, we additionally analyzed the number of genes contained in each orthogroup across 15 sequenced plant genomes. Orthogroups represent groups of genes derived from a single common ancestral gene. With this approach, we first inferred the size of the orthogroups across the 15 species[1,2,] including the three tea genomes, and we then compared the expansions and contractions of gene families with respect to the predicted size of the ancestral gene families across all nodes of the phylogeny[3,4]. In this manner, rather than comparing the number of gene copies between extant species, we looked at the statistically significant events of gene birth and loss across the lineages of the species phylogeny. This additional analysis is described in a new section of the manuscript (please see lines 331-389).

(4) The GWAS screened catechins and gallic acid synthesis related genes need be further tested for their function, such as in vitro enzymatic activity. The transcription factors regulating the formation of catechins also need be verified by corresponding transcriptional regulation experiments.

Thanks for your suggestion. Three of the candidate genes (CsANR, CsF3'5'H and CsMYB5) identified by GWAS were chosen for functional validation. On the one hand, we performed transient expression analysis of CsANR and CsF3'5H in tobacco leaves. On the other hand, CsANR and CsF3'5'H proteins were purified and incubated with tea extracts for 30 min in order to measure metabolic changes. Secondary metabolites were detected by LC-MS, and both of the two experiments showed that CsANR and CsF3'5'H could affect the contents of several flavonoid and anthocyanin related compounds including catechins (Figure 5; Supplemental Data 17). Moreover, we transiently expressed CsMYB5 in tobacco leaves to detect its regulation on the flavonoid pathway – again using direct metabolite measurements by LC-MS as the readout. It's clear that several flavonoid metabolites were significantly increased or decreased compared with the GFP only control.

(5) Further experiments are needed to verify the significance of GWAS screening for non-synonymous SNPs on the function of the gene, such as affecting enzyme activity or other attributes?

Thanks for the nice suggestion. To further verify the significance of GWAS screening for non-synonymous SNPs on the function of the gene, different alleles of CsANR and CsF3'5'H were tested. The purified enzymes of different alleles of both CsANR and CsF3'5'H were incubated with tea extracts, and their distinct catalytic function in tea secondary metabolism is presented in Figure 5b. Further *in vitro* assay indicated that both CsANRa and CsANRb could catalyze the conversion of cyanidin to epicatechin in the presence of the cofactor NADPH. Using the Arabidopsis AtANR as the positive control, we found that CsANRa had high Km and low enzyme efficiency in comparison with both of CsANRb and AtANR. These new data are discussed in lines 457 to 475.

Reviewer #2 (Remarks to the Author):

Manuscript 19-30844935 by Wen and colleagues presents a genome sequence for a Camellia sinensis accession performed at a very high quality. The manuscript also provides RNAseq data from numerous Chinese germplasm sources to identify SNPs that are used for diversity analyses. The data are useful, but the authors do not discover anything that was previously not known. Some results are not terribly believable, like the GWAS studies, and many of the conclusions are unjustified. I provide my specific comments below, in manuscript order.

We thank the reviewer for acknowledging the high quality of the manuscript we also appreciated the criticism which we believe that following a considerable concerted research effort we have been able to address. We think that the revised manuscript is considerably improved as a direct result of your comments. A detailed point-by-point discussion of your detailed comments follows below. Perhaps most critically we have provided validation for our GWAS story as well as provided answers to your questions as to how this was initially carried out which we trust will removes the ambiguities which concerned you.

Abstract:

(1) There is no such thing as a wild landrace. If it is a landrace, it is not wild. The idea, mentioned throughout, that landraces are somehow less subject to human selection is untrue and insulting to traditional farmers. Of course ancient farmers were selecting for improved traits. Most maize improvement, for instance, was performed by ancient farmers, and this is true for virtually all other crops. Hence, "the inconspicuous" sentence is nonsense. Moreover, if it so inconspicuous, then how did the authors perform GWAS

studies?

Thanks for the comments. We revised the manuscript according to the comments. We agree that “wild landrace” is not appropriate to describe DASZ. However, DASZ, which was found in wild condition at Mangyan Village, Yongjiang Township, Longyang District, Baoshan City, Yunnan Province (N 24°54′55.59″ E 98°48′30.87″; the altitude is between 1000 and 2000 meters; see Figure 1 as below) is an ancient tree and we thus describe it as an ancient tree in the revised manuscript.

In our study, we did not observe obvious difference between ancient trees and cultivars either at genetic or metabolic levels. Moreover, an earlier study also supported our results. This study collected 450 tea accessions including 331 landraces, 87 cultivars and 32 wild teas and we can clearly see the admixture among wild, landraces and cultivars from their barplot of population structure (Figure 2c)[5]. This large admixture indicates either an incomplete sampling of real wild forms, or teas cultivated today are not genetically distinct from their wild forms (this appears to be consistent with the trajectories of perennial tree domestication, given that tea is self-incompatible and propagated by cuttings), and the two alternatives are not mutually exclusive. We revised the manuscript to clarify this point (see line 27, 519-526, 539-547,572-575).

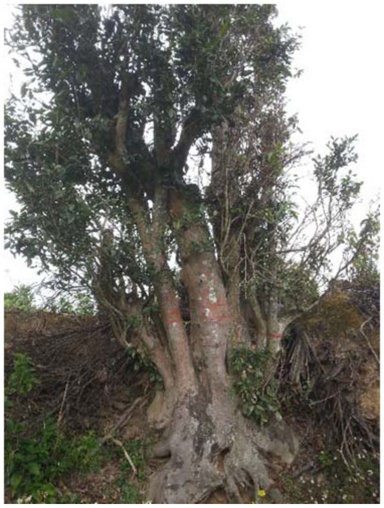

Fig. 1 Tea plant DASZ.

(2) Their analysis is not “a step of the genetic improvement” at all, and it is certainly not “significant” in that regard.

Thanks for the comments. We revised the manuscript according to them. However, we would like to summarize why we think this assembly is important also here.

Firstly, we assembled the first chromosome-scale genome of tea plant. A high-quality genome is important for genetic and biological study. As exemplified by metabolic traits or pathways, the quality of genome sequences is essential for the improved resolution of metabolic pathway genes. As some genes involved in specialized metabolism are physically clustered within the genome as tandem repeats or metabolic gene clusters, long read length technologies can resolve tandem duplications and extended repetitive sequences, revealing complex loci associated with specialized metabolism. In addition,

chromosome-scale genome enables syntenic analyses, which are vital for understanding genome evolution and dynamics, including the origins of gene duplications and gene clusters. In another example, as indicated in this study based on the assembled genome, we can use GWAS to locate QTL and discover genes responsible for the biosynthesis of catechins in tea plant. A total of 176 mQTL were identified by mGWAS, and candidate genes and favorable genetic variants were also detected. The function of several genes was verified as was the identification of superior and inferior alleles. We therefore contest that these results will indeed be an important foundation for future tea breeding.

Introduction:

(1) Why is the Scientific Data 2019 manuscript from Xia et al not mentioned? It has a more advanced Shuchazao assembly/annotation than does the 2018 manuscript that they reference.

Thanks for the comments. We add this reference in the revised manuscript. The complete and fragmented gene prediction BUSCO values of Shuchazao was 91.4% and 6.6% (PNAS 2018)[6]. The complete and fragmented gene prediction BUSCO value of Shuchazao was 86.2% and 8.2% (Scientific Data 2019)[7]. The complete and fragmented gene prediction BUSCO value of DASZ we report was 93.2% and 3.1%. The regression in quality is likely due to the use of only Arabidopsis data for gene finding within the Scientific data manuscript. The missing BUSCO of DASZ was slightly greater than Shuchazao based on PNAS 2018 (3.7% in DASZ; 2.0% in Shuchazao). However, the complete BUSCO of DASZ was greater than that reported for Shuchazao in either the PNAS 2018 or Scientific Data 2019 articles.

(2) Test from "For example, nanomaterials…." is a non-sequitur. There are lots of proposed benefits from tea, but little to no proof, and listing a couple extreme and unproven examples is not a good use of the reader's time.

Thanks for the comments. We removed the example of nanomaterials in the revised manuscript. The following sentence is added in the revised manuscript. "Nakayama et al. proved that tea polyphenols such as Epigallocatechin gallate (EGCG) and theaflavin digallate (TF3) could inhibit the infection of influenza virus by binding to haemagglutinin[8,9]. EGCG was not only prevent the infection of influenza virus, but also block the infectivity of other representative virus such as HCV, HIV-1 and HBV7[10]". As we are sure the reviewer will agree this is a far more compelling example.

(3) The idea that "the genetic diversity and population structure remain elusive due to limited number of molecular markers" is not true. You need fairly few markers for such an analysis, and certainly GBS marker sets are more than sufficient. Actually, tea diversity has been fairly well studied already, but that does not mean that every variety has been investigated. These authors choose some additional varieties, but I don't think they find much that is new in a general sense, just new information about these varieties.

Thanks for the comments. We agree that a small number of molecular markers may be

enough for illustrating the population structure and genetic diversity. However, most of the population genetic study on tea used SSR markers, which may be slightly outdated with the rise of the high throughput sequencing technology. The limited number of genetic markers (dozens of SSRs) may lose some information of minor alleles. The paper reported GBS marker sets (79,016 SNPs) of tea is enough but mainly focused on the tea accessions in Guizhou Plateau. We changed this sentence into “Most population genetic studies on diverse tea germplasm used SSR markers whilst a recent research used GBS marker sets (79,016 SNPs) to explore the genetic diversity which mainly focusing on the tea accessions in Guizhou Plateau. Therefore, the genetic diversity and population structure of tea remain elusive.” in the revised manuscript according to the reviewer’s comments.

Results:

(1) As mentioned above, comparisons should be made to the assembly in the Scientific Data assembly/annotation, not the two old publications.

Thanks for the suggestion. We made comparisons to the assembly and annotation in Scientific Data paper in the revised manuscript, see Table 1.

(2) Regarding 93.2% BUSCO scores, what were the BUSCO scores of earlier assemblies? I vaguely remember that the Scientific Data assembly gave something over 94%.

Thanks for the question. As we mentioned above 94% of BUSCO scores in Scientific Data includes the complete and fragment BUSCOs. 93.2% BUSCO scores in DASZ was the complete BUSCOs. When we added the fragment BUSCOs, BUSCO scores of DASZ is 96.3%.

(3) The whole SV description is vague. (What is tau S?) I am not expert in this type of analysis, but the authors’ statements of why homozygous SVs are possible artifacts or what hSVs mean (heterozygous I am guessing means hemizygous, at least for deletions). Are all SVs, as they define them, deletions, or do they include other types of rearrangements? Can they tell insertions from deletions, or are these all just considered indels?

Thanks for the comments. Tau S should be hSVs, it may be a typo during format transformation. We corrected this in the revised manuscript. Yes, heterozygous SVs indicate hemizygous ones we have added a comment to this effect in the Supplementary note. We compared the Pacbio reads with the DASZ reference genome to define the deletion and insertions. Detailed methods of how we detect SVs is now provided in the Supplementary Note.

(4) On lines 185-191, it seems odd that Fudingdabai is more closely related to Yunkang10 than to Shuchazao. Is Fudingdabai an assamica tea?

Fudingdabai is neither a typical assamica nor a sinensis type of tea. It is arbor type with

medium sized leaves. Yunkang 10 is typical assamica tea, an arbor type with large leaves. Shuchazao is typical senensis tea, a shrub with small leaves.

(5) It seems unlikely to me that the tea germplasm chosen was purely random. The authors see numerous relatives of Fudingdabai and a few others, but how do we know that this is an indication that many Chinese teas are related to these few lineages, or is it an outcome of what teas they chose to study? The results mentioned on lines 220-222 are strange, once again suggesting that the germplasm choice was not random.

Thanks for the comments. The accessions we studied here were randomly collected with the only consideration of geographic coverage and a few representative cultivars. Another study which conducted parentage analysis chose 128 elite tea cultivars and they found 29 accessions were the potential offspring of Fudingdabai and 15 of these 29 accessions were not included in our study[11]. Thus, two independent researches reached the similar conclusion that Fudingdabai played a crucial role in the breeding of Chinese tea, which partially rules out the effect of material selection. To clarify this question, we revised the manuscript and the following sentences were added. "In order to ensure the randomization of sampling, we compared our result with previous parentage analysis which selected 128 elite tea accessions and discovered that Fudingdabai played important role in Chinese tea breeding (the potential parent of 29 tea accessions)[11]. And 15 of these 29 accessions were not included in our study. The importance of Fudingdabai in the Chinese tea breeding is therefore strongly indicated by these two independent studies."

(6) Regarding lines 236-237, how do the authors explain that other researchers have seen correlations both with geography (that is, Yunnan versus elsewhere) and with assamica (large leaf) versus sinensis (small leaf)? My guess is that their germplasm collection was so skewed (perhaps to small leaf varieties) that there really was little leaf size variation, and thus no real variance investigated.

Thanks for the comments. First, here not all the accessions from different provinces showed random distribution in phylogenetic tree. We still found a subset of accessions originating from the same province grouped together. This phenomenon was also observed in other population genetic studies[11]. However, as the reviewer mentioned, some studies still showed a better clustering pattern than our study. For example, Yao et al. reported that a population genetic study using 450 tea accessions[5]. They showed almost all the accessions from Yunnan province grouped together in the phylogenetic tree. One possible reason we suspect is that most of the accession they used were landraces and wild accessions (80.7%)[5]. However, in our study, most accessions are cultivars (73.8%). Landraces and especially wild accessions would be more related to geographic distribution. The typical assamica (CSA) was arbor with large leaves, and the typical sinensis (CSS) was shrub with small leaves. However, many accessions are intermediate

types, for example, shrub with large leaves or arbor with small leaves. If we strictly classified according to the typical traits, CSS and CSA will have a better clustering in phylogenetic tree (Figure 2 d).

(7) The metabolite analyses are only valid if performed on leaves harvested at the same leaf size, on the same day, and in a single field location. From the Methods, it seems unlikely that any of this was actually done.
Thanks for the comments. All the 176 tea accessions that metabolite analyses performed on are at the same tree age and grown in a single field location. We harvested all the leaf samples using liquid nitrogen in the same day. It is difficult to ensure the leaf size are exactly the same for each accession as the leaf trait is very diverse among tea germplasm. However, we did ensure the sampled leaves are at the same developmental stage. We have revised the description in the methods to clarify this important point and are thankful that you brought it to our attention.

(8) Gene family analyses have already been done in other publications.
Thanks. In an attempt to bring new light on the evolutionary dynamics of gene families in tea, we specifically looked at the rapidly evolving gene families across the branches of the species phylogeny, and this allowed us to identify the gene families which are differentially evolving between the three tea genomes sequenced so far. This gave us the possibility to analyze evolutionary expansions and contractions which were either exclusive to DASZ or shared by all three genomes of tea (DASZ, CSA and CSS), and also to locate where the events of contractions and expansions took place along the phylogeny, e.g., in the terminal branches (thus species-specific) or before the species diverged (Supplementary Figure 19). We hope that this explains why we feel there is additional value in this analysis compared to what has been provided before.

(9) GWAS analysis is not valid unless the metabolite studies are done with samples collected in the same field, at the same leaf stage on the same day for all of the germplasm. I expect that this was not done. Many studies have shown major contributions of the environment to the metabolite profiles observed. Moreover, favorable genetic variants are only possible to detect if you have strong GWAS results. GWAS is prone to lots of false positives, as well, but it is hard to see how the authors took this into account.
Thanks for the comments. As we mentioned above, all the accessions were grown in the same field and all the samples were at the same leaf stage. We validated the GWAS results using both in vitro enzyme activity assay and transient overexpression. As mentioned above we revised the manuscript for better clarification of this important issue.

Discussion
(1) The sentence “This huge difference….” Is not a very likely explanation. Population

history (e.g., time spent homozygous versus heterozygous) is a more likely explanation.

Thanks for the comments. The huge differences numbers of hSVs between DASZ and Shuchazao may result from many reasons. We revised the manuscript as follows to make the explanation clearly. The huge difference of hSV number between DASZ and Shuchazao may result from several factors. On one hand, different number of hSVs may be partially caused by different Pacbio sequencing depth, which was less in Shuchazao than DASZ. On the other hand, a previous study showed that population history and clonal propagation tended to accumulate recessive deleterious variants in a heterozygous state[12]. Shuchazao was selected from local varieties of Shucheng County in the 1990s[13] while as an ancient tree that has lived several hundred years DASZ was more inclined to hide the SVs in a heterozygous state compared with Shuchazao.

(2) On lines 409-411, the conclusion made has already been shown to not be true in a general sense. The sinensis and assamica subspecies can be easily separated, and have shown little intercrossing, although that does not seem to be true for the apparently more skewed germplasm these authors chose to study.
Thanks for the comments. As we mentioned above, we did not say landraces were less subjected by human selection and Yao et al. also reported similar results[5]. Also, as we mentioned above, the typical sinensis and assamica accessions clearly separated in a phylogenetic tree. However, many accessions are intermediate type, for example the hybrids of typical assamica and typical sinensis, which is also confirmed by the breeding record (see Supplemental Data 7).

(3) Lines 421-433 are interesting, but not really related to this study.
Thanks for the comments. In lines 421-433 we are trying to explain why tea has not undergone long-term artificial directional selection in terms of metabolites that confer the flavor. This study aims to provide genetic, biochemical and evolutionary insights into tea plant based on a newly assembled reference genome and diverse tea germplasm collected here. These sentences are speculations based on the findings in this study, which would be helpful for tea breeding practices and raise an open question for further basic biological study on tea plant. As such we would rather keep them in the manuscript. However, if the reviewer is still not convinced by their relevance we would, naturally, remove them.

(4) Lines 434-437 are quite reasonable, but the authors should avoid the term “wild teas” because this is not a good name for landraces. There is nothing wild about them. Also, the authors should provide any proof for trees that have been harvested for hundreds of years. Is there any documentation of this, or is it just the opinion of local farmers.
Thanks for the comments. We agree that the term “wild teas” is not appropriate. As we also mentioned above the ancient trees, we collected here are all found in wild condition, which are ancient as evidenced from the status of these trees. Photos of these trees are shown in Figure 2 below. We add a reference to prove for trees have been harvested for

hundreds of years (Tea Plant Verities in China 2001)[13].

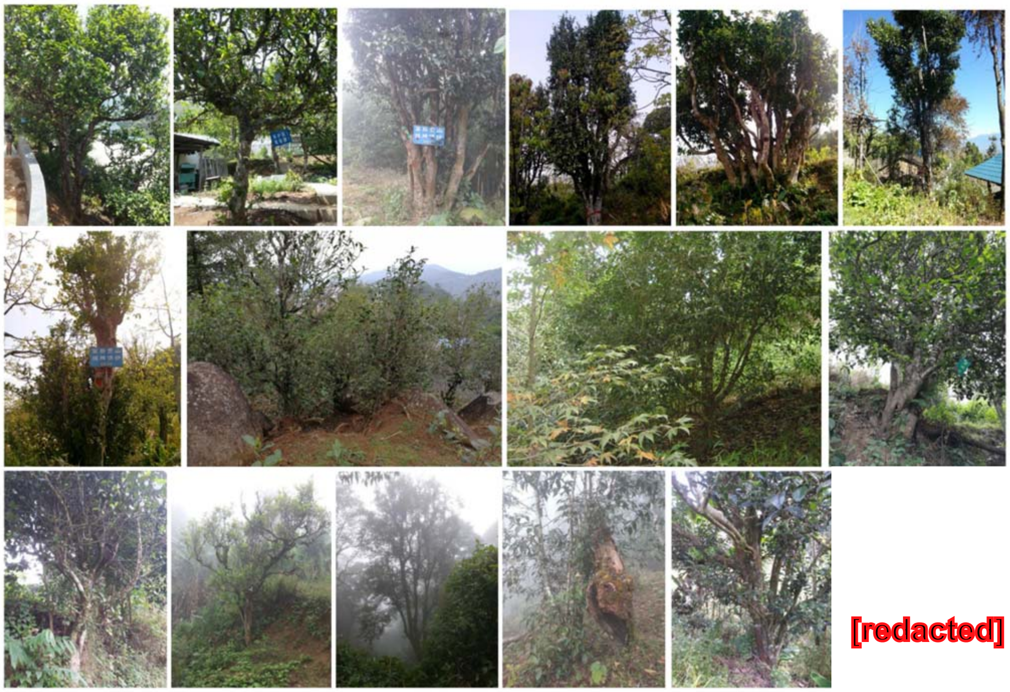

Fig 2. Pictures of tea trees collected in this study.

(5) “and the protective effects of tea catechins on human health has been finely documented” greatly overstates the case. The possible value of catechins on human health if far from proven, much less “finely documented”.

Thanks for the comments. The beneficial effects of tea catechins have been extensively studied and published (we show just a few references below). Maybe these are mostly in vitro studies, or based on various cell models. However, in pre-clinical studies the evidences that catechins have antioxidant effects are solid, however we agree that in clinical trials their protective effect is definitely more difficult to assess due to lack of large–scale cohort studies. To be more precise we revised the manuscript according to your comments. The word “finely” has been removed.

Steinmann, J., Buer, J., Pietschmann, T. & Steinmann, E. Anti-infective properties of epigallocatechin-3-gallate (EGCG), a component of green tea. British journal of pharmacology 168, 1059-1073 (2013).

Lixia Chen, Huanbiao Mo, Ling Zhao, Weimin Gao, Shu Wang, Meghan M. Cromie,

Chuanwen Lu, Jia-Sheng Wang, Chwan-Li Shen, Therapeutic properties of green tea against environmental insults, The Journal of Nutritional Biochemistry, Volume 40, 2017, Pages 1-13, ISSN 0955-2863, https://doi.org/10.1016/j.jnutbio.2016.05.005.
Wen-Hsing Cheng, Green Tea: An Ancient Antioxidant Drink for Optimal Health?, *The Journal of Nutrition*, Volume 149, Issue 11, November 2019, Pages 1877–1879, https://doi.org/10.1093/jn/nxz187
Martha J. Shrubsole, Wei Lu, Zhi Chen, Xiao Ou Shu, Ying Zheng, Qi Dai, Qiuyin Cai, Kai Gu, Zhi Xian Ruan, Yu-Tang Gao, Wei Zheng, Drinking Green Tea Modestly Reduces Breast Cancer Risk, *The Journal of Nutrition*, Volume 139, Issue 2, February 2009, Pages 310–316, https://doi.org/10.3945/jn.108.098699

Reviewer #3 (Remarks to the Author):

In this report, Qiu et al. present a new reference genome sequence for tea, based on DNA from a landrace, contact and genetic maps, and long-read technologies. Additionally, RNA sequencing was performed on other accessions, and the authors used this information to investigate the history of cultivated varieties, as well as to find genes implicated in biosynthesis of important compounds. The authors concluded that flavor metabolites may not have been strongly targeted by selection.
The first interest of this manuscript is the quality of the genome sequence, particularly the continuity, which is a great improvement compared to the two draft versions already published. There are however no main findings derived from this high-quality assembly compared to the previous papers. The structural variations led to some conclusions that should be investigated more in-depth. The parentage study has to be improved, as well as the investigation of gene categories enrichment and percentage of variations between genomes. Taken together, the authors should investigate more their preliminary findings to show more convincingly the interest of this new reference.

We thank the reviewer for the comments concerning the quality of the genome. We have taken the comments regarding novelty very seriously and made a considerable number of improvements to the investigation of the gene categories as well as validating the GWAS analysis as a result we feel that the study has been dramatically improved and thank the review for providing the impetus and direction for us to be able to achieve this.

Table 1 presents a comparison of annotation results, and it appears that there is no strong improvement compared to the sequence of Shuchazao besides the continuity of the contigs. The authors may make a better case why this sequence is really helping to address more questions than the previous one.
The other two genome of tea, scaffolds were not anchored to the chromosome which is of major importance for multiple analyses. With the help of Hi-C data we anchored 99.55% sequences to the chromosome. A high-quality genome is important for genetic and

biological study. As exemplified by metabolic traits or pathways, the quality of genome sequences is essential for the improved resolution of metabolic pathway genes. As some genes involved in specialized metabolism are physically clustered within the genome as tandem repeats or metabolic gene clusters, long read length technologies can resolve tandem duplications and extended repetitive sequences, revealing complex loci associated with specialized metabolism. In addition, chromosome-scale genome enables syntenic analyses, which are vital for understanding genome evolution and dynamics, including the origins of gene duplications and gene clusters. Another example, as indicated in this study based on the assembled genome, we can use GWAS to locate QTL and discover genes responsible for the biosynthesis of catechins in tea plant.

The analysis of SVs led to the finding of a large number of homozygous cases (line 144), which should be artefactual. It is important to sort out what is the problem. An assessment of error rates in long-read assemblies would be helpful. Many other conclusions, in particular about the heterozygous SVs differences with other varieties may be affected by this problem.
PacBio data may have uncorrectable errors and we used Illumina HiSeq data to evaluate the long-read assembly quality. A total of 201,175 incorrect base were identified and the base accuracy was 99.9936%.

The parentage analysis is based on a selection of 500 random variants. Many conclusions are apparently based on this, although this number looks rather low for such a large genome. It is essential in this case to show that the same results can be obtained by other assortments of random markers.
Thanks for the comments. We repeated the random marker selection 10 times, and the parent-offspring pairs were only identified in at least 9 marker sets were kept for further analysis. We revised the results of parentage analysis according to the new results, see line 232 to 236.

Line 152, the category “regulation of cell death” is deemed enriched in heterozygous SVs. The authors should investigate this, and maybe other categories, in detail to derive functional insights.
Thanks for the comments. A total of 27 genes were in the GO term ‘regulation of cell death’ and detailed GO analysis showed that about half of them were related to the membrane and displayed catalytic activity. Approximately 80% of them were related to the term ‘response to stimuli’ (Supplementary Figure 14). W05g013121 and W09g020593 exhibited high expression levels in multiple tissues of DASZ, while most of the 27 genes maintained low expression levels in all tissues (Supplementary Figure 15). W05g013121 and W09g020593 were annotated as a heme-binding protein and an aspartyl protease, respectively and these two protein families may relate to the autophagy pathway in plants. We added these results to the revised manuscript (see line 165 to 177).

In the discussion, the huge difference in SVs between Shuchazao and DASZ is explained by growth time. Other explanations have to be investigated, first the impact of the sequencing technologies, but also the sizes of the populations.

Thanks for the comments. To clarify this question, we added the following sentence in the revised manuscript. "The huge difference of hSV number between DASZ and Shuchazao may result from several factors. On one hand, different number of hSVs may be partially caused by different Pacbio sequencing depth, which was less in Shuchazao than DASZ. On the other hand, a previous study showed that population history and clonal propagation tended to accumulate recessive deleterious variants in a heterozygous state[12]. Shuchazao was selected from local varieties of Shucheng County in 1990s[13] while as an ancient tree that has lived several hundred years DASZ was more inclined to hide the SVs in a heterozygous state compared with Shuchazao".

Reviewer #4 (Remarks to the Author):

In this manuscript, the authors report a new assembly of tea plant (Camellia sinensis), resequencing analysis based on RNA-seq data form 217 accessions. Compared with the original genome, the quality of this assembly is much higher. Chromosome-level genome assembly is important for understanding the origin of tea tree species, genome-assisted breeding, and genomic structure analysis. The authors used SNPs obtained from the sequences of leaf transcriptomes from 217 accessions to perform population genomics analysis, explore phylogenetic relationships of tea trees and perform genomic association analysis.

The manuscript is particularly useful for generating a genetic resource for the tea plant research community. I believe this genome assembled version will be the best genome assembled version of the species for quite some time. As a reference genome of Camellia sinensis, authors need to be more careful in confirming the quality of the assembly of the genome, especially in comparison with published assemblies, and in-depth analysis of the new assembly.

Based on the reference genome, the author analyzes SV in the genome, but I think this analysis is open to question, both in terms of method and conclusion. Considering the large size of tea tree genome, in order to speed up and save costs, the author used RNA-seq data for SNP calling. These data also need to be verified to see if the results obtained by other methods are good consistency. The manuscript is in general clearly written.

Considering that tea tree has already released at least 2 different genome assemblies, the author needs to better show the uniqueness of this version and analyze more biological questions, so I suggest that this article needs a major revision.

We thank the reviewer for their enthusiasm about the quality of our genome sequence and

also for the more critical comments regarding SV analysis and uniqueness of the biology. These comments led us to carry out considerably more experimentation and data analysis and as a result we feel our manuscript has been dramatically improved.

There are other major issues that require attention:

1. Considering the complexity of the tea genome, especially the high content of repetitive sequences (over 80%), I strongly recommend that the author use different assembly strategies and software to list different assembly results and then determine a version, rather than using Falcon directly. HERA (Huilong Du,2019) is much better for high repetitive sequences genome. Cuna is other choice which is reported more accurate. Considering that this manuscript is based on genome assembly updates, the author is strongly recommended to use multiple software for assembly, and fully explain that this version is the best.
Thanks for the suggestions. It seemed that the PacBio tailored FALCON gave good contiguity which is in line with e.g. results obtained by Tean et al. reported that the continuity of FALCON assembly was better than Canu[15]. That said, there is always a balance in strength and weaknesses of different assemblers. We checked carefully of previously published tea genome including the data in preprints which suggested that our genome assembly version was the best. It will be definitely interesting to test the new HERA assembler in the future, especially as this promises one toolchain for assembly and incorporation of Hi-C/optical mapping data.

2. Hi-C data is useful when the chromosomes anchoring, but in the current high-quality plant reference genomes, Bionano data is also used to correct each other with Hi-C. Based on our past experience, simply using Hi-C in highly heterozygous genomes will lead many assembly errors, so it is recommended that the author use Bionano data for quality improvement, especially comparing the differences between the results of Bionano de novo assembly and the existing version.
Indeed, Bionano could be conducted to correct some assembly errors. However, in the case of high heterozygous tea genomes, bionano approach based on enzyme cleavage sites has caused some problems. Indeed this seems to be the case here, as we have tried constructing Bionano libraries three times. Unfortunately, the results of enzyme cleavage were not good enough for sequencing. In addition, concerning the accuracy of Hi-C, we combined the genetic map and Hi-C data to correct and archive a more accurate chromosome-scaled reference genome.

3. High-quality reference genomes have large moderators for annotations of TEs sequences. In fact, the authors found that more than 87% of the tea tree genome is composed of TEs sequences. I hope that the author can analyze the TEs sequence, not just show the results of the annotation.
Thanks for the suggestion. By further analysis we found that Gypsy and Copia played dominant roles in LTRs, accounting for 49.36% and 8.50% of the DASZ genome,

respectively (Supplementary Table 9). The proportions of Gypsy and Copia were similar but slightly higher than the other two genomes of tea. While considerable higher contents of other LTR sequences were detected in DASZ than Shuchazao (505,322,326 bp vs 162,745,061 bp; Supplementary Table 9), and this difference may be due to the higher Pacbio sequencing depth of DASZ than Shuchazao, which could facilitate the prediction of long repetitive sequence.

4. The authors used Pacbio reads mapping for SV calling and analysis, but these analyses were based on only one individual and the reference genome. Both the method and the biological interpretation were very inadequate. It is recommended to use more individuals, especially key accessions among 217 accessions.

Thanks for the comments. Considering the budget it is difficult for us to sequence more accessions using Pacbio or ONP. Thus, we focused on the biological function of genes affected by SVs. We performed GO annotation to these genes and the results were similar to the genes affected by hSVs in DASZ. Approximately 50% of 1,121 genes were relating to the GO term ‘cellular process’ and ‘metabolic process’ (Supplementary Figure 13). Detailed functional annotation revealed that 10 R genes were lost in Shuchazao, which may be involved in the response to biotic stress (Supplementary Data 6). Furthermore, genes involved in important metabolic pathways which possibly relate to the tea quality traits were also identified. For example, two F3’5’H genes, which are key enzymes in flavonoid pathway (W15g031524 and W15g031525) were partially lost in Shuchazao. (see line 188 to 197).

5. In the analysis of the population genomics, due to the complex history of tea tree domestication, there are many asexual reproduction phenomena, and many varieties have the same name or are recorded incorrectly. How did the author ensure the accuracy of these samples?

Thanks for the comments. Indeed, many tea varieties are clonal cultivars. In the past decades, clonal cultivars played important roles in the Chinese tea industry. All the cultivars were registered or documented (see Supplementary Data 7). Although, based on SNP data, relatively few (32) of 217 accessions showed high similarities with other accessions (identity > 95%). We cannot easily exclude that these accessions as incorrectly recorded. Some of them could be the result of natural mutation. However, even if we removed these accessions; the main conclusion of our study would not change. Besides, their study is actually a reflective description of the current status of tea breeding.

6. The author uses RNA-seq sequences for analysis. According to the statistics of the data submitted by the author, the data volume of the sample ranges from 14M reads to 93M reads, and the mapping rate ranges from 80% to 98%. The author needs to explain the impact of these differences on their SNP calling. In addition, SNP filtering is very tricky. It is recommended that the author analyze each step of SNP calling and then perform

SNP filtering based on the results.

Thanks for the comments. The low mapping rate of some samples may result from contamination during library construction or other reasons. Moreover, considering the high heterozygosity of tea plants, we set a very strict threshold for SNP filtering. After standard filteration of GATK, for homozygous SNPs, the supporting reads should be more than 10, for heterozygous SNPs, the sequencing depth for each allele should be greater than 4, and SNPs within 10 bp of InDels were removed. In order to examine the accuracy of SNP calling and filtration, we randomly peaked 500 SNPs and 2 accessions for sanger sequencing. The results showed that the accuracy of SNP calling was 98.5%. We added this result in the revised manuscript (see lines 213-215).

7. As for this species, unlike Camellia sinensis (Linn.) Var. assamica, the leaf size changes little. The author divides the leaf size into three types. Is there any specific phenotypic measurement data support? Is this phenotype related to different growth environments? If the phenotype is not accurate, I recommend removing it in subsequent GWAS analysis.

Thanks. Leaf size is not used as phenotype in the GWAS analysis in this study.

8. Considering the high heterozygosity of the tea genome, part of the parentage analysis, it is recommended to use all biaslelic SNPs to ensure the credibility of the structure, in addition to the small number of samples, this part of the analysis results need more methods to support its feasibility. 38 samples have known breeding records. According to the results of population genomics analysis, can they be consistent with these breeding records?

I hope the author can explain and elaborate on the above issues, and more importantly, improve the quality of the manuscript and scientific issues through our discussion.

Thanks for the comments. We repeated random marker selection 10 times, and only the parent-child pairs that were identified in at least nine marker sets were retained for further analysis. Based on this analysis 27 parent-offspring pairs were consistent with the breeding record. The results were indicated in the revised manuscript see line 232 to 236.

Related References

1 Emms, D. M. & Kelly, S. OrthoFinder: solving fundamental biases in whole genome comparisons dramatically improves orthogroup inference accuracy. *Genome Biol* **16**, 157, doi:10.1186/s13059-015-0721-2 (2015).

2 Emms, D. M. & Kelly, S. OrthoFinder: phylogenetic orthology inference for

comparative genomics. *Genome Biol* **20**, 238, doi:10.1186/s13059-019-1832-y (2019).

3 Hahn, M. W., De Bie, T., Stajich, J. E., Nguyen, C. & Cristianini, N. Estimating the tempo and mode of gene family evolution from comparative genomic data. *Genome Res* **15**, 1153-1160, doi:10.1101/gr.3567505 (2005).

4 De Bie, T., Cristianini, N., Demuth, J. P. & Hahn, M. W. CAFE: a computational tool for the study of gene family evolution. *Bioinformatics* **22**, 1269-1271 (2006).

5 Yao, M.-Z., Ma, C.-L., Qiao, T.-T., Jin, J.-Q. & Chen, L. Diversity distribution and population structure of tea germplasms in China revealed by EST-SSR markers. *Tree genetics & genomes* **8**, 205-220 (2012).

6 Wei, C. *et al.* Draft genome sequence of Camellia sinensis var. sinensis provides insights into the evolution of the tea genome and tea quality. *Proceedings of the National Academy of Sciences* **115**, E4151-E4158 (2018).

7 Xia, E. *et al.* The tea plant reference genome and improved gene annotation using long-read and paired-end sequencing data. *Scientific data* **6**, 1-9 (2019).

8 Nakayama, M. *et al.* Inhibition of the infectivity of influenza virus by tea polyphenols. *Antiviral research* **21**, 289-299 (1993).

9 Nakayama, M., Toda, M., Okubo, S. & Shimamura, T. Inhibition of influenza virus infection by tea. *Letters in applied microbiology* **11**, 38-40 (1990).

10 Steinmann, J., Buer, J., Pietschmann, T. & Steinmann, E. Anti-infective properties of epigallocatechin-3-gallate (EGCG), a component of green tea. *British journal of pharmacology* **168**, 1059-1073 (2013).

11 Tan, L.-Q. *et al.* Fingerprinting 128 Chinese clonal tea cultivars using SSR markers provides new insights into their pedigree relationships. *Tree genetics & genomes* **11**, 90 (2015).

12 Zhou, Y., Massonnet, M., Sanjak, J. S., Cantu, D. & Gaut, B. S. Evolutionary genomics of grape (Vitis vinifera ssp. vinifera) domestication. *Proceedings of the national academy of sciences* **114**, 11715-11720 (2017).

13 Yang, Y. Y. Y. *Tea Plant Varieties in China*. (shanghai scientific and technical publishers, 2001).

14 Chin, C.-S. *et al.* Phased diploid genome assembly with single-molecule real-time sequencing. *Nature methods* **13**, 1050 (2016).

15 Teh, B. T. *et al.* The draft genome of tropical fruit durian (Durio zibethinus). *Nature genetics* **49**, 1633 (2017).

Reviewer #1 (Remarks to the Author):

The revised version is significantly improved; the authors performed some of the suggested experiments whose results are consistent with the previous GWAS results.
Nevertheless there are still some issues that I am going to point out below.

1) In line 381, whether these DASZ-specific orthogroups confer the DASZ different phenotypes from other varieties, such as different cell sizes，numbers or other traits？
2) In lines 433-450, CsANR, CsF3’5’H and CsMYB5 all have more than two non-synonymous SNPs, but only two of them are shown in Figure 5, and there is no explanation in the legend. The authors should explain how to choose the two alleles of each of the three genes for next function validation.
3) In lines 721-723, cDNA is obtained by synthesis instead of being amplified from the tea tree. Does it reflect the real situation? As far as I know, the cDNA amplification of some genes in the tea tree does not match the sequencing results.
4) In Figure 5, ANR protein is transiently expressed in tobacco leaves or extracted from tea, and its effect on metabolites is partially inconsistent. How to explain it?
5) In line 433, I suggest that “10 non-synonymous” revised as “ten non-synonymous” to make it consistent with the following description.
6) In line 353, the brackets may be missed. In line 732, the spaces may be missed.

Reviewer #2 (Remarks to the Author):

The revisions made to the manuscript seem absolutely correct to me. I do not see any need for further revision.

Reviewer #3 (Remarks to the Author):

The authors adequately answered to my concerns. The quality of the genome sequence is better evaluated, and detailed analyses now illustrate its interest. As this new resource may be of general interest for tea research and plant genome evolution, I recommend publication.

Reviewer #4 (Remarks to the Author):

Thanks to the author for patiently replying to most of my questions, especially in time during the epidemic. But there are still some questions that need to be answered clearly.

1) The core of this study is high-quality genome assembly. Authors should not ignore comparisons with recently published genome assemblies(3.36Gb in doi: https://doi.org/10.1101/2020.03.19.998393, and 2.92Gb in doi: https://doi.org/10.1101/2020.01.02.892430) of the same species. The author should carefully compare the differences between these different assemblies and provide enough results to support the genome assembly version of this study is good enough. BUSCO and re-mapping are not enough to explain the differences between the assemblies. I strongly recommend the author to compare the sequence fragments or contigs between different assembled versions. If necessary, the author can modify and extend the contigs based on different assembly results.
2) Based on our experience in plant genome researches, we find that the annotation of protein-coding genes is very tricky. As the author's description and Supplementary Figure 7, repeat-masked genome was used as in-put for ab initio prediction. This method usually works in simple genomes, but for

complex genome, it will make a lot of massing. In fact, the protein-coding genes in the three assemblies are different (33k~40K). Authors should try more annotation methods and parameters and provide reliable annotations, and make a comprehensive comparison with published annotations.

REVIEWER COMMENTS

Reviewer #1 (Remarks to the Author):

The revised version is significantly improved; the authors performed some of the suggested experiments whose results are consistent with the previous GWAS results.
Nevertheless there are still some issues that I am going to point out below.

1) In line 381, whether these DASZ-specific orthogroups confer the DASZ different phenotypes from other varieties, such as different cell sizes，numbers or other traits？
Thanks for the comments. DASZ-specific orthogroups encoding several genes related with cell signaling and division, and cell wall metabolism and some of them may have important biological function. For example, OG0003683 was an orthogroup of TEBICHI gene family, which is required for DNA replication, recombination and T-DNA integration[1-3]. The mutant of TEBICHI gene in Arabidopsis will result in morphological defects, such as incorrect organ formation and defects in meristem maintenance[1,2]. However, we did not observe specific unusual phenotypes in DASZ. It is clearly difficult (impossible) to relate expansions or contractions of gene families to the differential phenotypes that could characterize DASZ with respect to the other tea genomes analyzed here. The size of the gene family is only one - out of many factors - which could affect a given phenotype. The level of expression, of course, the existence of various types of polymorphisms impacting the gene function, and also the presence of genetic and epigenetic changes affecting the overall transcriptional regulation, are all factors which could lead to the emergence of a novel phenotype.

2) In lines 433-450, CsANR, CsF3'5'H and CsMYB5 all have more than two non-synonymous SNPs, but only two of them are shown in Figure 5, and there is no explanation in the legend. The authors should explain how to choose the two alleles of each of the three genes for next function validation.
Thanks for the suggestion. We first tested the contribution of each non-synonymous SNP in each gene to the metabolic trait by ANOVA and only significant SNPs were kept for further analysis ($P < 0.05$). Due to the heterozygosity of tea genome, we evaluated the function of each genotype composed of these significant non-synonymous SNPs (Figure 4b-d). Accessions with genotypes of high and low level phenotypic contribution were selected to amplify the cDNA for each gene, respectively. The PCR products were subsequently introduced into T-vectors and sequenced to select the allele for each gene. Because each gene harbored more than three alleles (Figure 4b-d), for simplicity, we selected alleles with high and low level of phenotypic contribution for further functional validation, respectively. We

marked the selected alleles in Figure 4, attached allele sequences in Supplementary Note and revised the manuscript and figure legend to make this part more clearly, see line 458 to 463, line 724 to 730.

3) In lines 721-723, cDNA is obtained by synthesis instead of being amplified from the tea tree. Does it reflect the real situation? As far as I know, the cDNA amplification of some genes in the tea tree does not match the sequencing results.

Thanks for the comments. As we mentioned above, cDNA of each of the three genes were amplified from tea accessions and the two alleles for each gene were studied based on the sequence results of TA-clones. We had already constructed the vectors of each of the three genes for functional validation. Due to the COVID-19 pandemic the experiments were severely influenced and the vectors cannot be sent out from Wuhan. Our colleagues in Max Planck Institute in Germany performed the subsequent experiments in Germany. They synthesized these genes according to the sequenced allele sequences for the following functional validation.

4) In Figure 5, ANR protein is transiently expressed in tobacco leaves or extracted from tea, and its effect on metabolites is partially inconsistent. How to explain it?

Thanks for the comments. The results of enzyme assay were largely dependent on the concentration and composition of substrates. The metabolic system in tobacco and tea is not exactly the same. Usually the same enzyme could not lead to same metabolic output in different organisms. Moreover, transient expression is *in vivo* assay and incubation with tea extract is *in vitro* assay. Therefore, as shown in Figure 5c ANR transiently expressed in tobacco leaves and incubated with tea extracts had partially inconsistent effect on metabolites. However, both experiments indicate consistent trends of some key metabolites. For example, the contents of anthocyanin A11 were increased in both *in vivo* and *in vitro* assays.

5) In line 433, I suggest that “10 non-synonymous” revised as “ten non-synonymous” to make it consistent with the following description.

Thanks for the comments. We revised manuscript according to the comments.

6) In line 353, the brackets may be missed. In line 732, the spaces may be missed.

Thanks for the comments. We revised manuscript according to the comments.

Reviewer #2 (Remarks to the Author):

The revisions made to the manuscript seem absolutely correct to me. I do not see any need for further revision.

Reviewer #3 (Remarks to the Author):

The authors adequately answered to my concerns. The quality of the genome sequence is better evaluated, and detailed analyses now illustrate its interest. As this new resource may be of general interest for tea research and plant genome evolution, I recommend publication.

Reviewer #4 (Remarks to the Author):

Thanks to the author for patiently replying to most of my questions, especially in time during the epidemic. But there are still some questions that need to be answered clearly.

1) The core of this study is high-quality genome assembly. Authors should not ignore comparisons with recently published genome assemblies (3.36Gb in doi: https://doi.org/10.1101/2020.03.19.998393, and 2.92Gb in doi: https://doi.org/10.1101/2020.01.02.892430) of the same species. The author should carefully compare the differences between these different assemblies and provide enough results to support the genome assembly version of this study is good enough. BUSCO and re-mapping are not enough to explain the differences between the assemblies. I strongly recommend the author to compare the sequence fragments or contigs between different assembled versions. If necessary, the author can modify and extend the contigs based on different assembly results.
Thanks for the information of the two genome assemblies. Although these assemblies are of the same species, they are of tea varieties different from the one assembled here, which may not be feasible to modify and extend the contigs based on different assembly results as the reviewer suggested. And more importantly, we found the genomic data of both studies are not publicly available. We thus could not perform the comparisons.

2) Based on our experience in plant genome researches, we find that the annotation of protein-coding genes is very tricky. As the author's description and Supplementary Figure 7, repeat-masked genome was used as in-put for ab initio prediction. This method usually works in simple genomes, but for complex genome, it will make a lot of massing. In fact, the protein-coding

genes in the three assemblies are different (33k~40K). Authors should try more annotation methods and parameters and provide reliable annotations, and make a comprehensive comparison with published annotations.

Thanks for the comments. The annotation methods and parameter we used are widely adopted in genome annotation studies. Repeat-masked genome is commonly used in many complex genomes, such as maize[4] and wheat[5] and this step is also recommended in maker pipeline[6]. It is difficult to compare annotations with these two studies. On one hand, the annotation data of the two studies are not released. On the other hand, the description of annotation pipeline of the two studies is not detailed. Moreover, compared with them, we integrated Pacbio ISO-seq data in our gene annotation pipeline which may improve the annotation quality of DASZ.

References

1 Inagaki, S. *et al.* Arabidopsis TEBICHI, with helicase and DNA polymerase domains, is required for regulated cell division and differentiation in meristems. *The Plant Cell* **18**, 879-892 (2006).

2 Inagaki, S., Nakamura, K. & Morikami, A. A link among DNA replication, recombination, and gene expression revealed by genetic and genomic analysis of TEBICHI gene of Arabidopsis thaliana. *PLoS genetics* **5** (2009).

3 Van Kregten, M. *et al.* T-DNA integration in plants results from polymerase-θ-mediated DNA repair. *Nature plants* **2**, 1-6 (2016).

4 Yang, N. *et al.* Genome assembly of a tropical maize inbred line provides insights into structural variation and crop improvement. *Nat Genet* **51**, 1052-1059, doi:10.1038/s41588-019-0427-6 (2019).

5 Appels, R. *et al.* Shifting the limits in wheat research and breeding using a fully annotated reference genome. *Science* **361**, eaar7191 (2018).

6 Holt, C. & Yandell, M. MAKER2: an annotation pipeline and genome-database management tool for second-generation genome projects. *BMC bioinformatics* **12**, 491 (2011).

Reviewer #1 (Remarks to the Author):

The authors adequately answered to my concerns. The manuscript can be considered for publication.

Reviewer #4 (Remarks to the Author):

Thanks to the author's patience for replying. In this version, the author made a good modification. I have no more questions. I hope that the results of this research can be published as soon as possible.

REVIEWERS' COMMENTS:

Reviewer #1 (Remarks to the Author):

The authors adequately answered to my concerns. The manuscript can be considered for publication.

Thanks a lot for the positive assessment and nice suggestions which we feel greatly aided us to improve the manuscript.

Reviewer #4 (Remarks to the Author):

Thanks to the author’s patience for replying. In this version, the author made a good modification. I have no more questions. I hope that the results of this research can be published as soon as possible.

We thank the reviewer for the critical comments which led us to carry out considerably more experimentation and data analysis and as a result we feel our manuscript has been dramatically improved.
